# Site Disorder Drives Cyanide Dynamics and Fast Ion Transport in Li_6_PS_5_CN

**DOI:** 10.1021/acs.chemmater.4c00979

**Published:** 2024-09-25

**Authors:** Connor E. Ray, Yi Yao, Shelby L. Galinat, Bennett Addison, Volker Blum, Annalise E. Maughan

**Affiliations:** † Department of Chemistry, Colorado School of Mines, Golden, Colorado 80401, United States; ‡ Thomas Lord Department of Mechanical Engineering and Materials Science, Duke University, Durham, North Carolina 27708, United States; § Renewable Resources and Enabling Sciences Center, National Renewable Energy Laboratory, Golden, Colorado 80401, United States; ∥ Department of Chemistry, Duke University, Durham, North Carolina 27708, United States; ⊥ Materials, Chemical, and Computational Science Directorate, National Renewable Energy Laboratory, Golden, Colorado 80401, United States

## Abstract

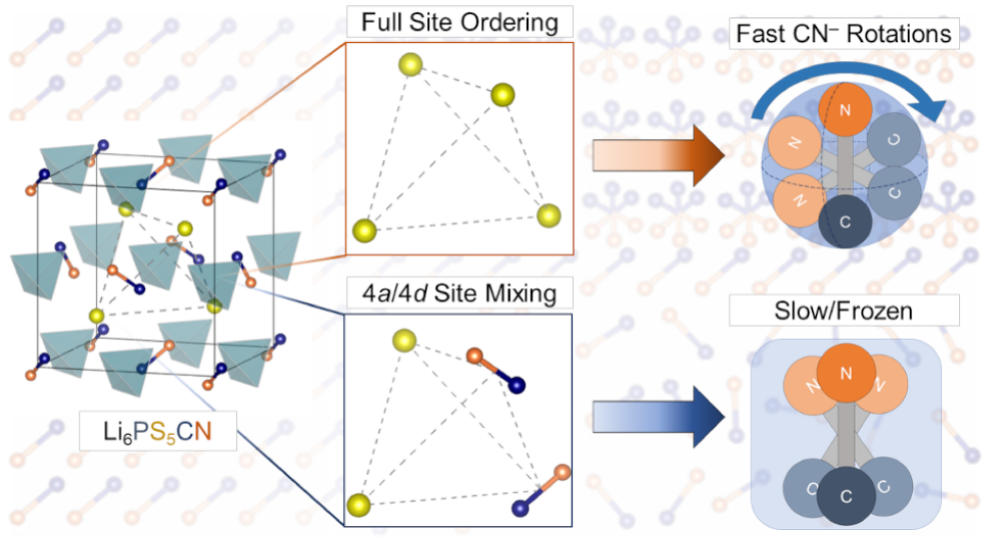

Halide argyrodite solid-state electrolytes of the general formula Li_6_PS_5_*X* exhibit complex static and dynamic disorder that plays a crucial role in ion transport processes. Here, we unravel the rich interplay between site disorder and dynamics in the plastic crystal argyrodite Li_6_PS_5_CN and the impact on ion diffusion processes through a suite of experimental and computational methodologies, including temperature-dependent synchrotron powder X-ray diffraction, AC electrochemical impedance spectroscopy, ^7^Li solid-state NMR, and machine learning-assisted molecular dynamics simulations. Sulfide and (pseudo)halide site disorder between the two anion sublattices unilaterally improves long-range lithium diffusion irrespective of the (pseudo)halide identity, which demonstrates the importance of site disorder in dictating bulk ionic conductivity in the argyrodite family. Furthermore, we find that anion site disorder modulates the presence and time scales of cyanide rotational dynamics. Ordered configurations of anions enable fast, quasi-free rotations of cyanides that occur on time scales of 10^11^ Hz at *T* = 300 K. In contrast, we find that cyanide dynamics are slow or frozen in Li_6_PS_5_CN when site disorder between the cyanide and sulfide sublattices is present at *T* = 300 K. We rationalize the observed differences in cyanide dynamics in the context of elastic dipole interactions between neighboring cyanide anions and local strain induced by the configurations of site disorder that may impact the energetic landscape for cyanide rotational dynamics. Through this study, we find that anion disorder plays a decisive role in dictating the extent and time scales of both lithium ion and cyanide dynamics in Li_6_PS_5_CN.

## Introduction

A new generation of electrochemical energy storage materials are needed to support the transition to a renewable-based power grid and electric transportation. Lithium-ion all-solid-state batteries (ASSBs) have demonstrated the potential to meet many of the challenges associated with current battery technologies. Compared to traditional liquid electrolyte lithium ion batteries, ASSBs can afford improvements to energy density, safety, and thermal and mechanical stability.^1,2^ However, many classes of solid electrolyte materials exhibit lower ionic conductivities compared to commercially available liquid electrolytes.^2^ Understanding the complex, underlying mechanisms of ion transport in the solid state is therefore paramount to designing the next generation of solid-state electrolytes.

Halide argyrodites of the general formula Li_6_PS_5_*X* (X = Cl^–^, Br^–^, I^–^, CN^–^) are promising solid state electrolytes for applications in all-solid-state batteries. The structure is characterized by a framework of isolated PS_4_^3–^ tetrahedra. Halide anions and excess “free” sulfide anions form a pseudodiamond lattice in the cubic unit cell, and lithium ions are highly disordered in the remaining interstitials. The open framework of the argyrodite structure produces a large number of low-energy pathways that enable fast, three-dimensional ion diffusion with demonstrated ionic conductivities up to 10^–2^–10^–3^ S cm^–1^ approaching those of liquid electrolytes.^3−7^

Site disorder plays a decisive role in the long-range ion hopping processes that are crucial for fast, bulk ionic conductivity in the halide argyrodite family. In the argyrodite structure, the anion sublattice can be described by two interpenetrating face-centered cubic lattices offset by a translation vector of (, , ). Site mixing of halide and sulfide anions between these sublattices directly impacts long-range diffusion. Li_6_PS_5_Cl and Li_6_PS_5_Br both exhibit substantial *X*^–^/S^2–^ site mixing between the two sublattices that leads to high ionic conductivities ≥ 10^–3^ S cm^–1^.^4−6,8,9^ In contrast, the large size of the I^–^ anion in Li_6_PS_5_I does not permit site mixing and subsequently leads to ionic conductivities that are several orders of magnitude lower than the chloride and bromide analogs.^3,4,7,10^*Ab initio* molecular dynamics simulations have demonstrated that anion site mixing facilitates long-range ion hopping that gives rise to bulk ionic conductivity, while anion ordering localizes mobile ions and limits long-range diffusion.^11−14^ This behavior is proposed to arise due to the accessibility of short- vs long-range ion hopping motions that are modulated by the differences in potential energy landscape surrounding the mobile ions.

Beyond the static disorder of site mixing, dynamic disorder directly impacts ionic conductivity across a variety of crystal structures. Soft and polarizable host lattices with lower-energy phonon modes are correlated with lower activation barriers to ionic conductivity.^2,15−20^ These soft vibrational modes help carry the mobile ion over the saddle point between neighboring sites.^5^ Local motions of polyatomic moieties have long been proposed as a mechanism to promote ion conduction by coupling dynamics of these species with mobile ion hopping.^21^ Incorporation of molecular species such as pseudohalides (CN^–^, BH_4_^–^) into the halide argyrodite structure have been shown to lower activation barriers for ionic conductivity,^22−24^ though identifying the presence and impact of coupled dynamics presents an ongoing challenge. Through solid-state nuclear magnetic resonance studies, Hanghofer et al. and Smith et al. demonstrated that rotations of PS_4_^3–^ tetrahedra in the halide argyrodite family occur on similar time scales to lithium hopping and have been correlated with increases in long-range ion transport.^25,26^ Interestingly, Brinek et al. recently revealed that structural disorder directly impacts the time scales of PS_4_^3–^ rotational dynamics in Li_6_PS_5_I,^27^ which suggests that thesedynamics are intimately linked with disorder-induced changes to the local potential energy landscape. The complexity of these coupled motions has so far precluded a cohesive understanding of if or how these coupled motions occur and how to control these interactions in solid-state electrolytes.

In this work, we unravel the rich connections between anion site disorder, lithium ion and cyanide dynamics, and Li-CN coupling in the cyanide argyrodite Li_6_PS_5_CN. Temperature-dependent synchrotron powder X-ray diffraction measurements coupled with differential scanning calorimetry indicate that Li_6_PS_5_CN adopts the cubic argyrodite structure between *T* = 90–300 K. We do not observe any phase transitions or thermodynamic events that would indicate collective changes in cyanide dynamics or the onset of cyanide ordering, which suggests that Li_6_PS_5_CN adopts a dipole glass state in which cyanide anions are static and orientationally disordered. Rietveld refinement indicates that Li_6_PS_5_CN exhibits substantial anion site disorder between S^2–^ and CN^–^. ^7^Li solid-state nuclear magnetic resonance spectroscopy and electrochemical impedance spectroscopy of Li_6_PS_5_CN reveals fast local and long-range lithium ion diffusion processes. Experimental measurements of Li^+^ transport are supported by machine learning-assisted molecular dynamics (MD) simulations; from simulations of lithium ion motion, we find that site mixing between sulfide and (pseudo)halide facilitates long-range lithium ion diffusion processes across all members of the Li_6_PS_5_*X* (*X =* Cl^–^, Br^–^, CN^–^, I^–^) family. MD simulations of Li_6_PS_5_CN further reveal that the configuration of S^2–^/CN^–^ site disorder dictates the extent and time scales of cyanide dynamics. Anion-ordered structures produce fast, quasi-free rotation of the cyanide ions at *T* = 300 K. In contrast, 50% site mixing between the CN^–^ and S^2–^ sublattices in Li_6_PS_5_CN result in slow or frozen cyanide dynamics (*T* = 300 K), which we attribute to elastic dipole coupling interactions between neighboring cyanides or local strain brought about by different configurations of site disorder. Through these MD simulations, we further find that lithium ions are electrostatically coupled to the cyanide ions and preferentially reside at the negatively charged carbon end of the cyanide anion regardless of the time scales of cyanide dynamics. Taken together, this study demonstrates the complex interplay between site disorder, lithium ion diffusion, and rotational dynamics of cyanide anions in the pseudohalide argyrodite Li_6_PS_5_CN.

## Results

### Structural Characterization of Li_6_PS_5_CN

**Figure 1:**
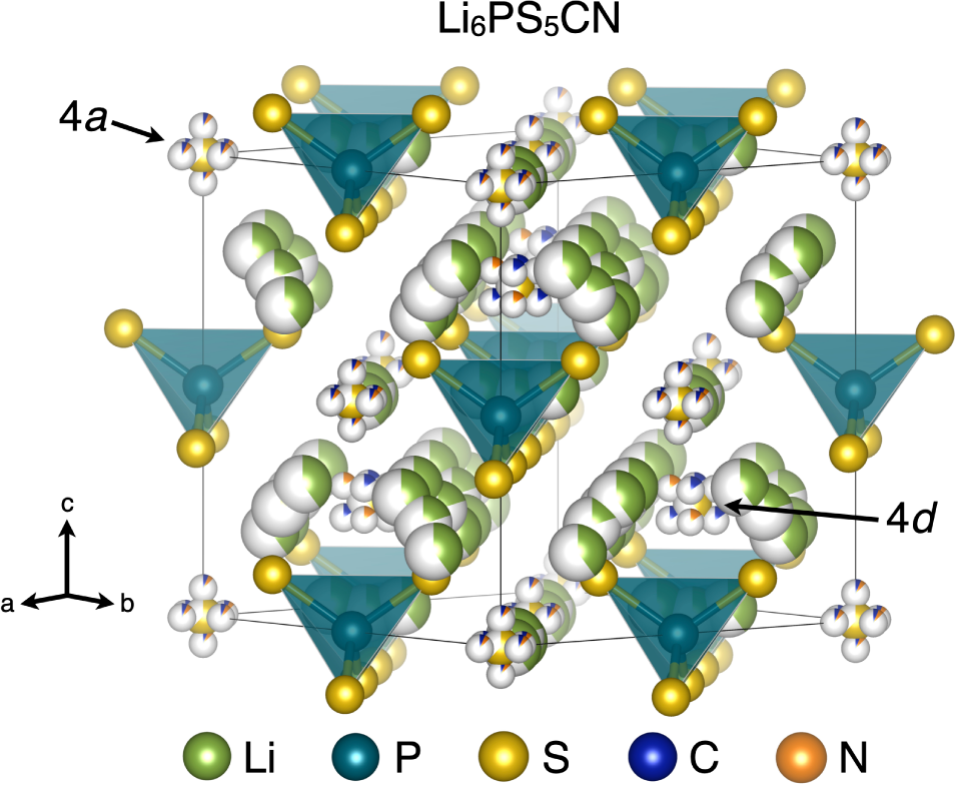
Structure of Li_6_PS_5_CN, *F*4̅3*m*. Partial occupancies (shown as partially shaded spheres) of S, C, and N were determined through Rietveld refinement at *T* = 300 K. The Li^+^ positions and occupancies were fixed to previously reported values.

Li_6_PS_5_CN adopts the cubic argyrodite structure (space group *F*4̅3*m*) at room temperature (Figure 1).^23^ The structure is characterized by a framework of isolated PS_4_ tetrahedra. Cyanide ions and excess “free” sulfide ions occupy the 4*a* (0, 0, 0) and 4*d* (0.75, 0.75, 0.75) Wyckoff sites to form a pseudodiamond lattice arrangement of anions with substantial site mixing of CN^–^/S^2–^ between these sites.^28^ The lithium ions reside in the interstices and are highly disordered across the 48*h* and 24*g* sites.

**Figure 2:**
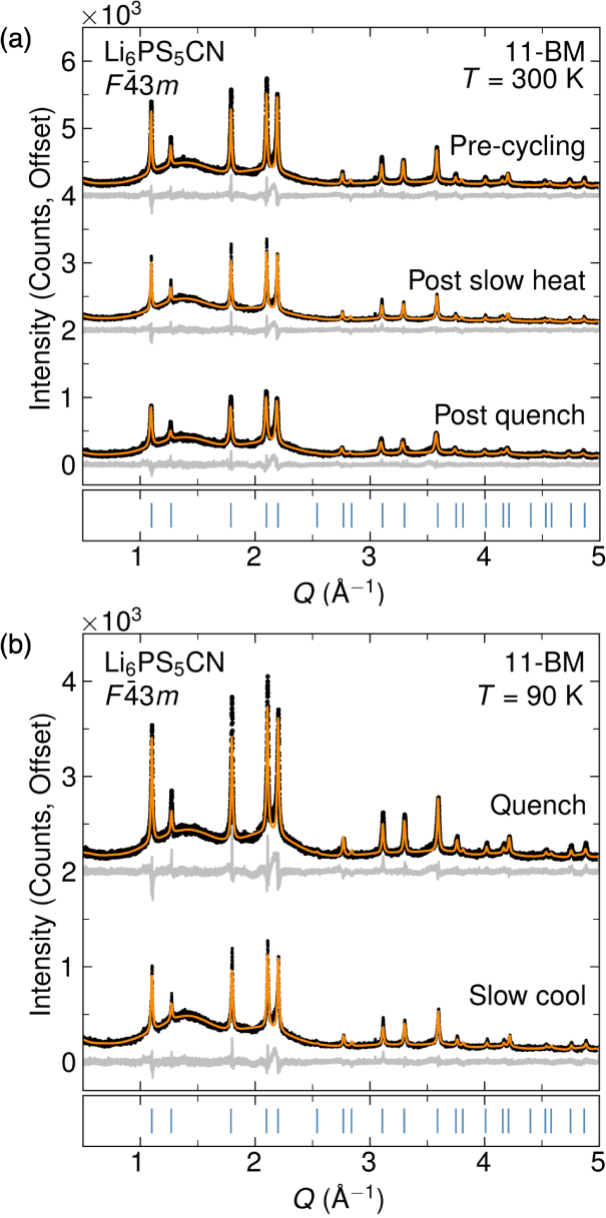
Exemplary Rietveld refinements of Li_6_PS_5_CN from the 11-BM-B beamline. (a) Rietveld refinements at *T* = 300 K of the sample initially before any temperature changes, after heating from the slow cooled temperature ramp, and after heating from quenching to *T =* 90 K. (b) Rietveld refinements at *T* = 90 K of Li_6_PS_5_CN after slow cooling and quenching.

The structure of Li_6_PS_5_CN and the extent of CN^–^/S^2–^ site disorder was determined through Rietveld refinement of high-resolution synchrotron powder X-ray diffraction (SXRD) collected at the 11-BM-B beamline (Advanced Photon Source, Argonne National Laboratory), as shown in Figure 2. In order to account for the geometry and orientational disorder of the cyanide ion and to accommodate site mixing between the CN^–^/S^2–^ ions within the structure, we constructed a constrained model of the cyanide argyrodite for analysis of the SXRD data. Cyanide ions were introduced at both the 4*a* and 4*d* Wyckoff sites of the argyrodite structure. The CN^–^ ions at the 4*a* sites (C1, N1) were aligned along the lattice vectors. The center of the CN bond was positioned at the (0, 0, 0) site and the C1 and N1 positions were offset by ±0.58 Å along the [100] direction to account for the reported CN bond length of ∼1.16 Å.^29^ At this general position (24*f*), the 4-fold rotoinversion symmetry operation along the principal symmetry axis generates six C1 and N1 atoms at each corner and face of the unit cell. At the 4*d* Wyckoff site, the center of the CN bond was positioned at (0.75, 0.75, 0.75) and the cyanide ions (C2, N2) were offset along the [111] direction by one-half the CN bond length to align the cyanides along the body diagonal of the cubic unit cell. The positions of the C and N atoms at both the 4*a* and 4*d* sites were chosen to align with high-symmetry directions in the cubic unit cell in order to promote crystallographic simplicity of the heavily disordered structural model. To accommodate the geometry of the cyanide ion, the positions of the C and N atoms at both 4*a* and 4*d* sites were constrained as fractions of the lattice vector to preserve the length of the CN bond during Rietveld refinement. Additionally, we constrained the occupancies of the CN^–^ and free S^2–^ ions residing at both 4*a* and 4*d* sites to preserve the stoichiometry of Li_6_PS_5_CN based upon the multiplicity of the general positions. As X-rays are insensitive to the position of lithium within the structure, we constrained the Li^+^ occupancies at the 48*h* and 24*g* sites in accordance with prior reports of Li_6_PS_5_Br.^30^ We note that the actual distribution of lithium ions may differ in Li_6_PS_5_CN due to the difference in anion charge density and nonspherical shape of the cyanide anion; however, we lack the ability to resolve the average positions and occupancies of lithium ions with X-ray scattering. The mathematical relationships between positions and occupancies used in all Rietveld refinements can be found in the TOPAS input file included in the Supporting Information.

Rietveld refinement of this constrained structural model with SXRD data collected at *T* = 300 K is shown in Figure 2 and is consistent with Li_6_PS_5_CN adopting the cubic argyrodite structure with orientationally disordered cyanide ions and site disorder between CN^–^/S^2–^ at the 4*a*/4*d* sites. The structural parameters extracted from the Rietveld refinement at *T* = 300 K (before temperature cycling) are reported in Table 1 and are similar to those reported previously.^23^ At *T* = 300 K, we find that the occupancy of the 4*a* Wyckoff site is 67(9)% S^2–^/32(1)% CN^–^ ions, while the occupancy of the 4*d* site is 32(1)% S^2–^/67(9)% CN^–^ ions.

**Table 1:** Structural Parameters Extracted from Rietveld Refinements of the Cubic Argyrodite Structure of Li_6_PS_5_CN (Space Group *F*4̅3*m*) at *T* = 300 K from High-Resolution Synchrotron Powder X-ray Diffraction before Temperature Cycling^a^

atom	Wyckoff site	*x*	*y*	*z*	*U*_iso_ (Å^2^)	occ.
Li1	48*h*	0.3044	0.0253	0.6956	0.0253	0.4
Li2	24*g*	0.25	0.009	0.75	0.0253	0.2
P	4*b*	1.0	0.5	1.0	0.0435(2)	1.0
S1	16*e*	0.122(4)	–0.122(4)	0.622(4)	0.068(1)	1.0
S2	4*a*	0	0	0	0.0253	0.67(9)
S3	4*d*	0.75	0.75	0.25	0.0253	0.32(1)
C1	24*f*	0.058(4)	0	0	0.0499	0.05(3)
N1	24*f*	0.058(4)	0	0	0.0499	0.05(3)
C2	16*e*	0.716(3)	0.716(3)	0.716(3)	0.0499	0.17(0)
N2	16*e*	0.783(7)	0.783(7)	0.783(7)	0.0499	0.17(0)

a Lattice parameter = 9.9240(1) Å, *R*_wp_ = 7.44%.

**Table 2:** Structural Parameters Extracted from Rietveld Refinements of the Cubic Argyrodite Structure of Li_6_PS_5_CN (Space Group *F*4̅3*m*) from High-Resolution Synchrotron Powder X-ray Diffraction Collected at *T* = 90 K after Quenching from *T* = 300 K to *T* = 90 K^a^

atom	Wyckoff site	*x*	*y*	*z*	*U*_iso_ (Å^2^)	occ.
Li1	48*h*	0.3044	0.0253	0.6956	0.0253	0.4
Li2	24*g*	0.25	0.009	0.75	0.0253	0.2
P	4*b*	1.0	0.5	1.0	0.048(1)	1.0
S1	16*e*	0.122(6)	–0.122(6)	0.622(6)	0.0635(8)	1.0
S2	4*a*	0	0	0	0.0253	0.65(1)
S3	4*d*	0.75	0.75	0.25	0.0253	0.35(1)
C1	24*f*	0.059(1)	0	0	0.0499	0.058(1)
N1	24*f*	0.059(1)	0	0	0.0499	0.058(1)
C2	16*e*	0.716(1)	0.716(1)	0.716(1)	0.0499	0.163(1)
N2	16*e*	0.784(1)	0.784(1)	0.784(1)	0.0499	0.163(1)

a Lattice parameter = 9.8878(1) Å, *R*_wp_ = 8.33%.

**Figure 3:**
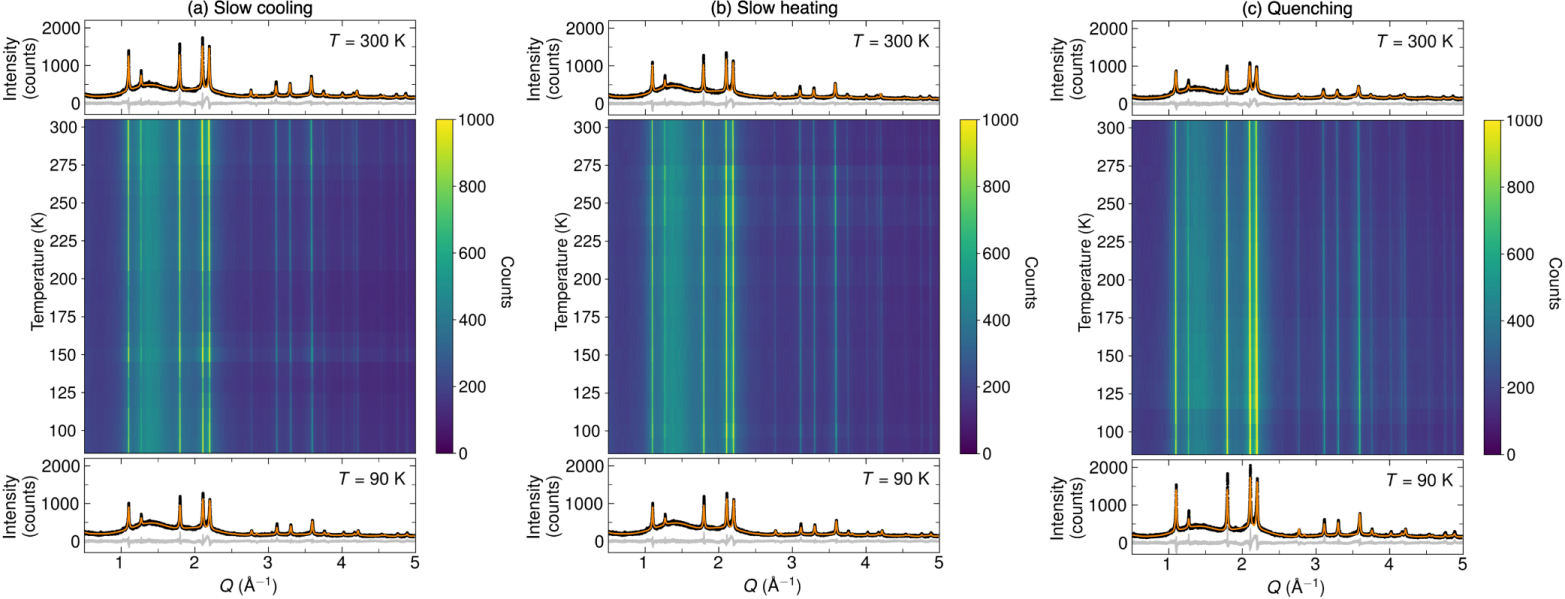
Color plot representations of the diffraction patterns for Li_6_PS_5_CN during (a) slow cooling, (b) heating from slow cooling, and (c) heating from quenched cooling. Rietveld refinements are included for the SXRD patterns taken at *T* = 300 K and *T* = 90 K before and after temperature cycling.

Plastic crystals, in which orientational degrees of freedom exist within an otherwise long-range periodic structure, are well-known to undergo phase transitions driven by collective ordering of the molecular species.^31−39^ These transitions are often hysteretic and are dependent upon thermal history of the sample.^39−43^ In order to identify phase transitions associated with collective ordering of the cyanide ions in Li_6_PS_5_CN, we collected temperature-dependent SXRD data at 11-BM-B. SXRD patterns were collected first on cooling from *T* = 300 K to *T* = 90 K in 10 K decrements and then again on warming. We also performed quenching studies to *T* = 90 K followed by slow warming to *T* = 300 K using a pristine sample of Li_6_PS_5_CN to eliminate the potential impact of thermal cycling. We find that the quenched sample exhibits nearly the same degree of S^2–^/CN^–^ site mixing as the sample used for slow temperature ramping; the occupancy of the 4a Wyckoff site is 65(1)% S^2–^/35(1)% CN^–^ ions, while the occupancy of the 4d site is 35(1)% S2–/67(5)% CN^–^ ions. Structural parameters from the quenched sample at T = 90 K are provided in Table 2. Representative Rietveld refinements for data collected at *T* = 300 K and *T* = 90 K are shown in Figure 2. As shown in Figures 2 and 3, Li_6_PS_5_CN retains the cubic argyrodite structure to *T* = 90 K in both slow-cooled and quenched samples. This observation is further supported by the absence of peaks in the differential scanning calorimetry data (Figure S1), which also indicates that the cyanide ions are not undergoing any crystallographically unresolvable transitions due to changes in cyanide dynamics or ordering over this temperature range.^44^ Interestingly, we note that temperature cycling of Li_6_PS_5_CN appears to influence the breadth and intensity of the Bragg reflections (Figures 2 and 3), which may be suggestive of ferroelastic behavior of Li_6_PS_5_CN. Similar behavior is frequently observed in materials with orientationally disordered molecular species, including alkali cyanides,^35,36,40^ and hybrid organic–inorganic perovskites.^41,42,45^ These data suggest that the cyanides are orientationally disordered and there is no collective ordering or freezing transitions of the cyanide ions in the structure during either slow cooling or quenching.^35,36^ We also note the presence of diffuse scattering in the SXRD patterns that manifests as a broad hump between *Q =* 1 – 2 Å^–1^; diffuse scattering is not uncommon in orientationally disordered materials, and we suspect that the incoherent scattering may be reflective of the large degree of both orientational and site disorder in the structure.

**Figure 4:**
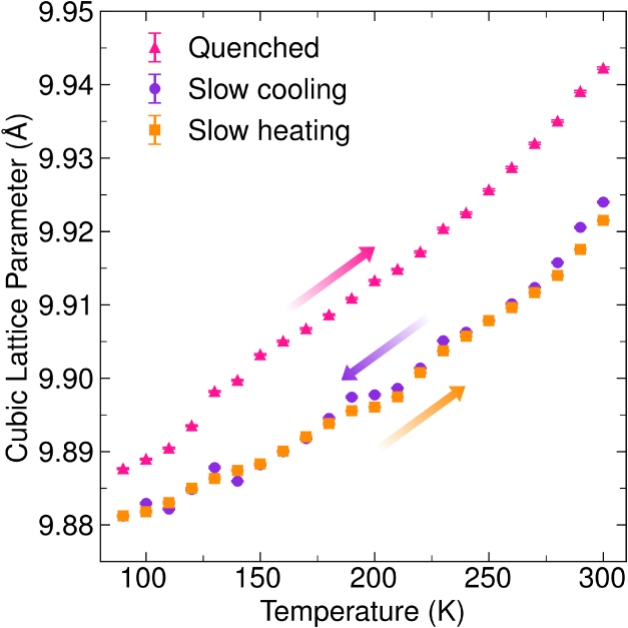
Cubic lattice parameter for the Li_6_PS_5_CN as a function of temperature for each of the heating and cooling profiles. Data collected on slow cooling and then slow heating are shown as purple circles and orange squares, respectively. Data collected on slow heating from quenching are shown as pink triangles. The arrows indicate the direction of heating/cooling.

We further analyzed temperature-dependent SXRD data collected during slow temperature ramping and quenching to understand how the structure of Li_6_PS_5_CN evolves during temperature cycling. Using the constrained models refined from the *T* = 300 K SXRD data (Table 1) and *T* = 90 K quenched sample, we performed sequential Rietveld refinements as a function of temperature for SXRD data collected during temperature cycling. The occupancies of the S^2–^ and CN^–^ at the 4*a* and 4*d* Wyckoff sites were fixed to the starting structures and not permitted to refine. Although no long-range cyanide ordering is observed, the unit cell did demonstrate an increase in lattice parameter as a function of increasing temperature as shown in Figure 4. Interestingly, the quenched sample exhibited a larger lattice parameter at all temperatures compared to the slow cooled/heated sample (9.9422(2) Å compared to 9.9240(1) Å, respectively). The difference between slow cooled and quenched samples only constitutes approximately 1% of the overall lattice parameter, which we attribute to differences in preparation between samples for slow-ramping and quenched trials. The measured lattice parameters of halide argyrodites are highly sensitive to the preparation method, particularly for solution-phase synthesis approaches; it is therefore not surprising to experimentally observe subtle differences between multiple samples of the same nominal composition.^46,47^

### Electrochemical Impedance Spectroscopy

**Figure 5:**
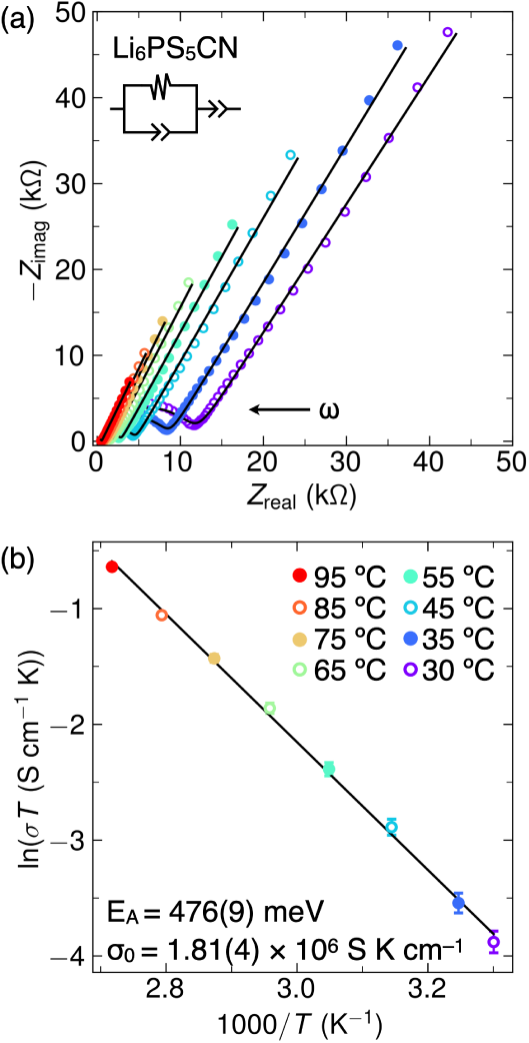
(a) Representative Nyquist plots of temperature-dependent electrochemical impedance spectroscopy measurements of Li_6_PS_5_CN. Data are shown as circles and fits to the (*R*_1_*Q*_1_) + *Q*_2_ equivalent circuit model are shown as black lines. Data points collected at higher frequencies are found toward lower *Z*_real_. At *T* = 30 °C, the apex frequency of the *R*_1_*Q*_1_ element occurs at approximately 1.89 × 10^6^ Hz. (b) Representative Arrhenius plot obtained from temperature-dependent electrochemical impedance spectroscopy data shown in (a).

We performed temperature-dependent AC electrochemical impedance spectroscopy (EIS) measurements to provide complementary insight into the bulk Li^+^ transport in Li_6_PS_5_CN. Representative Nyquist plots of the real and imaginary contributions to the impedance are shown in Figure 5a. In order to quantify the evolution of electrochemical processes as a function of temperature, we modeled the real and imaginary components of the impedance with a (*R*_1_*Q*_1_) + *Q*_2_ equivalent circuit, where *R* is a resistor and *Q* is a constant-phase element. We assign the high-frequency semicircle (*R*_1_*Q*_1_) to bulk Li^+^ transport and the low-frequency tail to capacitive processes (*Q*_2_) at the interfaces with the blocking gold electrodes. At *T* = 30 °C, we find a bulk ionic conductivity of σ_1_ = 6.8(2) × 10^–5^ S cm^–1^; this value is closely aligned with the room temperature ionic conductivity of σ_RT_ = 6(2) × 10^–5^ S cm^–1^ determined from our previous study.^23^ Our assignment of the *R*_1_*Q*_1_ element to bulk ion transport is supported by the capacitance value of 7.22 pF at *T* = 30 °C.^48^ Using this model, we find that the temperature-dependent Li^+^ conductivity of Li_6_PS_5_CN is well-captured by the Arrhenius relationship ln(σ_1_*T*) = σ_0_ exp(−*E*_A_/*k*_B_*T*) at *T* = 30–95 °C. As illustrated in Figure 5b, this yields an activation barrier for bulk Li^+^ transport of *E*_A_ = 476(9) meV and an Arrhenius prefactor of σ_0_ = 1.81(4) × 10^6^ S K cm^–1^. Temperature-dependent EIS fitting results are tabulated in Table S1.

### ^7^Li Solid-State Nuclear Magnetic Resonance Spectroscopy

**Figure 6:**
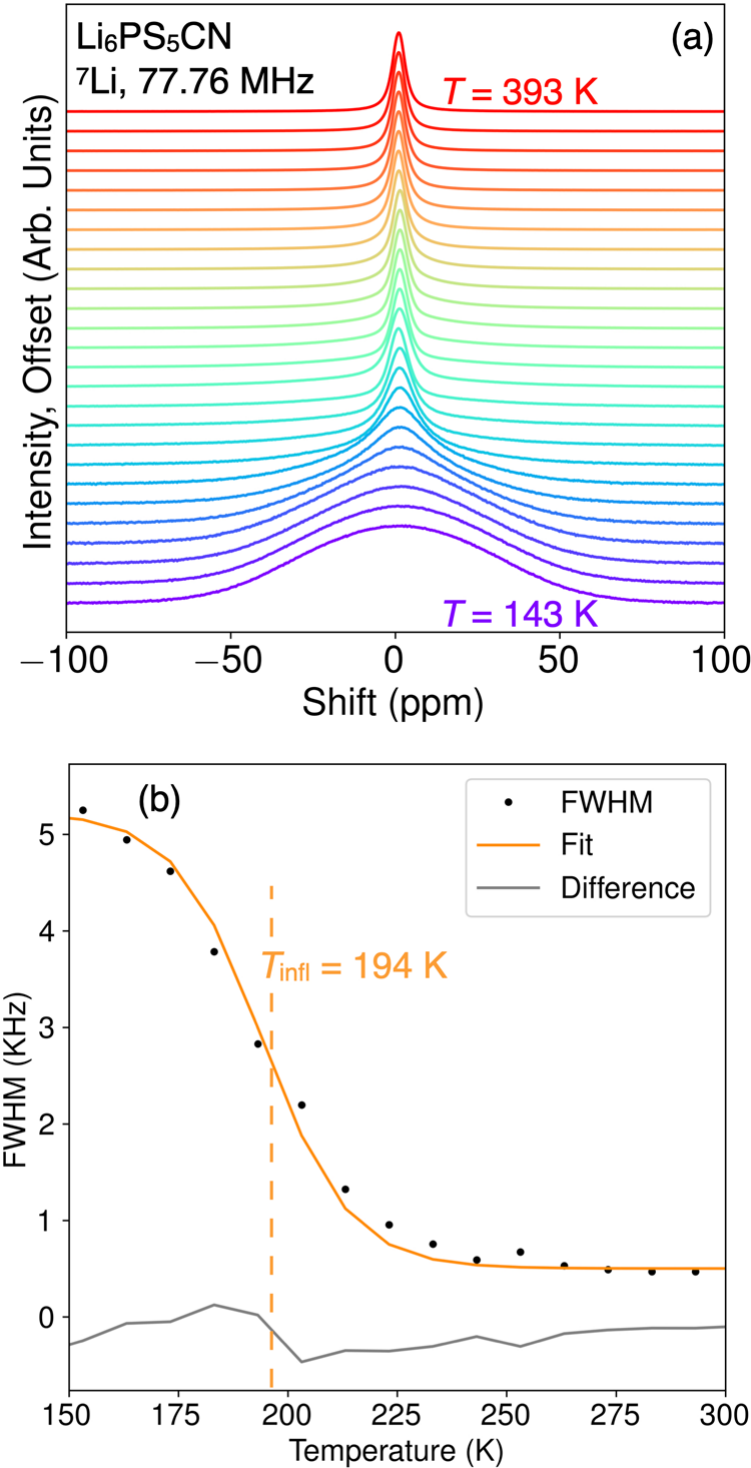
^7^Li SSNMR fwhm analysis of the Li_6_PS_5_CN argyrodite. (a) shows a waterfall plot demonstrating the motional narrowing of the static ^7^Li peak as a function of temperature. (b) shows the fwhm of the peaks from (a). The solid orange line represents a sigmoidal regression.

We employed ^7^Li solid-state nuclear magnetic resonance spectroscopy (SSNMR) to probe the local lithium ion dynamics in Li_6_PS_5_CN. The onset of bulk Li^+^ motion in Li_6_PS_5_CN was first characterized through static line width analysis. We observe motional narrowing of the line width with an increase in temperature, as shown in Figure 6, wherein the lithium ions undergo a transition to liquid-like dynamic motion at higher temperatures. The peak shapes were deconvoluted into Gaussian and Lorentzian components using a pseudo-Voigt model, as shown in Figure S2. The peak profile transitioned from Gaussian to Lorentzian with increasing temperature, as is commonly observed in superionic conductors as the fraction of mobile Li^+^ ions increases.^3,49,50^ At lower temperatures, neighboring lithium ions are surmised to maintain strong dipole–dipole interactions with one another, resulting in a range of distinct shielding environments and chemical shifts, which produces a broad Gaussian peak. As temperature increases and lithium ions gain enough energy to undergo fast local hopping, the dipole–dipole interactions are continuously averaged and the associated NMR peak narrows into a Lorentzian distribution. The onset temperature of bulk Li^+^ movement in Li_6_PS_5_CN was determined by extracting the fwhm as a function of temperature. We fit a sigmoidal regression to the fwhm v. temperature data, which gives an inflection temperature of *T*_infl_ = 194 K. From visual inspection, we approximate the onset temperature of motional narrowing to be *T*_onset_ ≈ 175 K. Using the empirical Waugh–Fedin expression *E*_A_(eV) = 1.67 × 10^–3^·*T*_onset_,^51^ we roughly estimate an activation energy of ∼0.282 eV for Li^+^ movement in the cyanide argyrodite. For comparison, activation barriers for halide argyrodites determined by motional narrowing typically range between 200 and 300 meV,^46,49^ which indicates that Li_6_PS_5_CN exhibits similar lithium ion diffusion behavior to other members of the argyrodite family.

**Figure 7:**
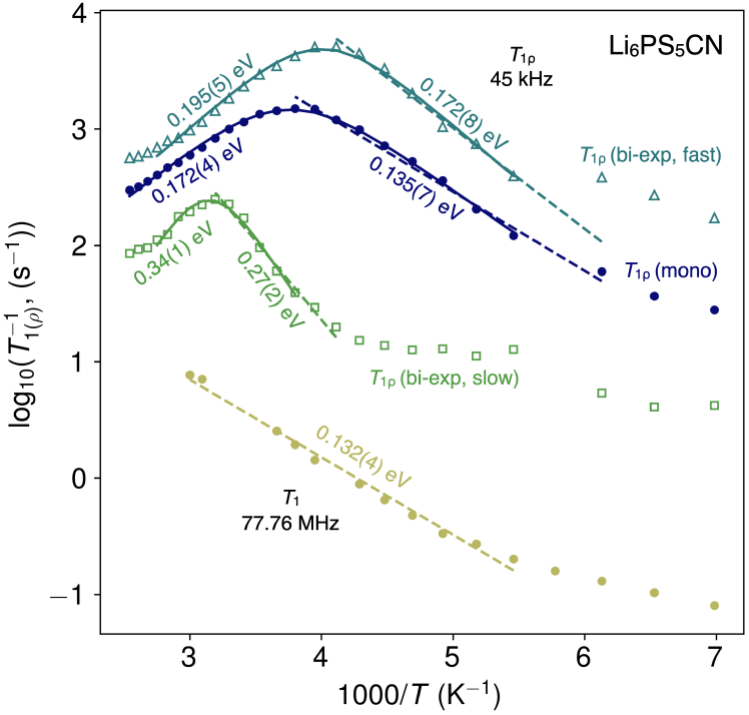
^7^Li spin–lattice relaxation data for Li_6_PS_5_CN. Relaxation rates were collected in the laboratory frame (1/*T*_1_) at a Larmor frequency of 77.76 MHz (yellow circles). Relaxation rates in the rotating frame (1/*T*_1ρ_) were collected at a spin-lock frequency of 45 kHz. The blue circles are calculated from a monoexponential decay fitted according to BPP theory. The (1/*T*_1ρ_) data were also fit to a biexponential decay (light blue triangles and green squares) which results in a fast motional regime (triangles) and a slow motional regime (squares). The solid lines represent a BPP fit according to eq 2, while dashed lines indicate a linear fit to Arrhenius behavior. The activation energies for the high temperature flanks were calculated through the BPP fit, and activation energies for the low temperature flanks were calculated through the Arrhenius fit.

^7^Li NMR spin–lattice relaxation (SLR) provides additional insight into distinct time- and length-scales of ion diffusion in Li_6_PS_5_CN. ^7^Li SLR NMR relaxometry data collected in the laboratory reference frame (*R*_1_ ≡ 1/*T*_1_) and rotating frame (*R*_1ρ_ ≡ 1/*T*_1ρ_) are shown in Figure 7. Relaxation rates in the laboratory reference and rotating reference frame are recorded at a Larmor frequency of ω_0_/2π = 77.76 MHz and spin-lock frequency of ω_1_/2π = 45 kHz, respectively. 1/*T*_1_ corresponds to fast local hopping, while 1/*T*_1ρ_ corresponds to slower, longer range hopping processes. The motional correlation rates(τ_*c*_) for lithium ions for both frequency regimes are equal to the resonance frequency at the temperature (*T*_max_) for which *R*_1_ (*R*_1ρ_) reach a maximum (i.e., ω_*c*_τ_*c*_ ≈ 1 at *T*_max_).^52^

Fast, short-range Li^+^ hopping is captured by relaxation rates recorded in the laboratory reference frame (1/*T*_1_), where Li^+^ jump rates are on the order of the Larmor frequency (77.76 MHz). As shown in the low-temperature 1/*T*_1_ data (*T* < 200 K) in Figure 7, we observe nondiffusive background relaxation processes that have previously been attributed to relaxation processes due to lattice vibrations or coupling of Li^+^ spins to paramagnetic impurities.^9,51−53^ At *T* ≈ 200 K, we observe a sharp increase in the relaxation rates that are characteristic of Li^+^ diffusive motion. We note that our measurements do not reach high enough temperatures to observe a maximum in the 1/*T*_1_ relaxation rates, as is typically observed in other fast ion conductors.^52^ Li_6_PS_5_CN decomposes near *T* = 425 K,^23^ which limits the maximum temperature range for SLR NMR measurements. We fit the low-temperature diffusive flank of the log(1/*T*_1_) vs 1000/*T* with an Arrhenius expression (excluding the low-temperature region in which nondiffusive relaxation processes dominate), which yields an activation barrier of *E*_A,low_ = 0.132(4) eV for fast, short-range Li^+^ hopping in Li_6_PS_5_CN. This activation barrier is comparable to those reported for other halide argyrodites which typically range from 90 to 150 meV and are consistent with rapid local hopping.^9,27,49,54^

Relaxation rates recorded in the rotating frame (1/*T*_1ρ_) provide insight into longer-range lithium ion diffusion occurring at slower rates in the kHz regime.^54^ The signal decay was measured over time, and the time-dependent decay was fit to an exponential decay function. The fitting function can comprise a single exponent (monoexponential) or two exponent terms (biexponential). We find that the relaxation processes for Li_6_PS_5_CN are best described by a biexponential decay; the presence of multiple relaxation processes is consistent with a disordered, inhomogeneous structure with a distribution of defect sites that produces two distinct *T*_1ρ_^–1^ features.^55^ Representative examples of a monoexponential and biexponential fit at *T* = 303 K are shown in Figure S3 to demonstrate the differences in fit between the two models. In order to compare Li_6_PS_5_CN to prior literature of other halide argyrodites, we have also included the relaxation rates determined from fitting the relaxation data to a monoexponential decay, which represents a weighted average of the two biexponential coefficients and is still representative of the long-range behavior. The results are shown in Figure 7. The behavior we observe in relaxometry is consistent with relaxation processes associated with lithium ion diffusion.^9,25,49,54^

The resulting relaxation rates from both the monoexponential and biexponential decay were fit to the model derived from the theory by Bloembergen, Purcell, and Pound (BPP).^56^ In BPP theory, the lithium jump rate, 1/τ, correlates to temperature through an Arrhenius relationship, described by the following equation:
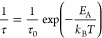
1where (*τ*_0_^–1^) is the jump attempt frequency. BPP theory relates the spectral density function, *J*(ω_1_), to the jump rate through the following relationship:
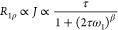
2where  is proportional to *T*_1ρ_^–1^ (also referred to as *R*_1ρ_). The data can be fit according to BPP theory to provide activation energy, β, and jump rates, where β is assumed to be 2 for uncorrelated ion movement.^50,57^ Asymmetric slopes on either flank of the maxima indicate deviation from β = 2. In Li_6_PS_5_CN we find β = 1.91, potentially indicating the presence of correlation effects (e.g., Coulombic interactions) which are common in disordered materials where there are multiple nearly degenerate diffusion pathways.^50^ The fitted curves according to the BPP model are also shown in Figure 7 and fitted parameters are tabulated in Table 3. Additionally, the low-temperature flank of each data set was fit with a linear Arrhenius model to yield the low-temperature activation energy, *E*_A__,__low_.

**Table 3:** Results of the Overall SSNMR *T*_1ρ_^–1^ Fit including the Monoexponential (Mono) and Biexponential (Fast, Slow) Decay fits as Shown in Figure 7^a^

fit	*E*_A__,__BPP_ (eV)	*E*_A__,__low_ (eV)	*T*_max_ (K)	*C* (s^–2^)	β	τ_0_ (s)
*T*_1ρ_^–1^(mono)	0.172(4)	0.135(7)	263.15	1.6(3) × 10^8^	1.91(4)	1.0(2) × 10^–9^
*T*_1ρ_^–1^(biexp, fast)	0.195(5)	0.172(8)	243.15	5.5(2) × 10^9^	1.97(4)	2.1(5) × 10^–10^
*T*_1ρ_^–1^(biexp, slow)	0.34(1)	0.27(2)	313.15	2.8(1) × 10^8^	2.001(0)	8(4) × 10^–12^

a *E*_A,BPP_ is the high temperature flank activation energy extracted from the BPP fit, while *E*_A,low_ is the low temperature flank activation energy extracted through a linear regression. β, τ_0_, and the prefactor term C were all obtained through the BPP fit regression, fit to eq 2

As shown in Figure 7 and summarized in Table 3, the monoexponential fit reveals a low-temperature activation energy of Li^+^ motion of *E*_A,low_ = 0.135(7) eV, and a high-temperature activation energy of *E*_A,high_ = 0.172(4) eV. These values are on the same order of magnitude, but slightly lower than those reported for other members of the halide argyrodite family.^9,49,54,58^ This observation is consistent with AC electrochemical impedance spectroscopy measurements in our previous study, wherein bulk conductivity indicated a similar yet lower activation energy of Li^+^ motion for Li_6_PS_5_CN compared to Li_6_PS_5_Br.^23^ The biexponential fits provide two *T*_1ρ_ curves that represent fast and slow motional regimes (triangles and squares in Figure 7) occurring on kHz time scales. Biexponential *T*_1ρ_ behavior was previously observed in the Li_6+*x*_P_1–*x*_Ge_*x*_S_5_I argyrodites which was attributed to different time scales between higher-energy intercage and lower-energy intacage ion dynamics.^54^ The faster ion behavior dominates at higher temperatures, and the relaxation processes converge to a monoexponential decay (Figure S4). This result is also observed in Li_2_OHCl, which exhibits biexponential decay behavior in the *T*_1_ regime.^55^

Relaxometry can also provide insight into the time scales of lithium ion diffusion. At the 1/*T*_1ρ_ maximum, the lithium ion jump rate, 1/τ, is on the order of the spin-lock frequency ω_1_/2π = 45 kHz. We find that the monoexponential *T*_1ρ_ reaches a maximum at *T*_max_ = 263.15 K. Using the relationship ω_1_τ = 0.5, we find a jump frequency of 1/τ ≈ 5.6 × 10^5^ s^–1^ at this temperature. The lithium ion diffusion coefficient can be estimated using the Einstein–Smoluchowski equation:^59^
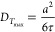
3where *a* is the Li–Li jump distance. Here, we take the approximate distance for intercage jumps between the 48*h*–48*h* Li sites to be ∼3.1 Å.^25^ We note that this jump distance is an approximation; our structural analysis through synchrotron powder diffraction does not provide adequate sensitivity to the lithium sublattice to accurately determine positions and distances in Li_6_PS_5_CN. Using this approximate jump distance yields a diffusion coefficient on the order of *D*_263K_ ≈ 8.97 × 10^–11^ cm^2^ s^–1^. This value is lower than the typical diffusion coefficients on the order of 10^–5^–10^–8^ cm^2^ s^–1^ reported from SLR analysis of argyrodites in the laboratory frame (1/*T*_1_).^49,60^ Since *T*_1ρ_ probes slower motions (kHz compared to MHz), it is expected that the Li^+^ diffusion coefficient from these relaxometry data would be lower compared to those determined from *T*_1_ data. The lithium ion jump rate of 1/τ ≈ 5.6 × 10^5^ s^–1^ for Li_6_PS_5_CN determined from *T*_1ρ_ is closely comparable to those determined for other argyrodites (1/τ ≈ 2.512 × 10^5^ s^–1^ for Li_6_PS_5_Br^46^ and 1/τ ≈ 1.4 × 10^5^ s^–1^ for Li_7_PSe_6_;^49^ these slower rates have been previously attributed to longer range diffusion through grain boundaries^46^ and suggests that long-range diffusion processes in Li_6_PS_5_CN are commensurate with those of other polycrystalline argyrodites.

### Machine Learning-Assisted Molecular Dynamics Simulations

In order to understand the dynamics of cyanide and lithium ions in Li_6_PS_5_CN we employed machine learning (ML)-assisted molecular dynamics (MD) simulations within the framework of density functional theory using the FHI-aims code^61^ and machine learning potentials determined by the MLIP-2 code^62^ in the moment tensor potential (MTP) form.^63^ Further details can be found in the Methods and Materials section. Moreover, the Supporting Information includes scatter plots of the MTP energies and forces compared to the direct density functional theory (DFT) energies, in order to validate our MTP training. These plots are presented in Figures S5–S9. Figures S10–S12 provide the average potential energies and lattice parameters for each Li_6_PS_5_*X* composition and for each disorder configuration over all the simulated trajectories.

**Figure 8:**
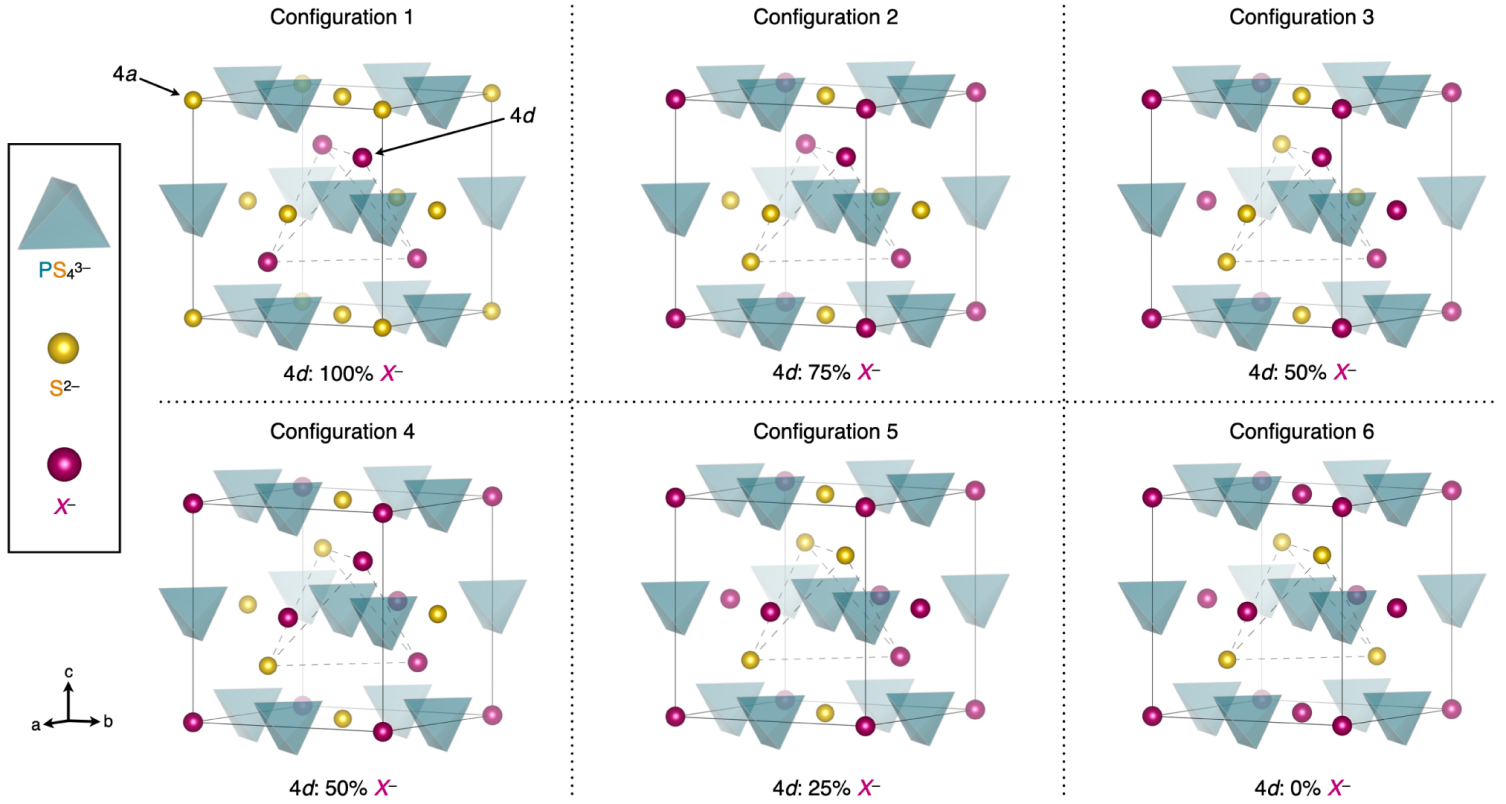
Structural representations of the six disorder configurations used as starting models for molecular dynamics simulations. (Pseudo)halide ions, *X*^–^, are represented by magenta spheres, while sulfur atoms are represented by yellow spheres. The 4*a* and 4*d* Wyckoff sites are labeled in Configuration 1 for clarity, and the 4*d* Wyckoff sites are connected by dashed gray lines as a guide to the eye. The P and S atoms that comprise the PS_4_^3–^ tetrahedra are omitted for clarity, and the tetrahedra are shown as teal polyhedral representations. Lithium ions are also omitted for clarity.

The halide argyrodite structure is well-known to exhibit *X*^–^/S^2–^ site mixing at the 4*a* and 4*d* Wyckoff sites. The extent of site mixing (particularly at the 4*d* site) depends on the identity of the halide and plays a critical role in ion transport processes in the argyrodites.^4,6,11,13^ For example, the lack of site mixing in Li_6_PS_5_I raises the activation barrier for longer-range Li^+^ hopping between neighboring lithium cages and subsequently reduces the bulk ionic conductivity by several orders of magnitude relative to anion-disordered Li_6_PS_5_Cl and Li_6_PS_5_Br.^3,4,7,10^ In order to understand how site mixing impacts molecular dynamics and ion transport in Li_6_PS_5_CN, we constructed six structural models with unique configurations of site disorder across the 4*a*/4*d* sites, as shown in Figure 8. Models for these six configurations were constructed for different (pseudo)halide identities, including *X*^–^ = Cl^–^, CN^–^, Br^–^, and I^–^. Configurations 1 and 6 correspond to structures without anion site mixing, in which the (pseudo)halide occupancy of the 4*d* site is 100% and 0%, respectively. Configurations 2 and 5 correspond to 75%/25% 4*d* (pseudo)halide occupancy, respectively. Configurations 3 and 4 both correspond to 50% (pseudo)halide occupancy at the 4*d* site but with distinct arrangements of the halide/sulfide ions at the corners and faces of the unit cell.

We performed ML-assisted MD simulations for each (pseudo)halide argyrodite (X = Cl^–^, CN^–^, Br^–^, and I^–^) and for each configuration of anion site disorder. We used 2 × 2 × 2 supercells for all simulations as detailed in the Methods section and calculated the average potential energies during our NPT simulation of all six configurations to determine the lowest-energy disorder configuration for each (pseudo)halide identity. The lowest energy disordered configurations also correspond to the smallest lattice volumes determined in these NPT simulations (Figure S11). We take these average potential energies as indicative of relative stability of the configurations with respect to each other. This assignment assumes that the entropic contribution for each configuration is roughly equal and we can therefore take the potential energies as roughly indicative of Gibbs free energies of the configurations. As shown in Figure S10, the lowest average potential energy structures of Li_6_PS_5_Cl and Li_6_PS_5_Br occur for Configuration 3, in which 50% of the 4*d* sites are occupied by halide ions. 50% site mixing in this configuration lowers the average potential energy by 8 meV/atom and 1 meV/atom relative to the ordered Configuration 6 for the chloride and bromide analogs, respectively. In contrast, we find that the lowest-energy configuration for Li_6_PS_5_I occurs for Configuration 6, in which the 4*a* and 4*d* sites are fully occupied by iodide and sulfide, respectively (Figure S10d). This observation is consistent with prior experimental and computational studies which have found that the larger size of iodide prevents I^–^/S^2–^ site mixing at the 4*d* site in the argyrodite structure.^3,4,7,10^ This consistency also supports our use of average potential energy as an indicator of configurational stability. We also note that the dynamical nature of the Li^+^ positions and multiple possible local minima precludes a simpler stability analysis in terms of approximate Gibbs free energies for static structures (e.g., the harmonic approximation from first-principles, as is often done for other materials).^64^

For the cyanide argyrodite Li_6_PS_5_CN, we find that the lowest average potential energy configuration of CN^–^/S^2–^ disorder occurs for Configuration 4 (Figure S10b). We note that we experimentally observe 67(9)% and 32(1)% CN^–^ occupation on the 4*a* and 4*d* sites, respectively, in contrast to the lowest potential energy configuration predicted by our simulations. This discrepancy may be due, in part, to the low X-ray scattering cross-section of C and N relative to higher *Z* species (P, S) and orientational disorder of the cyanides that precludes precise description of their positions from experiment. Alternatively, the deviation could reflect the finite size of the simulation cell or the fact that the average potential energy was used to approximately gauge the stability of a configuration instead of the free energy. As mentioned previously, both Configuration 3 and Configuration 4 exhibit 50% site disorder of the CN^–^/S^2–^ ions at the 4*d* site, however these configurations differ in the arrangement of the CN^–^/S^2–^ ions occupying the faces of the pseudocubic unit cell (the 4*a* site). In Configuration 3, the face of the (100) and (001) planes are occupied by sulfides, while the halides reside on the face of the (010) plane. In Configuration 4, sulfides reside on the faces of the (010) and (001) planes, while halides occupy the face of the (100) plane. Although both configurations display the same degree of site mixing, we suspect that the potential energy differences between Configurations 3 and 4 for the cyanide argyrodite may arise due to interactions between neighboring cyanide ions that favor Configuration 4 or entropic contributions that are not accounted in our average potential energy calculations.^65,66^

#### Lithium Ion Dynamics

**Figure 9:**
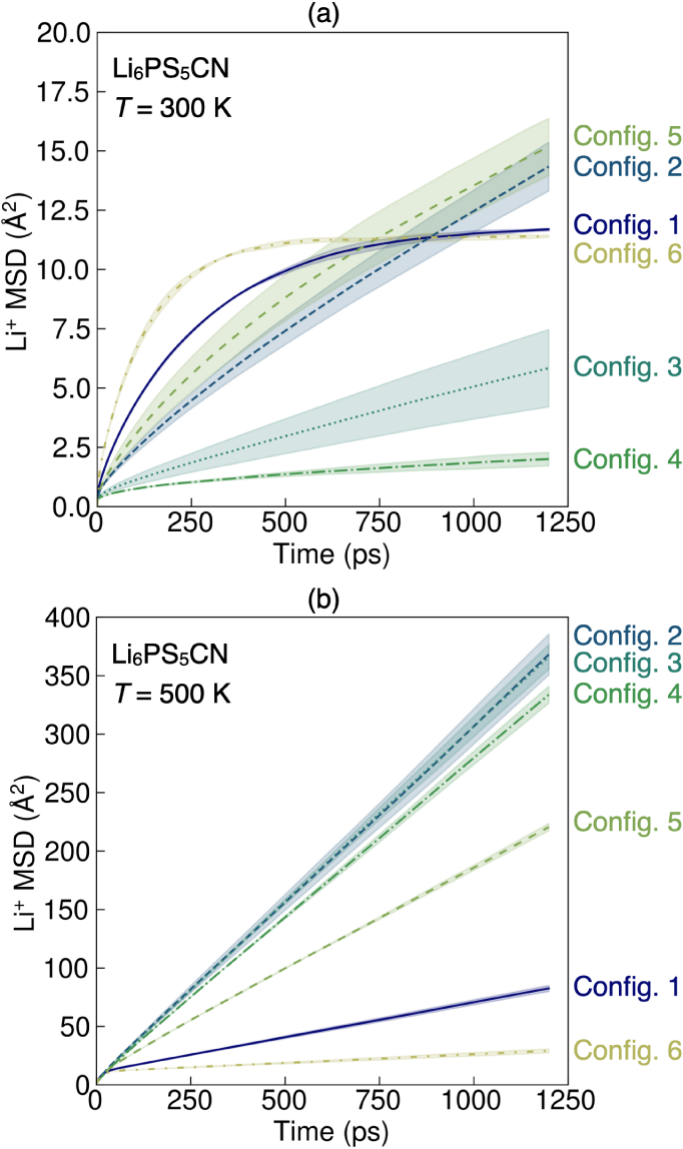
Lithium ion mean squared displacement for each of the six configurations of Li_6_PS_5_CN at (a) *T* = 300 K and (b) *T* = 500 K (see Figure 7). Simulations were performed in triplicate. Lines represent the average MSD from the three runs and the shaded areas represent the standard deviations between replicate runs.

We performed ML-assisted MD simulations from *T* = 300–800 K in 50 K increments and extracted the Li^+^ mean-squared displacement (MSD) over time at each temperature for each (pseudo)halide and each disorder configuration (Figures S13–S23). Representative plots of Li^+^ MSD vs time at both *T* = 300 K and *T* = 500 K for Li_6_PS_5_CN are shown in Figure 9. From inspection of Figure 9, we identify several qualitative trends in Li^+^ dynamics. At *T* = 300 K, we observe that Configurations 1 and 6 (no site disorder) exhibit an initial onset of fast Li^+^ motion over the first ∼250 ps, but then gradually plateau at long times. The plateau has been previously attributed to Li^+^ hopping confined within the lithium cages in the argyrodite structure.^13^ In contrast, when CN^–^/S^2–^ site disorder is present (Configurations 2–5) at *T* = 300 K, the Li^+^ MSDs exhibit positive slopes that are consistent with longer-range diffusion processes. At *T* = 500 K, the higher simulation temperatures facilitate faster long-range diffusion for all configurations. Site-ordered configurations (Configurations 1,6) exhibit shallower slopes than site-disordered Configurations 2–5, which qualitatively indicates higher Li^+^ diffusion coefficients when site mixing is present. These qualitative observations for Li_6_PS_5_CN are consistent with prior studies of the Li^+^ diffusion in argyrodites and the importance of site disorder for longer-range diffusion processes.^11,13^

**Figure 10:**
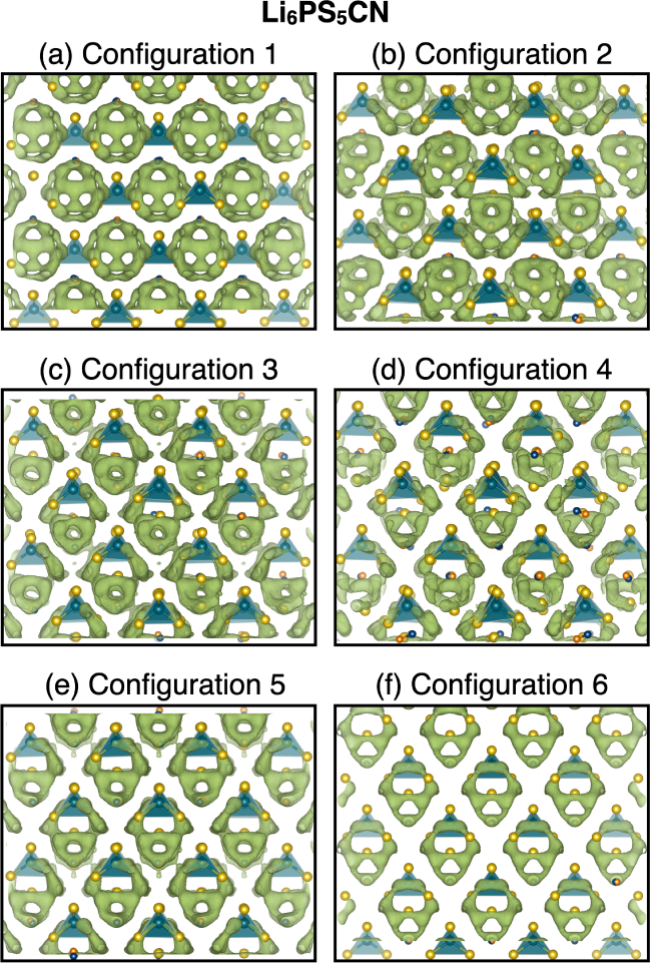
Lithium density distribution determined from molecular dynamics simulations of Li_6_PS_5_CN for each configuration of S^2–^/CN^–^ site mixing at *T* = 300 K. The lithium density isosurfaces, shown in green, are overlaid with the argyrodite structure.

The larger Li^+^ diffusion coefficients observed for site-disordered configurations in the halide argyrodites have previously been attributed to an increase in the frequency of longer-range lithium hopping between neighboring lithium cages.^4,6,11−13^ As shown in the lithium density distributions in Figure 10 at *T* = 300 K, the Li_6_PS_5_CN structures with S^2–^/CN^–^ ordering (Configurations 1 and 6) exhibit highly localized Li^+^ densities which indicates that lithium diffusion is predominantly confined to short-range hopping within the lithium cages. In contrast, anion disorder (Configurations 2–5) results in more disperse lithium ion densities that are reflective of a higher frequency of intercage jumps that contribute to long-range diffusion and higher lithium self-diffusion coefficients. We observe similar differences in the lithium density distributions for ordered vs disordered configurations of the other halide argyrodites, as shown in Figures S25–S27.

In order to quantify the lithium ion diffusion behavior, we extracted the lithium ion self-diffusion coefficients (*D*_Li_) from the MD simulations, which describes the rate at which individual lithium ions diffuse through the structure.^13,67^ Lithium ion self-diffusion coefficients were determined at each simulation temperature, for each (pseudo)halide, and for each disorder configuration from the slope of the MSD in the long-time limit (further details are provided in the Supporting Information). The extracted Li^+^ diffusion coefficients are shown in Figure S24 on Arrhenius plots and are tabulated in Tables S2–S4. At higher temperatures (*T* ≥ 500 K), we find that the diffusion coefficients obey the Arrhenius relationship (Figure S24) for all (pseudo)halides and configurations of site disorder. At *T* = 500 K, this yields lithium ion diffusion coefficients on the order of *D*_Li_ ∼ 10^–6^ cm^2^ s^–1^ (Configurations 2–5) and *D*_Li_ ∼ 10^–7^–10^–8^ cm^2^ s^–1^ (Configurations 1 and 6) for all (pseudo)halides. These values are comparable to prior computational reports of lithium ion diffusion in halide argyrodites.^12,68^ At lower temperatures and for site-ordered models, the generally slower Li^+^ diffusion often results in MD simulations that have not fully converged over the 1200 ps duration of the simulations (e.g., the slope of Li^+^ MSD vs time is not constant); this manifests as deviations from the Arrhenius relationship at lower temperatures (Figure S24). As such, there is significant uncertainty in diffusion coefficients with values below 10^–7^ cm^2^ s^–1^ from our simulations.

**Figure 11:**
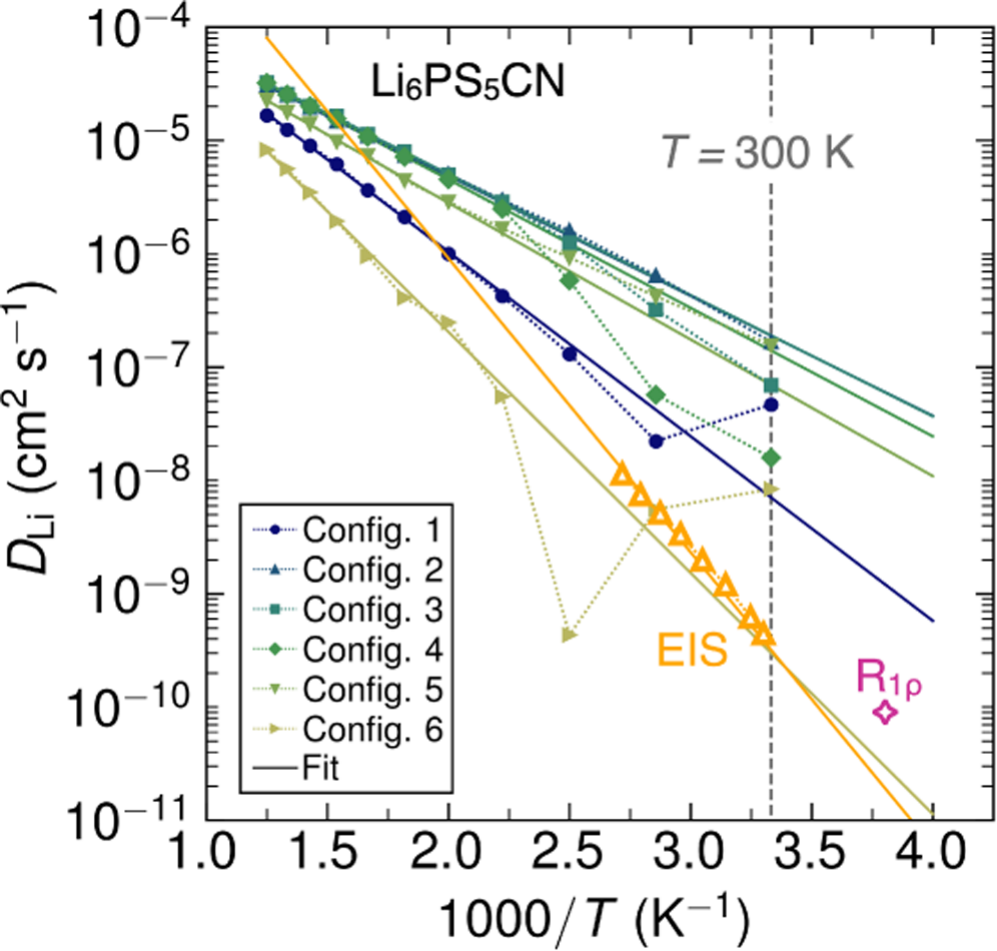
Comparison of diffusion coefficients determined by MD simulations, ^7^Li SSNMR relaxometry, and AC electrochemical impedance spectroscopy. Li^+^ diffusion coefficients from MD simulations are shown as filled symbols for each Configuration of site disorder, and dotted lines are provided as a guide to the eye. The magenta star represents the Li^+^ diffusion coefficient determined from SS NMR relaxometry in the rotating frame (*R*_1ρ_, 45 kHz). Li^+^ diffusion coefficients calculated from temperature-dependent AC electrochemical impedance spectroscopy (EIS) are shown as open orange triangles. For all data sets, solid lines represent linear regressions based on the relationship *D*_Li_ = *D*_0_ exp(−*E*_A_/*k*_B_*T*).

Comparison of the experimental and computational Li^+^ diffusion coefficients provides a holistic description of the Li^+^ transport behavior of Li_6_PS_5_CN. In Figure 11, we compare the Li^+^ diffusion coefficients from ^7^Li SS NMR relaxometry, AC EIS, and MD simulations. Li^+^ diffusion coefficients were determined from the EIS data via the Nernst–Einstein equation^30,51^
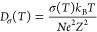
4where *N* is the number density of Li^+^ (2.5 × 10^28^ m^–3^), *e* is the elementary charge, *Z* is the charge of Li^+^, and σ(*T*) is the ionic conductivity of Li_6_PS_5_CN determined from EIS measurements. For simplicity, we assume a Haven ratio of 1 for uncorrelated motion and we assume that all lithium ions are mobile. From this calculation, we find that the diffusion coefficients of Li_6_PS_5_CN are in close agreement between both EIS and SS NMR. For example, we find a value of *D*_σ,303K_ = 4.7 × 10^–10^ cm^2^ s^–1^ for bulk lithium ion diffusion at *T* = 30°C (*T* = 303 K). Extrapolating the EIS Arrhenius relationship to lower temperatures yields *D*_σ,263K_ = 2.98 × 10^–11^ cm^2^ s^–1^, which is closely comparable to the diffusion coefficient of *D*_263K_ = 8.97 × 10^–11^ cm^2^ s^–1^ determined from SS NMR relaxometry (*R*_1ρ_, Figure 7). EIS and NMR relaxometry in the rotating frame (*R*_1ρ_) both probe ion transport occurring over long length- and time-scales,^11,13,46,60^ and thus we expect these measurements to be in relatively close agreement. While the experimentally determined diffusion coefficients are in good agreement, we find that the diffusion coefficients from MD simulations generally overestimate the experiment, though the degree of the overestimation varies (e.g., it amounts to less than 2 orders of magnitude for Configuration 4). This overestimation, particularly at low temperatures (*T* = 300 K), has been previously attributed to the presence of defects and heterogeneities in real samples that contribute to macroscopic transport but are not well-captured by the relatively short time- and length-scales of our MD simulations.^69^ At higher temperatures (*T* ≥ 500 K), however, the diffusion coefficients from both MD simulations and extrapolated from EIS measurements are in substantially better agreement, with diffusion coefficients in the ≥10^–6^ cm^2^ s^–1^ regime.

#### Cyanide Ion Dynamics

**Figure 12:**
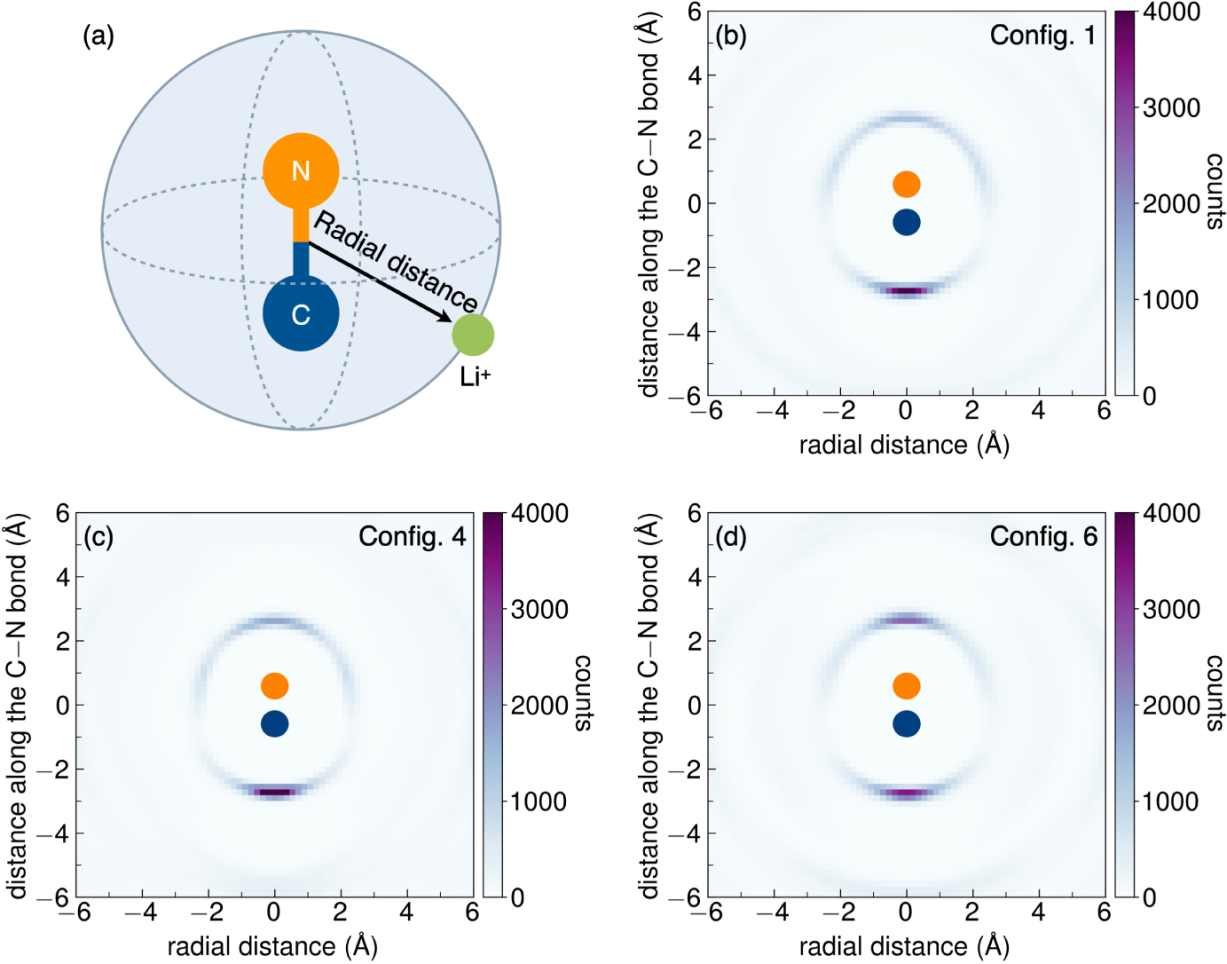
(a) A cartoon diagram representing the PSDF calculations for Li_6_PS_5_CN taken from 12 ns MD trajectories at *T* = 300 K. Lithium ions were tracked relative to the origin at each time step in the simulation. The radial distance from the Li^+^ to the center of the CN bond (at the origin) was recorded and projected into a 2D color plot shown in (b)–(d) for Configurations 1, 4, and 6 respectively. The frequency of occurrence at a particular radial distance is represented in “counts” in the color map. In each configuration, the lithium ions show a preference for aligning along the C–N axis, particularly near the carbon.

We calculated a pseudospatial distribution function (PSDF) for lithium relative to cyanide from our MD simulations to further uncover the potential for Li-CN coupling in Li_6_PS_5_CN. PSDFs were calculated for ordered (Configurations 1 and 6) and disordered (Configuration 4) structures of Li_6_PS_5_CN from MD simulations performed at *T* = 300 K. As illustrated by the schematic in Figure 12a, the frame of reference is fixed with respect to the cyanide and the relative positions of the lithium ions are taken from each time step in the MD simulations. The radial distance from the lithium ions to the origin (the center of the C–N bond) was determined for each time step of the simulation and the three-dimensional radial vectors were projected onto the plane of the cyanide ion as shown in the color maps in Figure 12b–d. The frequency of Li^+^ occurrence at a particular radial distance is represented in “counts” in the color map. For all configurations, we observe that lithium ions preferentially reside at positions directly along the C–N bond axis with a higher affinity for the carbon atom, which points to electrostatic coupling between the lithium and cyanide ions in Li_6_PS_5_CN. Interestingly, we observe higher Li^+^ density at both ends of the cyanide molecule, despite the fact that the carbon atom carries the negative charge in cyanide. We attribute this observation to the ambident nature of the cyanide ion and the unpaired electrons in the sp orbital on both the carbon and nitrogen atoms that protrude outward along the bond axis.^29^ The distinct distribution of lithium ions due to electrostatic coupling with cyanide anions is reflected in the broad radial distribution function of the Li–CN interactions compared to the single, well-defined Li–*X* distance observed in the halide analogs (Figures S28 and S29).

**Figure 13:**
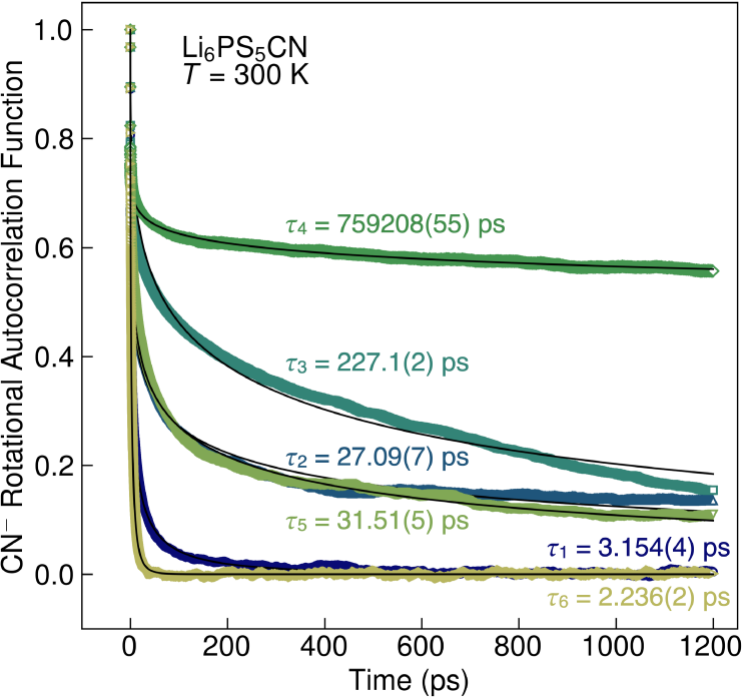
Rotational autocorrelation function for cyanide ions calculated for all Configurations of anion site disorder from MD simulations performed at *T* = 300 K. The autocorrelation functions were fit to a stretched exponential, , to determine the time constants for cyanide reorientations (τ). Data are shown as colored markers and the fits to the stretched exponential function are shown as black lines.

MD simulations provide further insight into the presence and time scales of cyanide rotational dynamics in Li_6_PS_5_CN. We extracted the rotational autocorrelation function for cyanide from MD simulations at *T* = 300 K for each configuration of anion disorder, as shown in Figure 13. The autocorrelation functions were fit to a stretched exponential decay, , which has been previously used to extract the time scales of cation rotational dynamics in hybrid perovskites.^45^ We find that the configuration of anion disorder significantly impacts the presence and time scales of cyanide rotational dynamics in Li_6_PS_5_CN. For Configurations 1 and 6, we observe a rapid decay of the autocorrelation function that corresponds to time constants of τ_1_ = 3.154(4) ps and τ_6_ = 2.236(2) ps, respectively. These time scales correspond to fast reorientation frequencies of 3.2 × 10^11^ Hz and 4.5 × 10^11^ Hz, respectively. In contrast, anion site disorder results in substantially slower cyanide rotational dynamics. Configurations 2 and 5 correspond to 75% CN^–^ and 25% CN^–^ occupation on the 4*d* site, respectively, and exhibit very similar time scales of cyanide rotational dynamics with time constants of τ_2_ = 27.09(7) ps (3.7 × 10^10^ Hz) and τ_5_ = 31.51(5) ps (3.2 × 10^10^ Hz), which are quite similar to cyanide reorientation rates of 2 × 10^10^ Hz reported in K(CN)_1–*x*_Br_*x*_.^70^ For 50% site mixing (Configurations 3, 4), we find substantially slower cyanide rotational time constants of τ_3_ = 227.1 ps (4.4 × 10^9^ Hz) and τ_4_ = 759208(55) ps (1.3 × 10^6^ Hz). We note that the rotational autocorrelation function does not decay to zero over the duration of our 1200 ps simulations for Configurations 2–5, but rather exhibits an initial decay and then plateaus. Thus, the time constants and frequencies extracted from these simulations (particularly for Configuration 4) are not a reliable indicator of an actual reorientation time but rather signify that cyanides are orientationally constrained and do not rotate fully freely over the duration of the simulations. The initial decay at short time scales may be attributed to nonrotational dynamics of the cyanide ion, such as “wobbling in a cone” motions.^71^ Furthermore, the impact of site disorder on cyanide dynamics appears to be conserved at higher simulation temperatures. At *T* = 500 K (Figure S30), we observe faster initial decay of the rotational autocorrelation function at short time scales, consistent with faster reorientational dynamics of the cyanide ions in Li_6_PS_5_CN for all configurations. However, we observe that, for configurations with site disorder (Configurations 2–5), the autocorrelation function never decays to zero over the duration of the simulations. This behavior indicates that cyanides are permitted to undergo full rotations in all configurations at *T* = 500 K, but that the rotations are not fully isotropic when site disorder is present.^72 −75^ The sluggish or frozen cyanide dynamics at lower temperatures (*T* ≤ 300 K) are consistent with temperature-dependent SXRD and DSC measurements which indicate that Li_6_PS_5_CN does not undergo crystallographic or thermodynamic phase transitions over the measured temperatures. Together, these data suggest that Li_6_PS_5_CN exhibits glassy behavior with hindered cyanide rotations at and below room temperature.^35,36,70,76,77^

## Discussion

The configuration of (dis)order between the cyanide and sulfide anion sublattices strongly impacts lithium ion diffusion rates in Li_6_PS_5_CN. Our MD simulations reveal that cyanide/sulfide anion site disorder promotes fast Li^+^ diffusion, while slower Li^+^ diffusion is observed for structures with ordered anionic sublattices. This behavior is consistent with prior experimental and computational studies of the impact of anion disorder on ion transport in halide argyrodites,^4,6,11−13^ and indicates that site disorder is critically important for bulk transport processes in Li_6_PS_5_CN.

Furthermore, we find that anion site disorder dictates the presence and time scales of cyanide rotational dynamics in Li_6_PS_5_CN. In the ordered configurations (Configurations 1 and 6), cyanide undergoes rapid reorientational dynamics on time scales of ∼10^11^ Hz at *T* = 300 K, similar to the time scales of cyanide dynamics observed in prior studies of K(CN)_1–*x*_Br_*x*_.^70^ In contrast, CN^–^/S^2–^ site disorder leads to slow or inhibited cyanide reorientational dynamics. The sluggish or frozen cyanide dynamics are consistent with temperature-dependent SXRD and DSC measurements which indicate that Li_6_PS_5_CN does not undergo crystallographic or thermodynamic phase transitions over the measured temperatures.

Given the rotational dynamics of cyanide (Figure 13) and the observation of electrostatic coupling between Li^+^ and CN^–^ ions (Figure 12), it is interesting to consider dynamic Li–CN coupling in Li_6_PS_5_CN. Using the Einstein–Smoluchowski equation,  (where *a* is the jump distance, here approximated to be 3.1 Å),^25^ we determine a lithium ion jump rate 1/τ of ∼2.8 × 10^6^ Hz from the EIS measurements performed at *T* = 300 K. In order to infer the potential presence of coupled Li–CN dynamics, we compare this experimental Li^+^ jump rate to the time scales of cyanide rotations determined from MD simulations. In ordered Configurations 1 and 6, cyanides undergo quasi-free rotations on time scales of ∼10^11^ Hz (∼2–3 ps) at *T* = 300 K, and we therefore anticipate that Li^+^ hopping and CN^–^ rotations are fully decoupled and only experience transient electrostatic interactions that manifest as the Li–CN coupling observed in Figure 12. In contrast, the presence of CN^–^/S^2–^ site mixing results in slow or hindered cyanide rotations depending on the degree and configuration of site disorder. For Configurations 2 and 5 (25%/75% site disorder) this yields rotations on the order of ∼10^10^ Hz, while for Configuration 3 and 4 (50% site disorder) we observe even further hindered rotational dynamics. These slower rotational dynamics are still faster than the time scales for bulk Li^+^ transport. We note that the time and length scales accessed by the present MD simulations are likely not yet large enough to safely access Li^+^ diffusion coefficients of around 10^–10^ cm^2^ s^–1^ as seen in EIS and NMR or to capture cyanide rotational dynamics beyond the 12 ns duration of the simulations. Substantial increases in MD simulation capability, beyond the scope of the present work, are still needed to fully resolve all atomistic details of relatively low-*T* Li^+^ diffusion and cyanide dynamics in Li_6_PS_5_CN. However, the present data suggest that cyanide dynamics and Li^+^ hopping generally occur on distinct time scales at *T* = 300 K.

**Figure 14:**
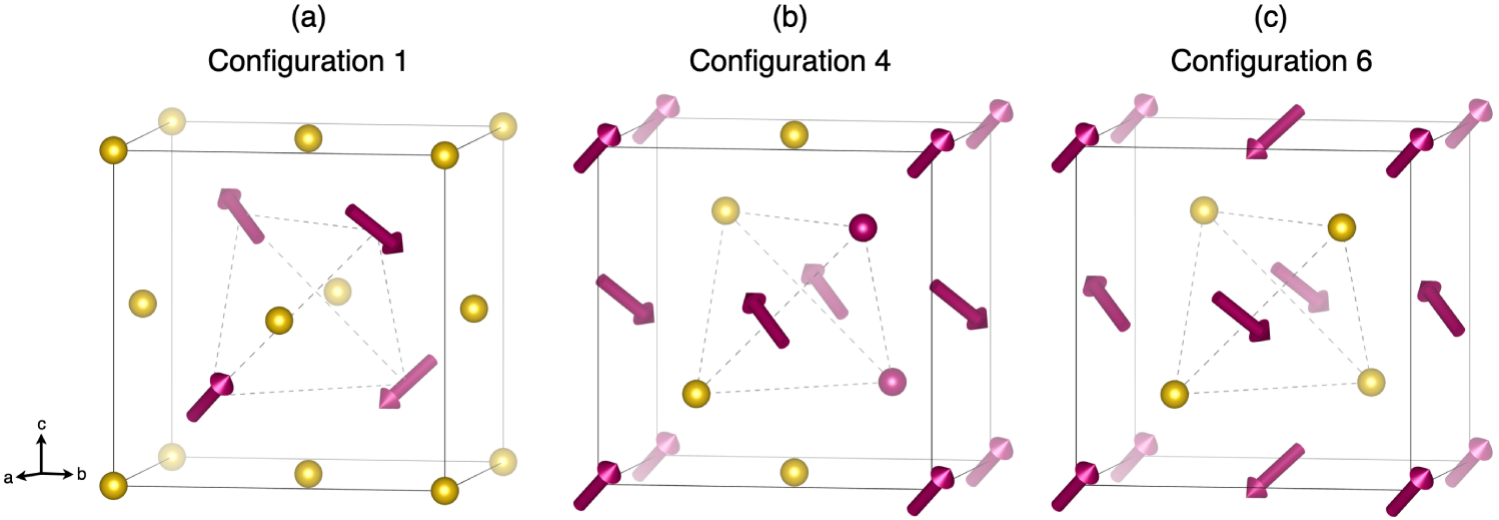
Depiction of cyanide elastic dipoles arranged on the argyrodite structure for Configurations 1 (a), 4 (b), and 6 (c). Sulfide ions are represented by yellow spheres, and cyanide elastic dipoles and orientations are represented by magenta vector arrows.

In order to understand why site disorder impacts cyanide dynamics in Li_6_PS_5_CN, we first consider how interactions between neighboring cyanides are affected by the local configuration of anion (dis)order. The interaction energy between two elastic dipoles in a solid has been previously described by
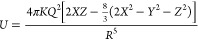
5where *Q* is the elastic dipole moment, *K* is the bulk modulus of the solid, and *R* is the distance between elastic dipoles.^66^ Pfeiffer and Mahan expanded this expression to elastic dipoles arranged on a face-centered cubic (FCC) lattice and demonstrated that the interaction energy is minimized when nearest-neighbor elastic dipoles are oriented in a “T” configuration.^66^ These interactions are, in part, responsible for the formation of ordered ground states in alkali cyanides at low temperatures.^37,38,70^ The anion sublattice of the argyrodite family can be considered as two interpenetrating FCC lattices offset by a translation vector of (, , ). In order to understand the impact of cyanide–cyanide interactions on their rotational dynamics in Li_6_PS_5_CN, we map the ground-state ordered dipole configurations onto the interpenetrating anion FCC sublattices in the argyrodite structure for both ordered (Configurations 1 and 6) and disordered (Configuration 4) configurations (Figure 14). In Configurations 1 and 6, the cyanide ions reside on the lattice points to an FCC lattices originating at (0.75, 0.25, 0.25) and (0, 0, 0), respectively, with nearest-neighbor cyanide–cyanide distances of *R* ∼ 7.0 Å. Figure 14a,c illustrates the minimum potential energy arrangements of the elastic dipoles on an FCC lattice for ordered configurations (Configurations 1 and 6).

In anion-disordered configurations, site mixing of cyanide and sulfide anions disrupts the ground state arrangement of cyanide elastic dipoles on the two FCC anion sublattices. As shown in Figure 14b, 50% of the 4*d* sites are occupied with cyanides at (0.25, 0.75, 0.25) and (0.75, 0.75, 0.75) in Configuration 4, with substantially shorter nearest-neighbor cyanide–cyanide distances (*R* ∼ 4.30 Å compared to *R* ∼ 7.02 Å in the ordered configurations). Closer cyanide–cyanide distances and the strong dependence (1/*R*^5^) of the elastic dipole interaction energy will strongly impact elastic dipole coupling between neighboring cyanides when anion site disorder is present in Li_6_PS_5_CN. While the longer cyanide–cyanide distances in Configurations 1 and 6 permit fast, quasi-free rotation of the cyanide ions on time scales of 10^11^ Hz, we hypothesize that strong dipole–dipole coupling in anion-disordered Configurations (Configurations 2–5) may inhibit reorientations and lead to the restricted rotational dynamics observed in our MD simulations. We further consider how the degree of site mixing could impact elastic dipole coupling and the timescales of cyanide rotational dynamics. As shown in Figure 13, increasing the degree of anion site mixing results in the most hindered rotational dynamics of the cyanide anions (e.g., 50% site mixing in Configurations 3 and 4 results in a slower decay of the rotational autocorrelation function compared to 75%/25% site mixing in Configurations 2 and 5). This observation suggests that the interactions between neighboring cyanides play a significant role in the presence and time scales of rotational dynamics in Li_6_PS_5_CN.

Site disorder may also lead to differences in local strain that can modulate the potential energy landscape for molecular reorientations. Site disorder and local strain have been previously invoked to explain glassy dynamics in alkali cyanides such as K(CN)_1–*x*_Br_*x*_, where dilution of the cyanide sublattice with bromide leads to the formation of an orientationally disordered dipole glass at low temperatures due to fluctuations in the local potential surrounding the cyanide anions.^33,35,36,70,78^ Furthermore, Brinek et al. recently demonstrated that structural disorder in Li_6_PS_5_I slows the rotational dynamics of the PS_4_^3–^ tetrahedra^27^ and effectively decouples the PS_4_ rotations from lithium ion hopping.^25^

Taken together, we find that site disorder between the CN^–^/S^2–^ sublattices (rather than rotational dynamics of CN^–^) is predominantly responsible for promoting long-range diffusion processes in Li_6_PS_5_CN. The distinct time scales of cyanide rotations and lithium ion hopping suggest that these processes are decoupled, though we observe Li–CN interactions due to transient electrostatic coupling of these species in the structure. As the time scales of Li^+^ hopping and CN^–^ rotational dynamics are quite sensitive to the local configuration of CN^–^/S^2–^ site disorder, we propose that there may exist a configuration of site disorder in which these processes occur on similar time scales. Further tailoring this disorder is therefore a promising future direction to impact Li^+^ diffusion and the proclivity for coupled Li–CN dynamics in Li_6_PS_5_CN.

## Conclusions

We present a suite of experimental and computational measurements, including temperature-dependent synchrotron powder X-ray diffraction, AC electrochemical impedance spectroscopy, ^7^Li SSNMR, and molecular dynamics simulations, in order to understand the complex interplay of site disorder, ion diffusion, and cyanide dynamics in the cyanide argyrodite Li_6_PS_5_CN and the halide analogs Li_6_PS_5_X (X = Cl^–^, Br^–^, I^–^). We find that the configuration of anion site mixing between *X*^–^/S^2–^ enables long-range Li^+^ transport processes across all (pseudo)halide identities, which points to the crucial role of anion site disorder in the Li^+^ conductivity in this family of solid-state electrolytes. Furthermore, anion (dis)order plays a decisive role in both ion diffusion and cyanide dynamics in the cyanide argyrodite. In anion-ordered configurations of Li_6_PS_5_CN, cyanides undergo fast, quasi-free rotations at *T =* 300 K, and we observe preferential orientation of the Li^+^ ions at the ends of the cyanide anion, which may suggest transient electrostatic coupling. In contrast, CN^–^/S^2–^ site disorder produces substantially slower or simply quenches cyanide rotational dynamics in Li_6_PS_5_CN, possibly due to closer proximity of neighboring cyanides that interact strongly through elastic dipole coupling or through differences in local strain fields in which the cyanide ions reside. Furthermore, we find that cyanide dynamics generally occur on faster time scales than Li^+^ hopping that likely preclude fully coupled Li–CN motions. We further propose that judicious control of the CN^–^ sublattice may be leveraged as a design principle to direct the time scales of both Li^+^ hopping and cyanide rotational dynamics in Li_6_PS_5_CN.

## Methods and Materials

### Materials Synthesis

The cyanide argyrodite Li_6_PS_5_CN was synthesized by the low-temperature solution-phase methods described elsewhere.^23^ Briefly, 1.2 mmol of P_2_S_5_ and 3.6 mmol of Li_2_S were added to 20 mL of dry tetrahydrofuran (THF) in a 100 mL Schlenk flask and stirred for 20 min to form a bright yellow suspension. To a separate 100 mL Schlenk flask, 2.4 mmol of LiCN·DMF and 2.4 mmol of Li_2_S were dissolved in 20 mL of dry ethanol. The ethanolic solution was cannula transferred into the THF solution to yield an emerald green transparent solution. The final product was recovered by evaporating the THF and ethanol at gradually increasing temperatures. The product was dried on the vacuum line at 150 °C for one hour.

### Characterization

#### Structural Characterization

High-resolution synchrotron powder X-ray diffraction (SXRD) data were collected on the 11-BM-B beamline at the Advanced Photon Source, Argonne National Laboratory. Powdered samples of Li_6_PS_5_CN were packed into quartz capillaries (OD: 0.70 mm, ID: 0.69 mm). The capillaries were sealed with two-part epoxy and then sheathed inside 0.8 mm Kapton capillaries and sealed with a clay plug to prevent air exposure. We first collected data on cooling from *T* = 300 K to *T* = 90 K and then upon heating from *T* = 90 K to *T* = 300 K in 10 K increments. For the quenched scans, a pristine sample capillary was loaded into the instrument. The cryostream was set to *T =* 90 K and then loaded onto the sample to cool the sample as quickly as possible. The sample was allowed to equilibrate for 5 min prior to collecting data upon heating from *T* = 90 K to *T =* 300 K in 10 K increments. All diffraction data were analyzed by the Rietveld method implemented in TOPAS v6. VESTA was used to visualize and render all crystal structures presented in this publication.^79^

#### Differential Scanning Calorimetry

Differential scanning calorimetry data were collected with a TA Instruments DSC 250 calorimeter equipped with liquid nitrogen cooling. Polycrystalline powder of Li_6_PS_5_CN (mass = 6.8 mg) was loaded into an aluminum *T*_0_ hermetic sample pan and sealed in an argon-filled glovebox. An empty Al sample pan was used as a reference. Data were collected at heating/cooling rates of 5 K min^–1^, as shown in Figure S1. Quenching studies were performed by ramping the temperature to *T* = −90 °C (*T* = 183 K).

#### AC Electrochemical Impedance Spectroscopy

Bulk ionic conductivity and Li^+^ ion migration energy barriers were calculated from temperature-dependent potentiostatic electrochemical impedance spectroscopy (EIS). Inside an argon-filled glovebox, a 6 mm diameter pellet of ∼0.1 g of Li_6_PS_5_CN was pressed uniaxially to ∼60 bar on the gauge, which is equivalent to 8000 bar pressure over the area of the pellet. Pressing resulted in a pellet ∼2 mm thick with ∼80% theoretical density. Gold blocking electrodes were sputtered onto both sides of the pellet at 13 mA for 40 s. The pellet was loaded into a home-built, air-free, insulating polyether ether ketone (PEEK) cell in parallel plate capacitor geometry with 6 mm diameter stainless steel rods in contact with the gold electrodes. Measurements were performed under 16 MPa of constant pressure as measured by an in-line load cell in a home-built pressure jig. EIS measurements were collected using a Gamry Interface 1010E Potentiostat with an applied bias of 20 mV. The frequency was swept logarithmically from 2 × 10^6^ Hz to 0.2 Hz at 10 measurements per decade. The PEEK cell, pressure jig, and load cell assembly were placed in a Quincy Lab convection oven for temperature-dependent measurements. The sample was equilibrated at each temperature point for several hours, and sample temperature equilibration was determined by ensuring that the impedance was no longer evolving over time. The EIS data were modeled with an (*R*_1_*Q*_1_) + *Q*_2_ equivalent circuit using *ad hoc* Python code to extract parameters descriptive of the impedance.

#### ^7^Li Solid State Nuclear Magnetic Resonance Spectroscopy

^7^Li SSNMR measurements were performed on a 200 MHz (4.7 T) Bruker AvanceIII HD NMR spectrometer, which corresponds to a^7^Li Larmor frequency of 77.76 MHz, equipped with a 7 mm HX DVT style probe. Three data sets were collected at temperatures ranging from −100 °C to +120 °C: single pulse excitation (SPE), ^7^Li spin–lattice relaxation (SLR) in the laboratory frame (*T*_1_) using saturation recovery method, and ^7^Li SLR in the rotating-frame (*T*_1ρ_) using a spin-lock frequency of 45 kHz. For all measurements, hard 90° excitation pulses of 3.125 μs were used. SPE measurements were performed using a 60 s recycle delay, 4 dummy scans and 8 scan averages. SLR *T*_1_ measurements were performed using the saturation recovery method, which employed a train of 32 90° pulses separated by short (10 μs) delays followed by a variable recovery delay ranging from 0.1 to 20 s, and a readout pulse.^49^ SLR *T*_1ρ_ measurements were performed using an initial 90° excitation pulse followed by a variable-length spin-locking pulse (ranging from 0.1 to 30 ms) on the ^7^Li channel at a field strength of 45 kHz, followed by acquisition, with a recycle delay set to 30 s. The temperature was controlled by passing nitrogen gas through a liquid nitrogen heat exchanger for measurements below room temperature, or with the standard probe heater for high-temperature measurements, using a flow rate of 1000 Lh^–1^. Temperature at the probe was calibrated using lead nitrate.^80^ To ensure measurements were conducted under inert conditions, samples were first loaded into flat-bottomed Pyrex ampules (4.57 mm ID, 5.59 mm OD) in an Ar-filled glovebox and flame-sealed under vacuum. Ampules were then loaded into standard Bruker 7 mm MAS rotors and were inserted into the probe. No sample spinning was performed.

#### All-Electron Full Potential van der Waals Corrected Density Functional Theory

All the first-principles calculations were performed using van der Waals-corrected density functional theory (DFT). Specifically, DFT calculations were carried out using the all electron FHI-aims code^61^ with the PBE functional^81^ and the nonlocal many body dispersion (MBD-NL) version of the van der Waals correction.^82^ To save computational cost, the “light” settings from the 2020 default in FHI-aims^61^ were used for all calculations, with only the Γ point used for *k*-point sampling. The settings and the *k*-point sampling were validated against the more accurate “intermediate” settings with a 4 × 4 × 4 *k*-point sampling, as illustrated in Figure S9.

#### Machine Learning Potential with the Moment Tensor Potential Form

The development of machine learning interatomic potentials (MLIP) has enabled efficient sampling of potential energy surfaces with first-principles accuracy.^83^ Among the MLIPs, the moment tensor potential (MTP) introduced by Shapeev, which uses tensors of inertia of the system of neighboring atoms, has been shown to be relatively accurate and data-efficient.^62,63,84^ A recent study by Qi et al. has demonstrated the effectiveness of MTP for simulating lithium conductivity in various types of lithium superionic conductors.^85^ Therefore, in this work, we have chosen MTP as our MLIP, following the technical choices made by Qi et al., with a cutoff radius, *R*_cut_ of 5.0 Å and the parameter *lev*_*max*_ (maximal level of moments) set to 16 to control the number of the basis functions.

**Figure 15:**
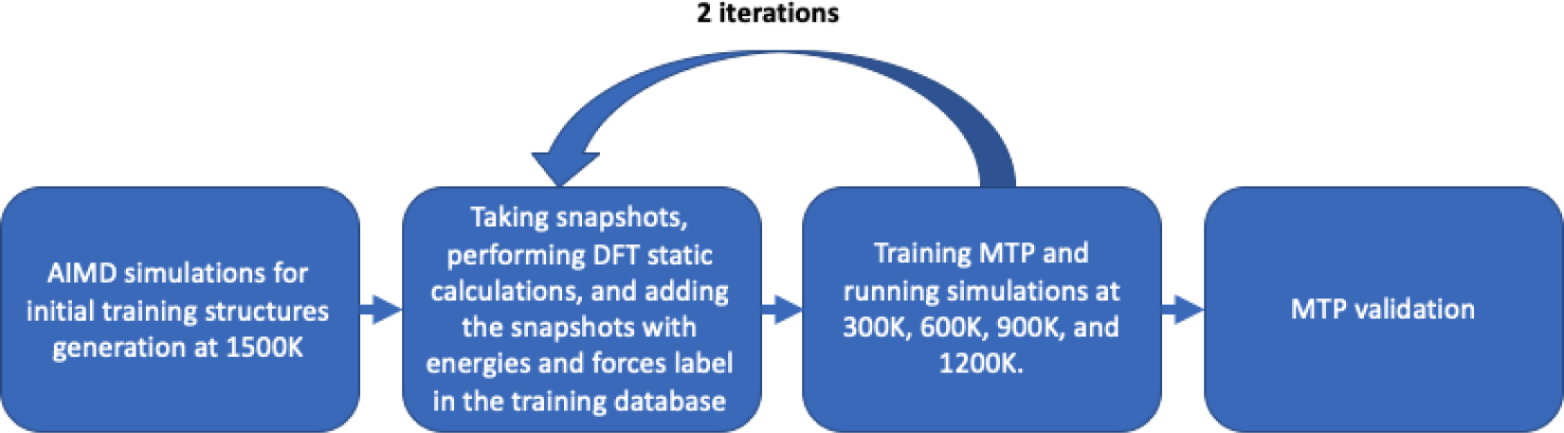
Flowchart of the iterative training database preparation and MTP training scheme for halide argyrodites with different configurations of site disorder.

To construct an MLIP, it is necessary to gather first-principles data. An optimal strategy to construct an MLIP with the first-principles method is still an active research area with ongoing developments.^86^ Following the approach suggested by Miksch et al.,^87^ our strategy involves an iterative process for constructing the MLIP and compiling the reference first-principles data, as summarized in Figure 15. Initially, we performed *ab initio* molecular dynamics simulations at *T* = 1500 K to collect the training data. The construction of the initial structures was begun from prototype structures obtained from the Materials Project^88^ with the ID numbers mp-985592, mp-985591, and mp-985582, corresponding to Li_6_PS_5_Cl, Li_6_PS_5_Br, Li_6_PS_5_I, respectively. By substituting the Cl atom in Li_6_PS_5_Cl structure with the CN molecule, we obtained the Li_6_PS_5_CN structure. Subsequently, we manually swapped the coordinates of the S atom and (pseudo)halide atoms to generate the structures in Configurations 1–6, as shown in Figure 8. We used a 2 × 2 × 2 supercell which gave around 20 Å × 20 Å × 20 Å cell size with 416 atoms for Li_6_PS_5_Cl, Li_6_PS_5_Br, Li_6_PS_5_I and 448 atoms for Li_6_PS_5_CN following the technical choices of Qi et al.^85^ In the initial training structures generation step, 10 ps molecular dynamics were performed for each configuration with a time step of 2 fs under the NVT ensemble using the Bussi-Donadio-Parrinello thermostat,^89^ at the experimental cell size and at −5%, −2.5%, +2.5%, and +5% of the experimental cell sizes, resulting in a total of 300 ps initial trajectories (6 configurations × 5 cell size × 10 ps). A high temperature of *T* = 1500 K was chosen for the initial trajectories to allow for exploration of a larger configuration space, reducing the occurrence of instability during MLIP simulations, as suggested by Miksch et al.^87^ Using the MLIP trained on the initial training structures, we performed additional simulations at *T* = 300, 600, 900, and 1200 K. Snapshots from these simulations were used for single point calculations and the resulting data points were included in the training structures to retrain the MLIP. This iterative process was repeated twice to ensure convergence of the MLIP. Importantly, we trained a single MLIP for all the six configurations corresponding to each composition, allowing us to make direct comparisons among configurations.

The productive molecular dynamics simulations were performed in NPT ensemble using the Bussi-Donadio-Parrinello thermostat^89^ and the Berendsen barostat.^90^ Simulations were carried out at temperatures from *T* = 300 to 800 K, with 50 K intervals, and a pressure of 1 bar. A time step of 2 fs was used, and each simulation was run for 4 ns. To ensure statistical accuracy, each system was simulated three times, resulting in a total of 12 ns of trajectory data for properties calculations. All training, evaluations, and simulations with MTP were performed with the MLIP-2 package^62^ and the LAMMPS package.^91^ Customized scripts were developed to facilitate the conversion between the output file format of FHI-aims,^61^ the geometry.in file format^92^ of FHI-aims, and the MLIP cfg format.

#### Rotational Dynamics Calculation

The rotational autocorrelation function^93^ is employed to characterize the rotational dynamics of the CN molecule. In our analysis, we define **p**_CN_ as the vector pointing from the N atom to the C atom within the molecule. Subsequently, we calculate the second-order Legendre polynomial (*P*_2_) of the autocorrelation function for **p**_CN_, using the following equation:
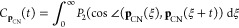
6

## Supporting Information Available

Machine learning scatter plots for Li_6_PS_5_*X* (*X* = CN^–^, Cl^–^, Br^–^, I^–^), plots of potential energy vs configuration and lattice parameter vs configuration for Li_6_PS_5_*X* (*X* = CN^–^, Cl^–^, Br^–^, I^–^) calculated by averaging the machine learning-assisted molecular dynamics simulations within NPT ensemble, temperature-dependent lattice parameters for Li_6_PS_5_*X* (*X* = CN^–^, Cl^–^, Br^–^, I^–^) calculated by averaging the machine learning-assisted molecular dynamics simulations within the NPT ensemble, a description of the mathematical relationships between lithium mean-squared displacement and diffusion coefficient, lithium mean-squared displacement for simulation temperatures between *T* = 300–800 K in 50 K increments, Arrhenius plot for lithium ion self-diffusion coefficients from the molecular dynamics simulations, lithium density distributions for Li_6_PS_5_*X* (*X* = Cl^–^, Br^–^, I^–^) from MD simulation trajectories, tabulated diffusion coefficients for each Li_6_PS_5_*X* compound (*X* = CN^–^, Cl^–^, Br^–^, I^–^) from MD simulations, radial distribution functions for all Li–*X* distances extracted from the MD simulation trajectories, and the cyanide rotational autocorrelation function calculated from MD simulations at *T* = 500 K (PDF)
A TOPAS input file for Rietveld refinements, differential scanning calorimetry data of Li_6_PS_5_CN, fitting parameters from AC electrochemical impedance spectroscopy measurements, and fitting routines and parameters for ^7^Li SSNMR linewidth and relaxometry analyses (TXT)
