# Supplementary material for: Site Disorder Drives Cyanide Dynamics and Fast Ion Transport in Li6PS5CN
Source: Chem Mater. 2024 Sep 25;36(19):9424–41. doi: 10.1021/acs.chemmater.4c00979 (PMC11467833; doi:10.1021/acs.chemmater.4c00979)
Supplement: Supplementary file 1 — cm4c00979_si_001.pdf [file cm4c00979_si_001.pdf]

Supporting Information: Site disorder drives cyanide dynamics and fast ion transport in Li_6PS_5CN

Connor E. Ray,[†] Yi Yao,[‡,¶] Shelby L. Galinat,[†] Bennett Addison,[§] Volker Blum,[‡,¶] and Annalise E. Maughan[*,†,‖]

†*Department of Chemistry, Colorado School of Mines, Golden, Colorado 80401, United States*

‡*Thomas Lord Department of Mechanical Engineering and Materials Science, Duke University, Durham, North Carolina 27708, United States*

¶*Department of Chemistry, Duke University, Durham, North Carolina 27708, United States*

§*Renewable Resources and Enabling Sciences Center, National Renewable Energy Laboratory, Golden, Colorado 80401, United States*

‖*Materials, Chemical, and Computational Science Directorate, National Renewable Energy Laboratory, Golden, Colorado 80401, United States*

E-mail: amaughan@mines.edu

Temperature-Dependent Characterization

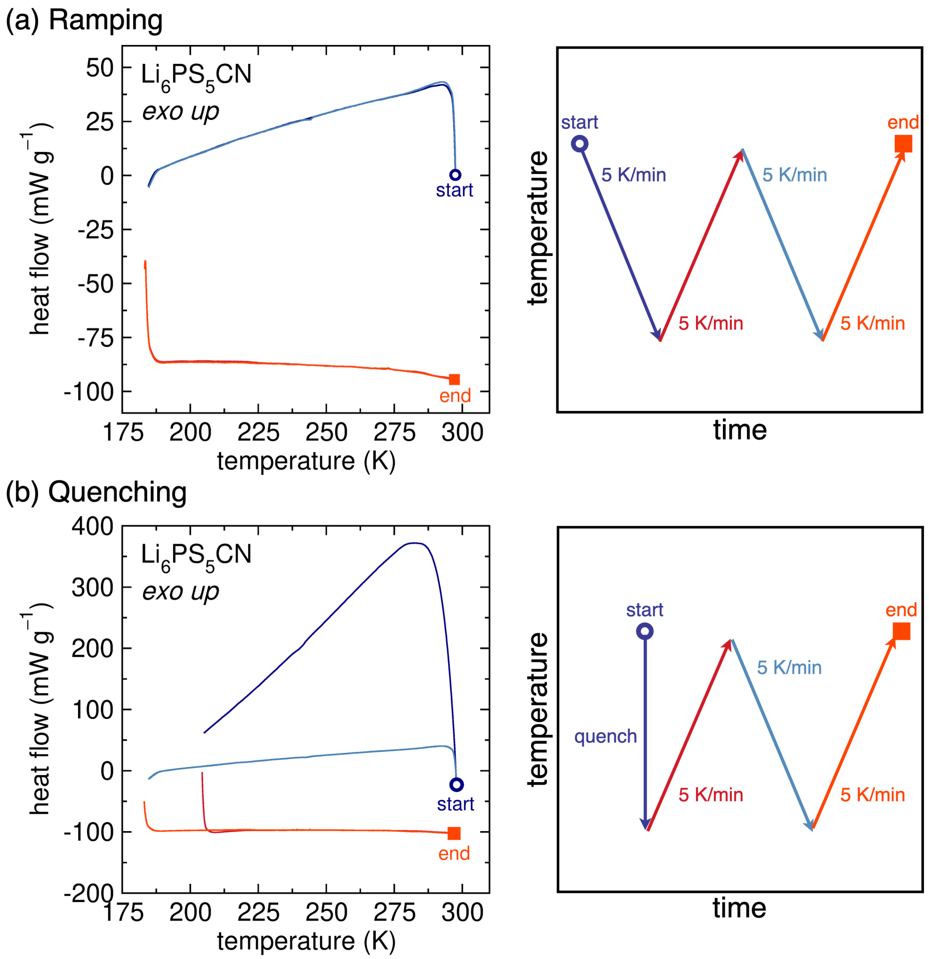

Figure S1: Differential scanning calorimetry (DSC) measurements of Li_6PS_5CN. In (a), measurements were collected under ramping conditions at 5 K/min. In (b), the sample was rapidly quenched to the lowest temperature and then ramped at 5 K/min. The start and end points of the scans are denoted by a blue open circle and filled orange square, respectively. The panels to the right represent the scan parameters for the slow ramped and quenched scans.

^{7}Li Solid-State NMR

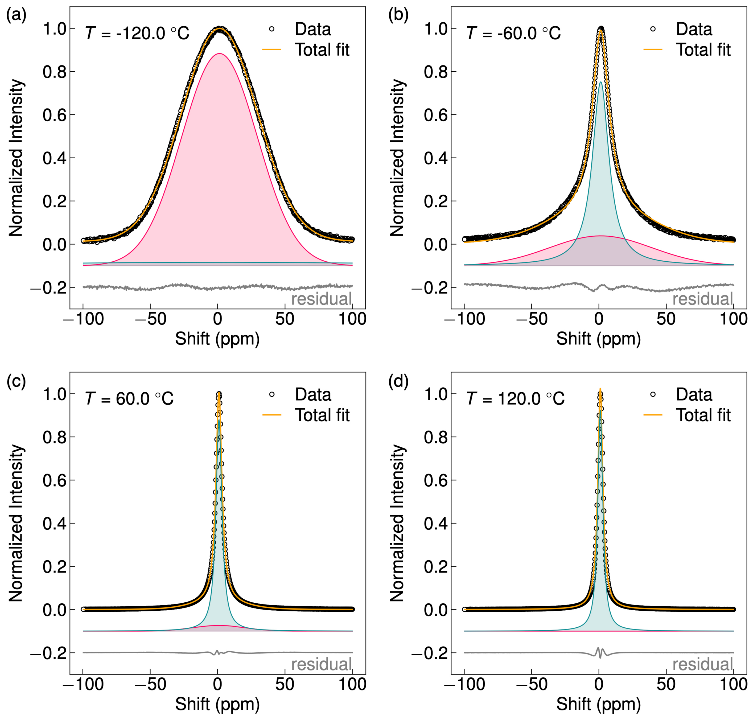

Figure S2: Deconvolution of the static linewidth NMR peaks for Li_6PS_5CN at (a) −120 °C, (b) −60 °C, (c) 60 °C, and (d) 120 °C. Data are shown as open circles and the total peak fit is shown as the orange line. The Gaussian and Lorentzian contributions to the fits are shown as shaded pink and teal curves, respectively, and are offset for clarity. These temperatures were chosen to exemplify peaks in fully Gaussian (a), intermediate (b and c), and fully Lorentzian (d) regimes.

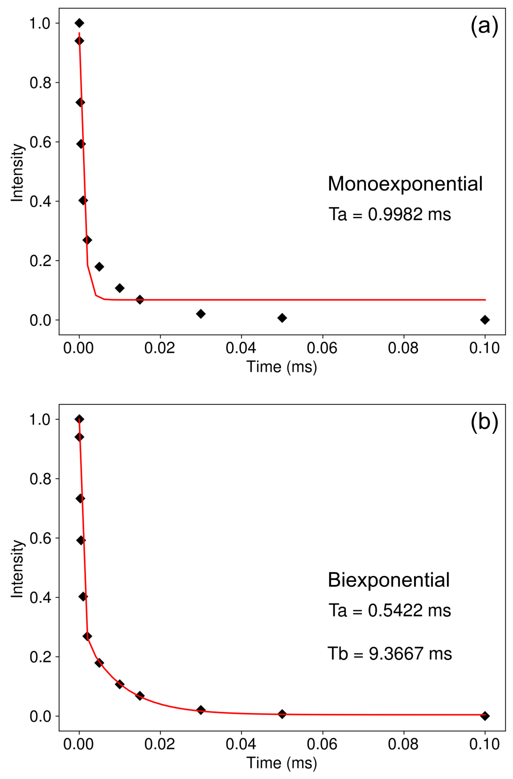

Figure S3: Spin-lattice relaxation NMR decay intensity fitted to a single monoexponential decay function, and a biexponential decay function both shown at 303 K.

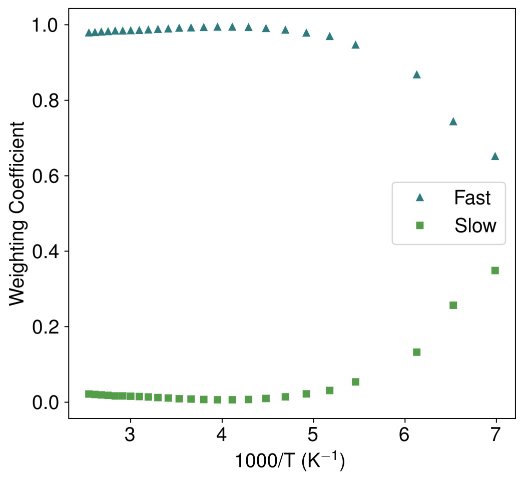

Figure S4: Weights of the exponential decay coefficients for the biexponential $T_{1\rho}$ decay. At high temperatures, the "fast" coefficient dominates and the behavior resembles a monoexponential decay function.

Electrochemical Impedance Spectroscopy

Table S1: Results of temperature-dependent electrochemical impedance spectroscopy measurements of Li_6PS_5CN. Data were fit to the equivalent circuit model ($R_1Q_1 + Q_2$) at all temperatures. Sample thickness = 0.2245(1) cm, sample diameter = 0.6 cm. R is the resistance, Q is the constant phase element, n is the constant phase, and C is the capacitance.

T (°C)	R_1 (Ohms)	Q_1 (S s^{-n})	n_1	σ_1 (S cm^{-1})	C_1 (F)	Q_2 (S s^{-n})	n_2	τ_1 (Hz)
30	1.16E+04	6.50E-10	0.724	6.82E-05	7.22E-12	2.01E-07	6.26E-01	1.89E+06
35	8.46E+03	7.00E-10	0.724	9.39E-05	7.11E-12	1.87E-07	6.43E-01	2.65E+06
45	4.53E+03	1.00E-09	0.716	1.75E-04	7.55E-12	2.38E-07	6.58E-01	4.65E+06
55	2.83E+03	8.77E-10	0.742	2.80E-04	9.95E-12	2.90E-07	6.71E-01	5.65E+06
65	1.73E+03	7.71E-10	0.776	4.60E-04	1.55E-11	3.56E-07	6.88E-01	5.95E+06
75	1.15E+03	9.92E-10	0.783	6.88E-04	2.25E-11	4.34E-07	7.01E-01	6.13E+06
85	8.18E+02	9.91E-10	0.798	9.71E-04	2.86E-11	5.70E-07	7.06E-01	6.81E+06
95	5.53E+02	9.95E-10	0.811	1.43E-03	3.47E-11	8.56E-07	7.05E-01	8.29E+06

Machine Learning-Assisted Molecular Dynamics Simulations

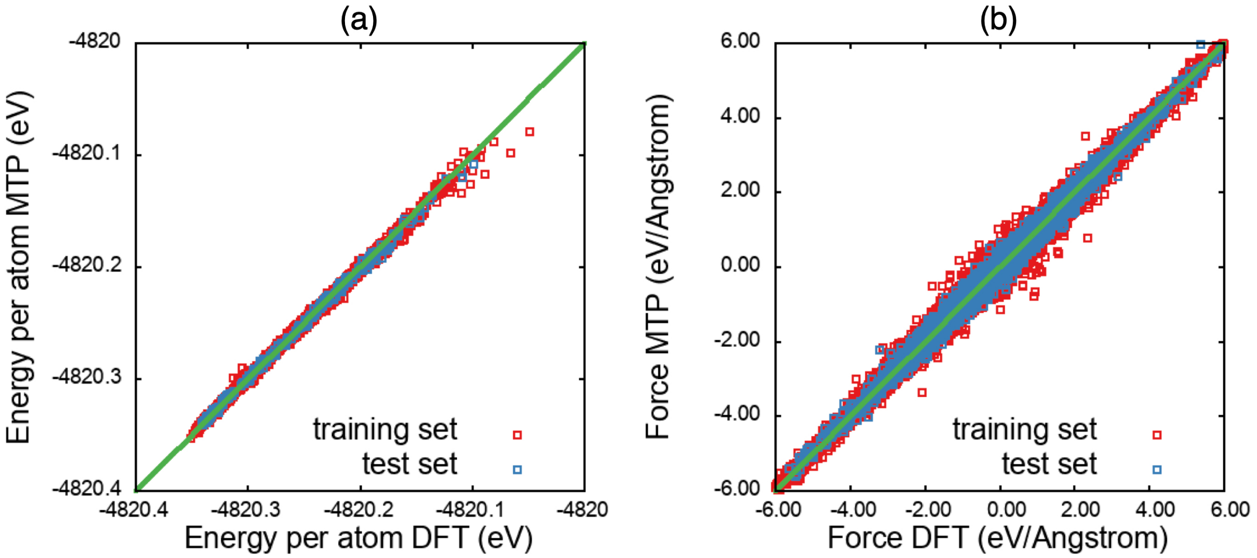

Figure S5: The scatter plots for the (a) potential energies and (b) forces calculated by direct first principles calculation and MTP model for Li_6PS_5CN at the PBE+MBD levels of theory. The plots include total of 2160 training configurations and 240 test configurations. The green diagonal line indicates perfect agreement.

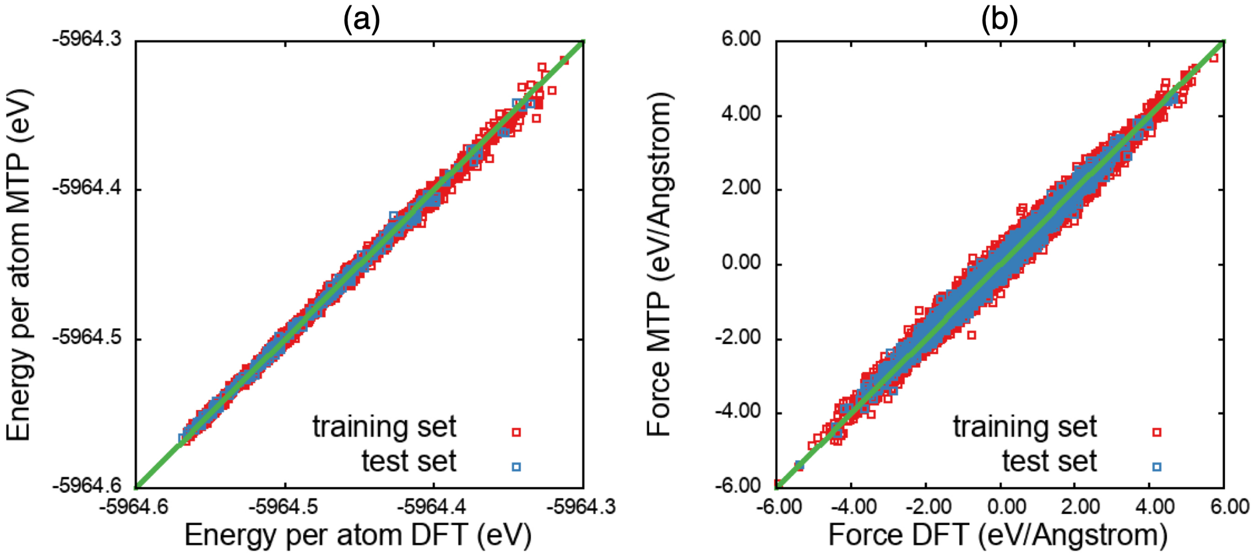

Figure S6: The scatter plots for the (a) potential energies and (b) forces calculated by direct first principles calculation and MTP model for Li_6PS_5Cl at the PBE+MBD levels of theory. The plots include total of 2160 training configurations and 240 test configurations. The green diagonal line indicates perfect agreement.

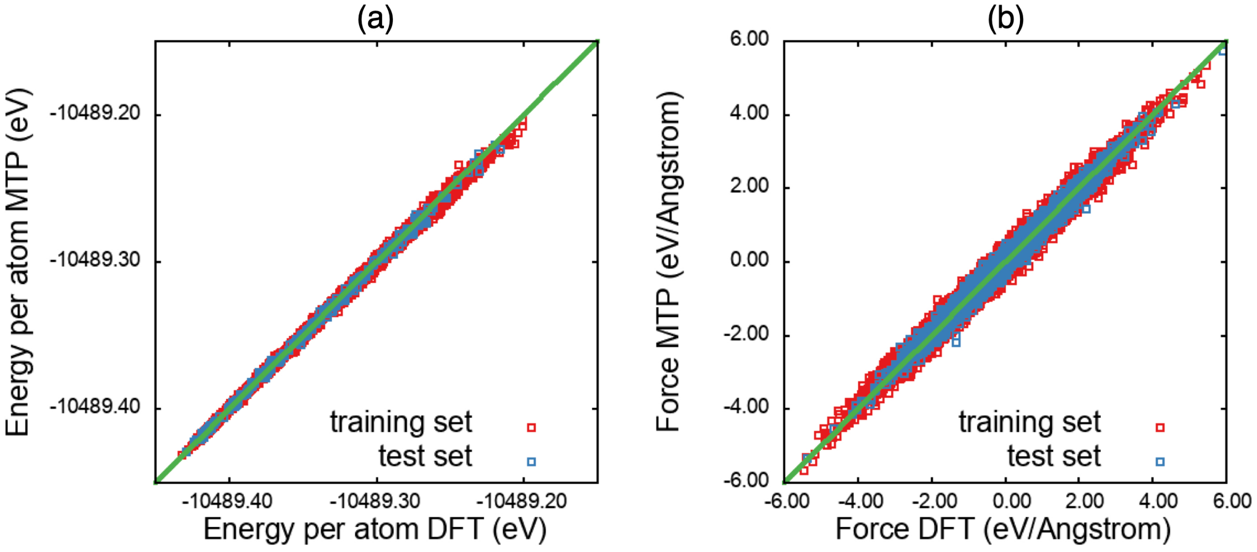

Figure S7: The scatter plots for the (a) potential energies and (b) forces calculated by direct first principles calculation and MTP model for Li_6PS_5Br at the PBE+MBD levels of theory. The plots include total of 2160 training configurations and 240 test configurations. The green diagonal line indicates perfect agreement.

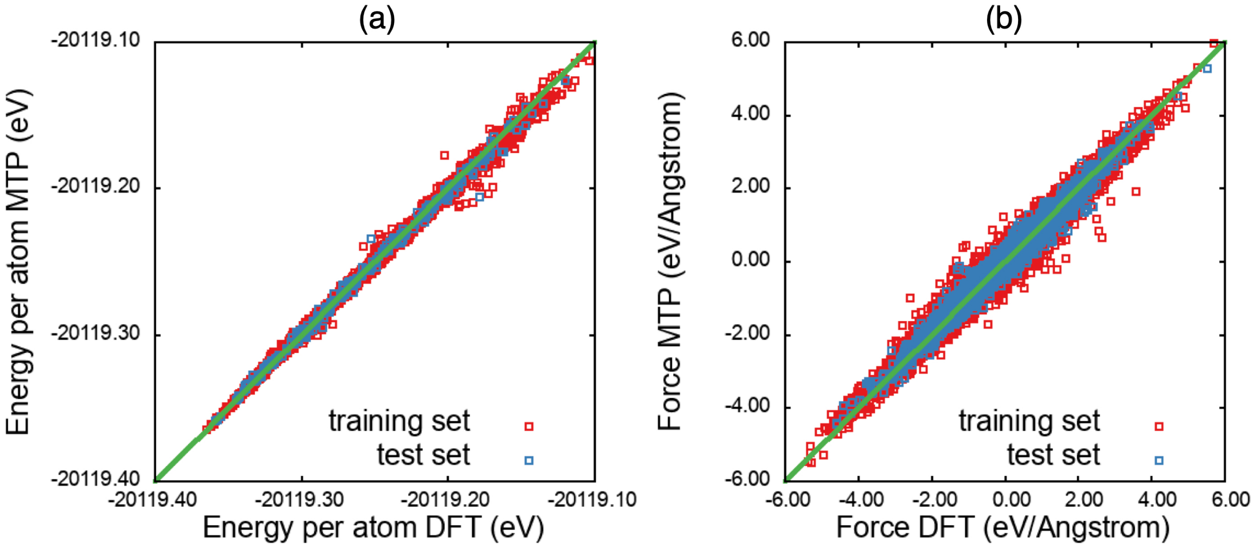

Figure S8: The scatter plots for the (a) potential energies and (b) forces calculated by direct first principles calculation and MTP model for Li_6PS_5I at the PBE+MBD levels of theory. The plots include total of 2160 training configurations and 240 test configurations. The green diagonal line indicates perfect agreement.

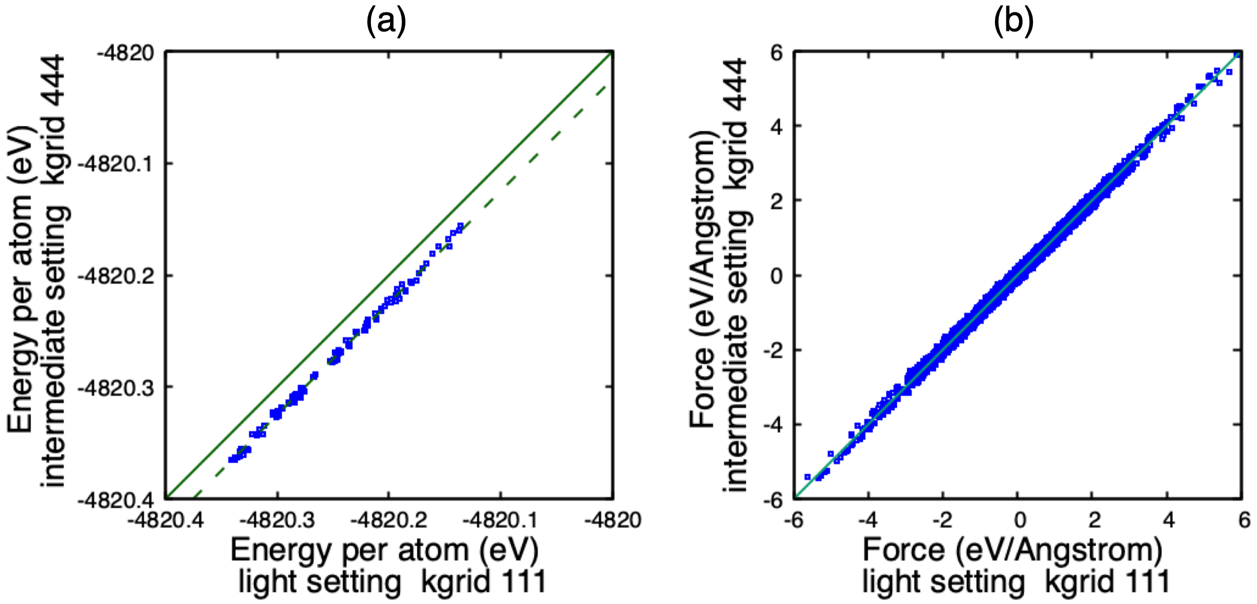

Figure S9: The scatter plots for the (a) potential energies and (b) forces calculated by light setting with k grid 111 and intermediate setting with k grid 444 for Li_6PS_5CN. The plots include 100 snapshots from the simulations taken from the MD trajectories. The green diagonal line indicates perfect agreement. The dashed green line in the potential energies plot indicates a constant offset.

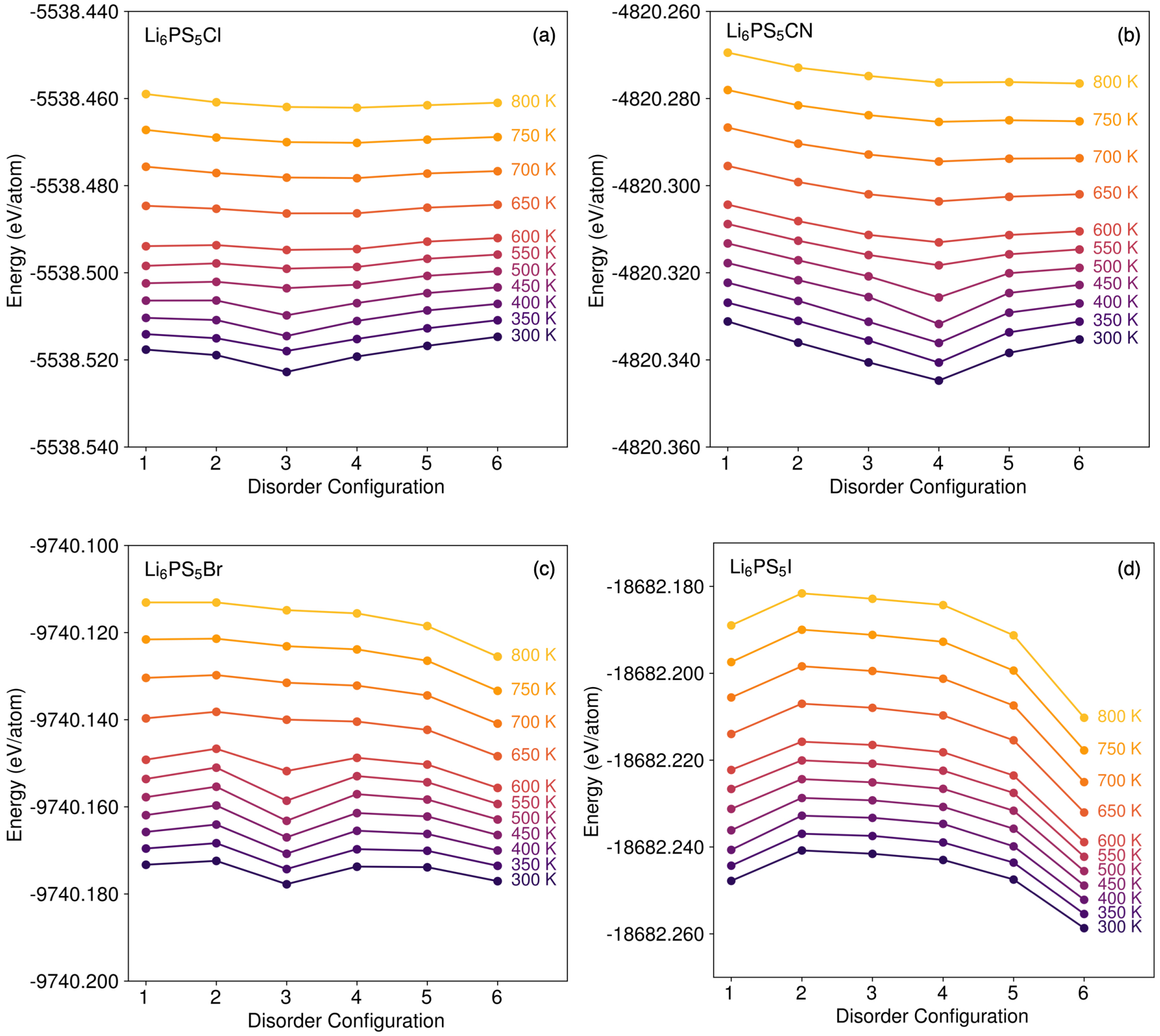

Figure S10: The potential energy (eV/atom) for Li_6PS_5X (X = Cl^- (a), CN^- (b), Br^- (c), or I^- (d) argyrodite structures calculated by averaging the potential energies of the machine learning assisted *ab initio* molecular dynamics within NPT ensemble. The energies were calculated for each disorder configuration at varying temperatures, as denoted by the colored traces.

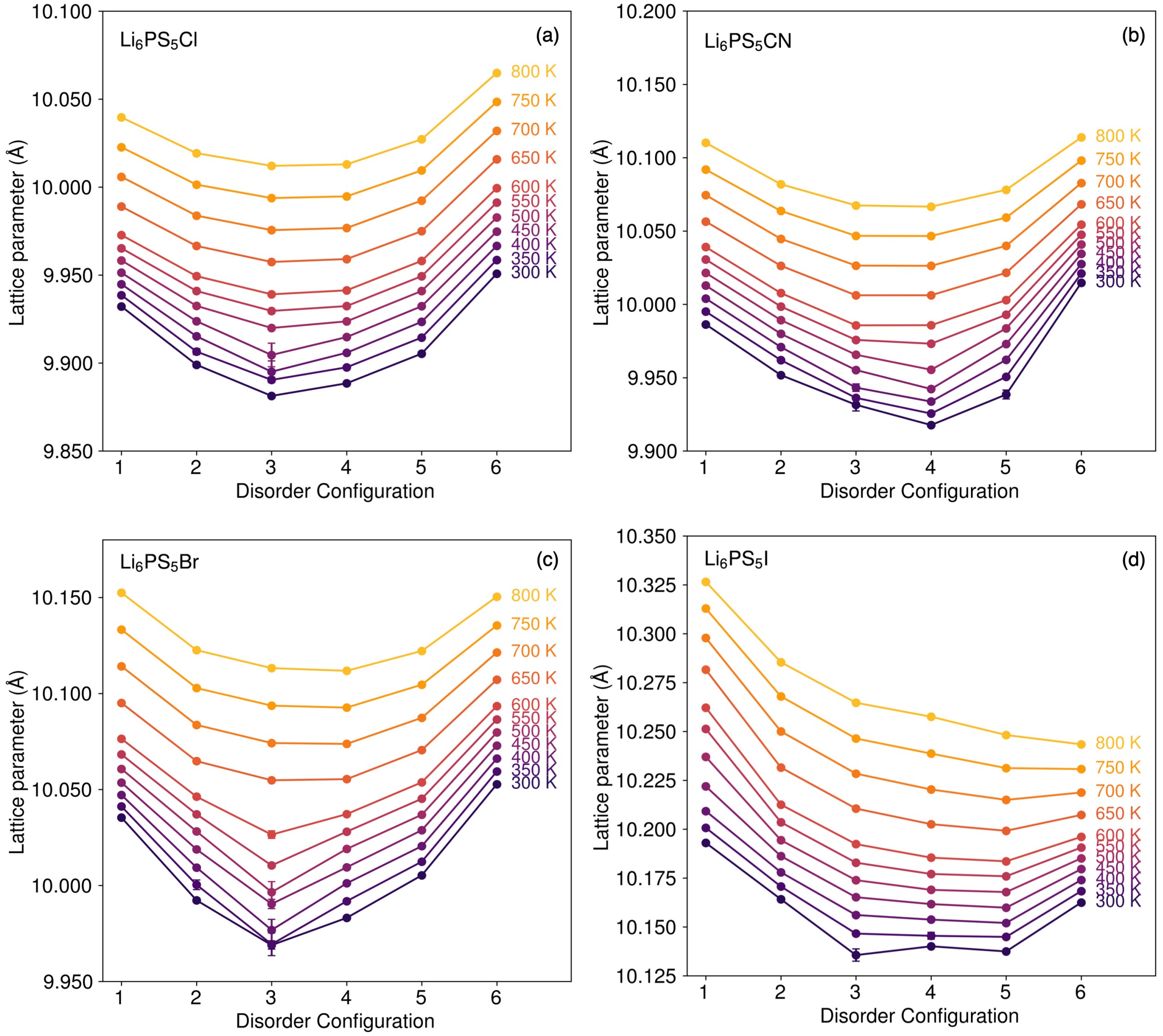

Figure S11: The lattice parameters for Li_6PS_5X (X = Cl^- (a), CN^- (b), Br^- (c), or I^- (d) argyrodite structures calculated by averaging the machine learning-assisted molecular dynamics within NPT ensemble. The lattice parameters were calculated for each disorder configuration at varying temperatures, as denoted by the colored traces.

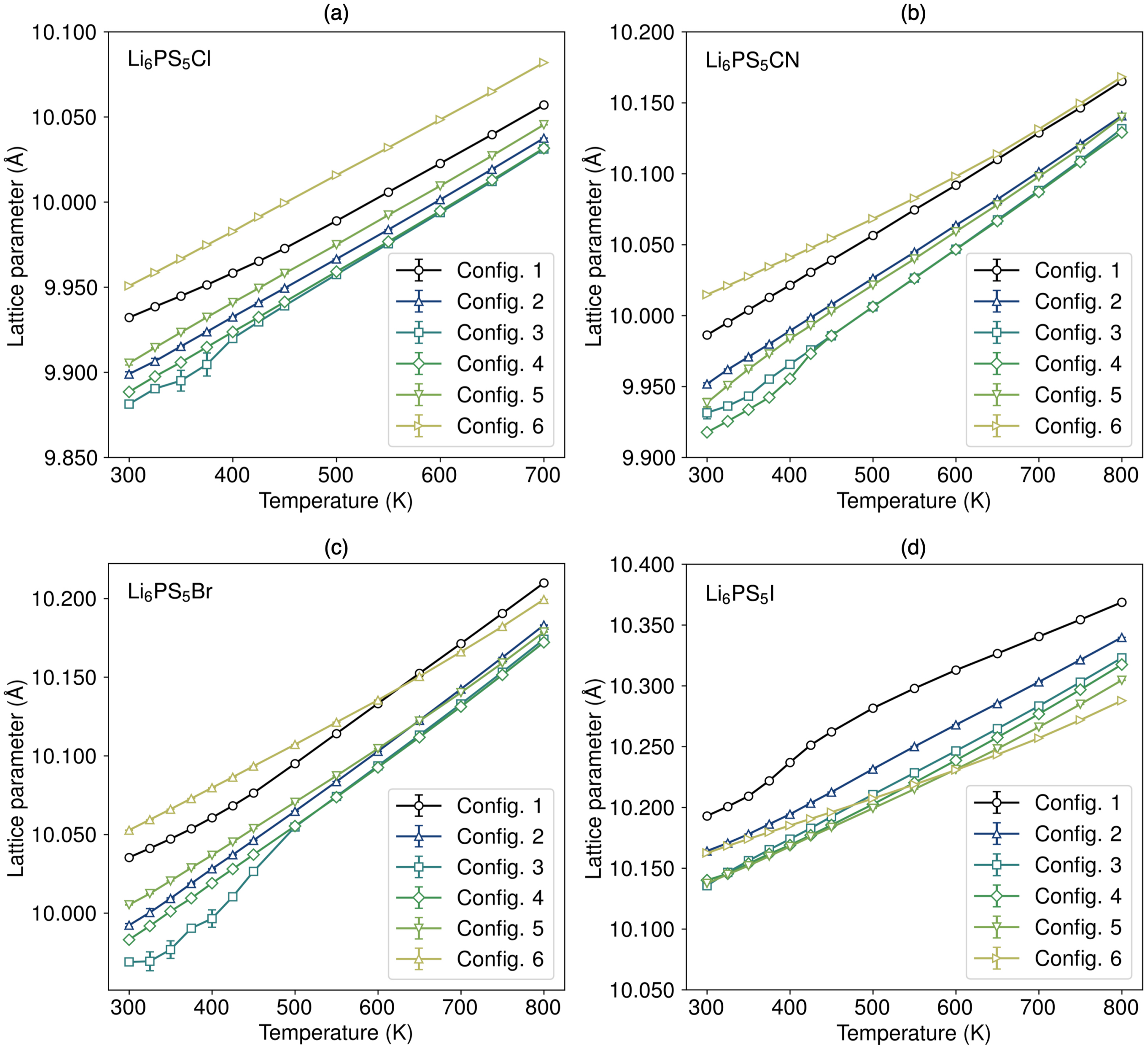

Figure S12: The lattice parameters as a function of temperature for Li_6PS_5X (X = Cl^-(a), CN^- (b), Br^- (c), or I^- (d) argyrodite structures calculated by averaging the machine learning assisted molecular dynamics within NPT ensemble. The lattice parameters were calculated for each disorder configuration as denoted by the colored traces.

Lithium self-diffusion coefficients (D_{Li}) were calculated from the slope of the Li MSD according to the Einstein relation

$$D_{\mathrm{Li}} = \lim_{t\to\infty} \frac{\langle|\delta r_{\mathrm{Li}}(t)|\rangle^2}{6t} \tag{1}$$

where $\delta r_{\mathrm{Li}}(t)$ is the displacement of Li^+ at time t.[1,2] The slope of the linear regime of the Li^+

MSD was determined from linear regression between $t = 400\,\text{ps}$ to $t = 1200\,\text{ps}$ for each halide (X = Cl^-, CN^-, Br^-, I^-), each temperature, and for each Configuration of anion order from MD simulations. At higher temperatures ($T \geq 500$ K), the Li^+ diffusion coefficients approximate a linear relationship in $\ln(D_{\text{Li}})$ v. 1000/T, which suggests Arrhenius-like behavior. We note that simulations performed at lower temperatures ($T < 500$ K) are often not fully converged between $t = 400\,\text{ps}$ to $t = 1200\,\text{ps}$ (e.g., the plot of mean-squared displacement vs. temperature is not linear) for structures with low lithium self-diffusion, as shown in Figures S13-S16 and evidenced by deviations from linearity in plots of $\ln(D_{\text{Li}})$ v. 1000/T. To provide a more accurate estimation of the Li^+ diffusion coefficients at lower temperatures, we fit an Arrhenius relationship from $T = 500 - 800$ K and extrapolated the lithium ion diffusion coefficients for $T = 300 - 450$ K. The results are tabulated in Tables S2-S5.

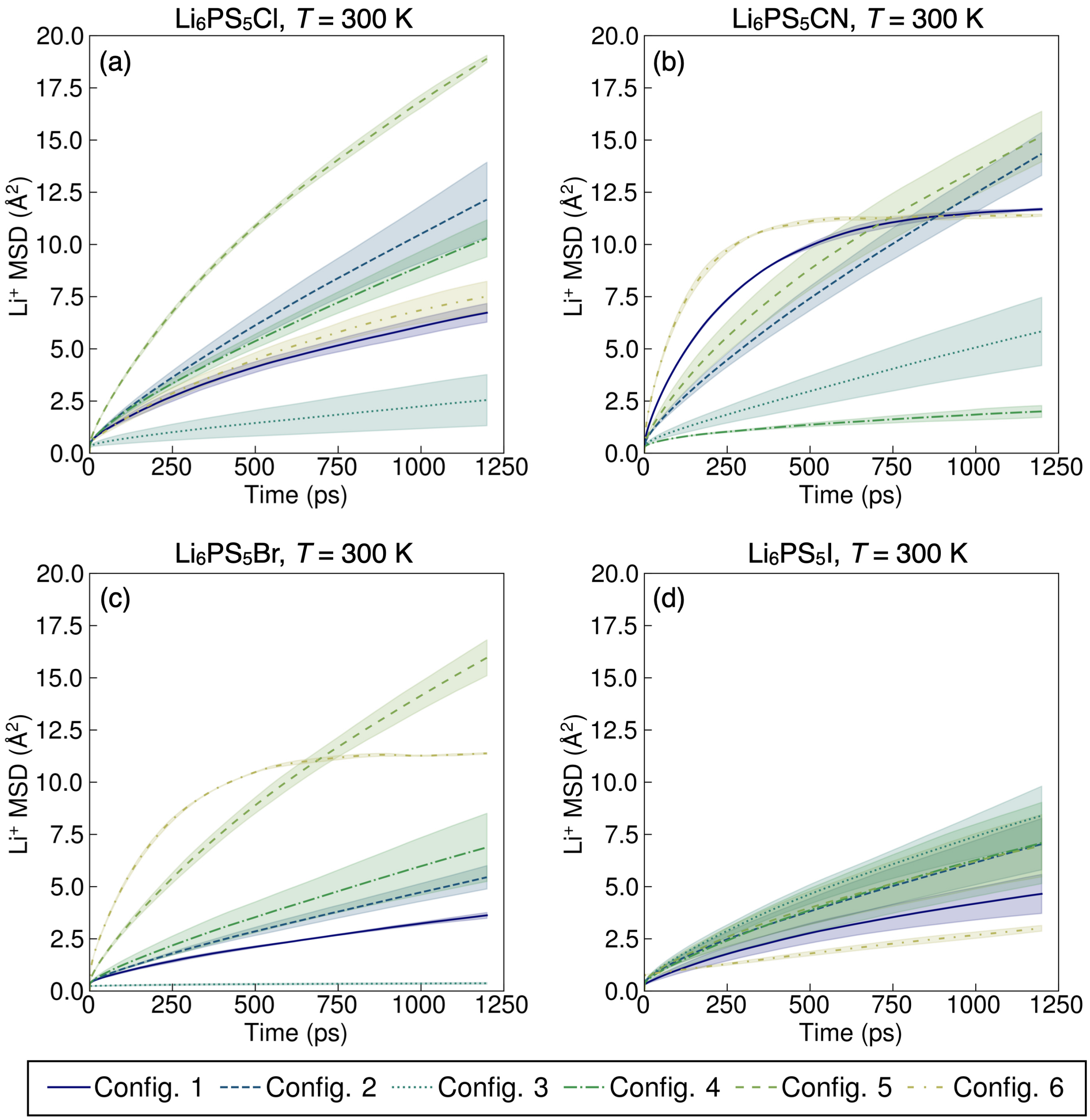

Figure S13: Lithium ion mean squared displacement for each of the six Configurations for the (a) chloride, (b) cyanide, (c) bromide, and (d) iodide argyrodite structures from molecular dynamics simulations. Simulations were performed at T = 300 K in triplicate. Lines represent the average MSD from the three runs and the shaded areas represent the standard deviations between replicate runs.

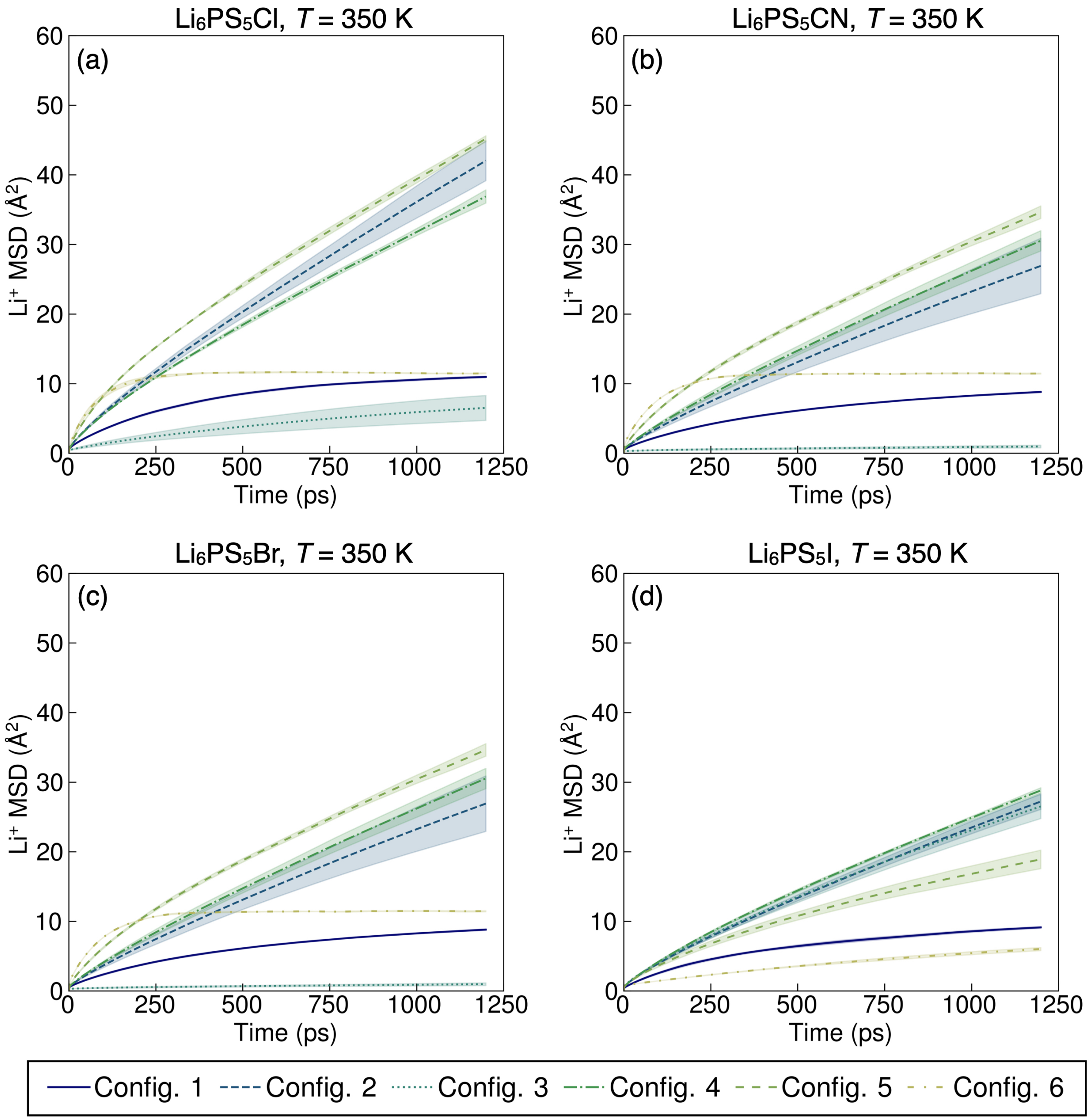

Figure S14: Lithium ion mean squared displacement for each of the six Configurations for the (a) chloride, (b) cyanide, (c) bromide, and (d) iodide argyrodite structures from molecular dynamics simulations. Simulations were performed at T = 350 K in triplicate. Lines represent the average MSD from the three runs and the shaded areas represent the standard deviations between replicate runs.

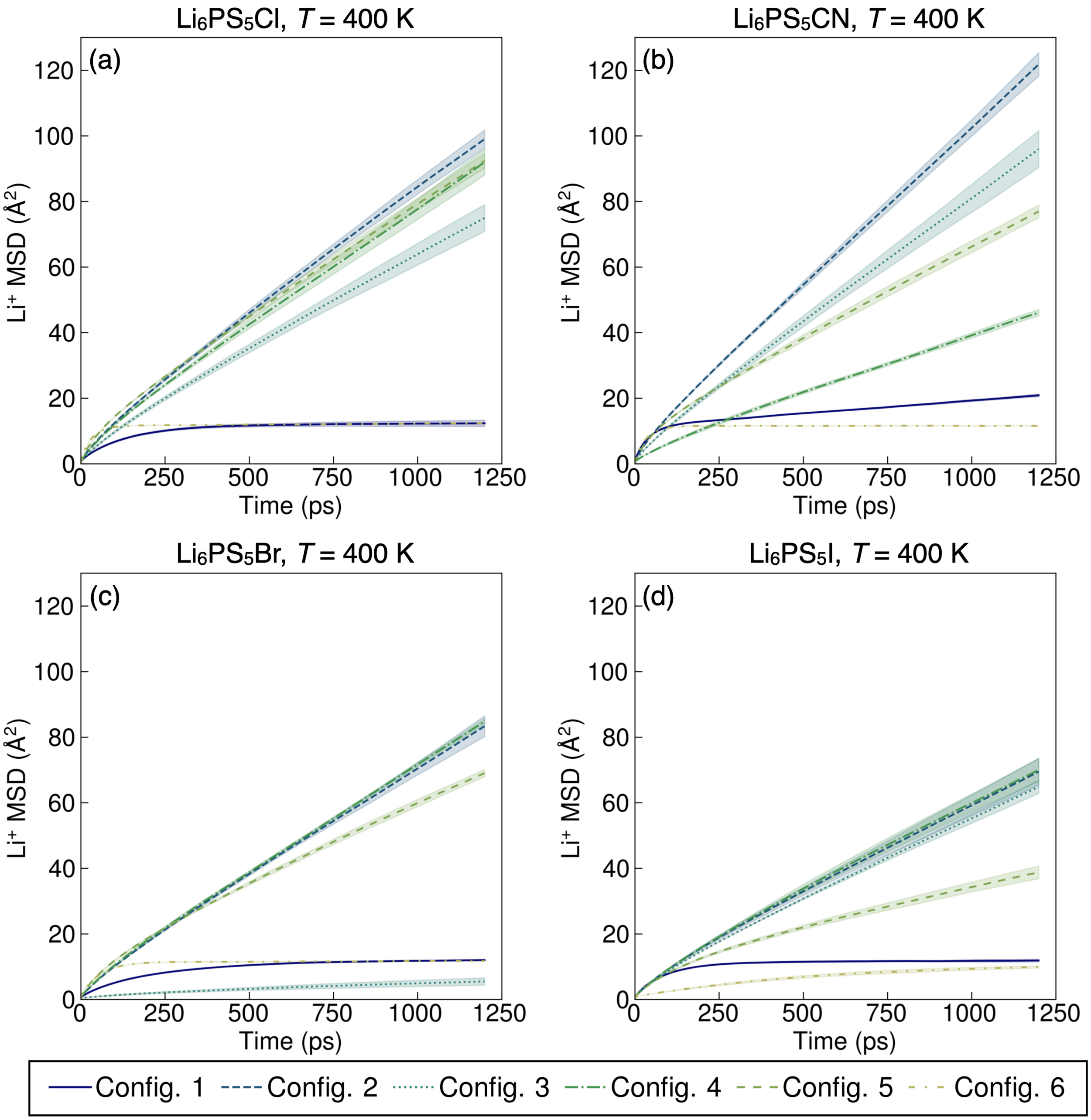

Figure S15: Lithium ion mean squared displacement for each of the six Configurations for the (a) chloride, (b) cyanide, (c) bromide, and (d) iodide argyrodite structures from molecular dynamics simulations. Simulations were performed at $T = 400\,\mathrm{K}$ in triplicate. Lines represent the average MSD from the three runs and the shaded areas represent the standard deviations between replicate runs.

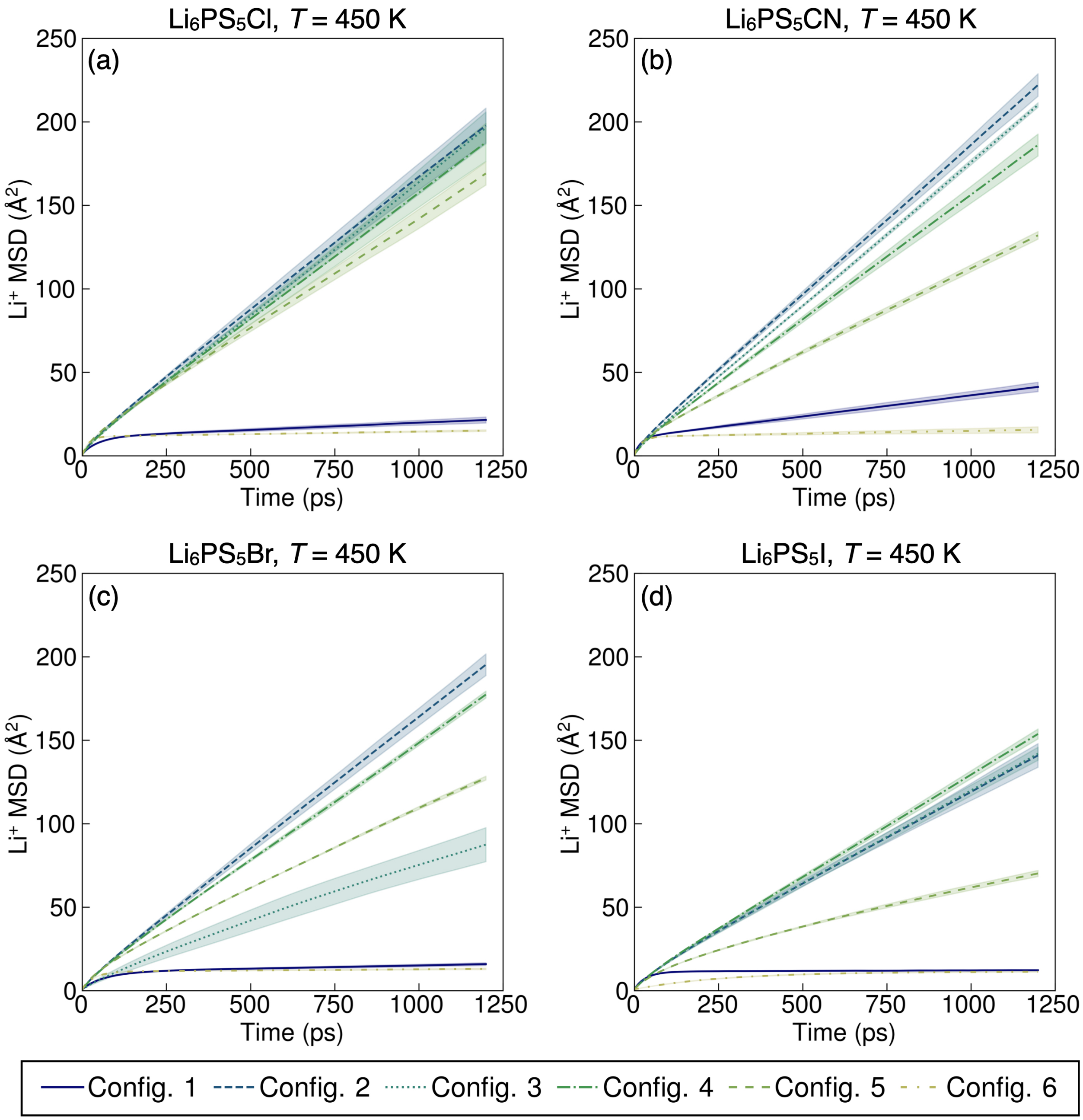

Figure S16: Lithium ion mean squared displacement for each of the six Configurations for the (a) chloride, (b) cyanide, (c) bromide, and (d) iodide argyrodite structures from molecular dynamics simulations. Simulations were performed at $T = 450\,\mathrm{K}$ in triplicate. Lines represent the average MSD from the three runs and the shaded areas represent the standard deviations between replicate runs.

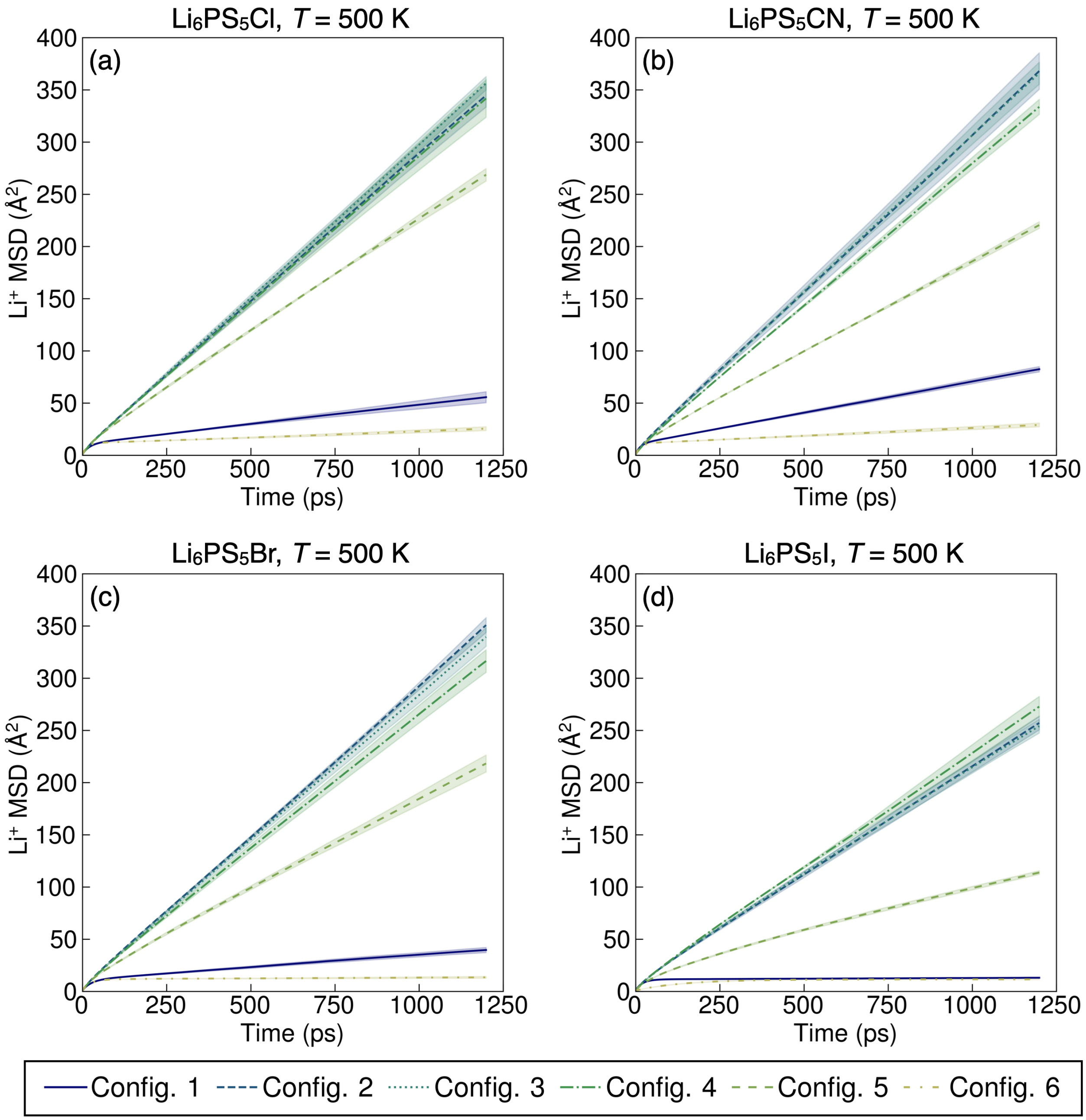

Figure S17: Lithium ion mean squared displacement for each of the six Configurations for the (a) chloride, (b) cyanide, (c) bromide, and (d) iodide argyrodite structures from molecular dynamics simulations. Simulations were performed at $T = 500\,\mathrm{K}$ in triplicate. Lines represent the average MSD from the three runs and the shaded areas represent the standard deviations between replicate runs.

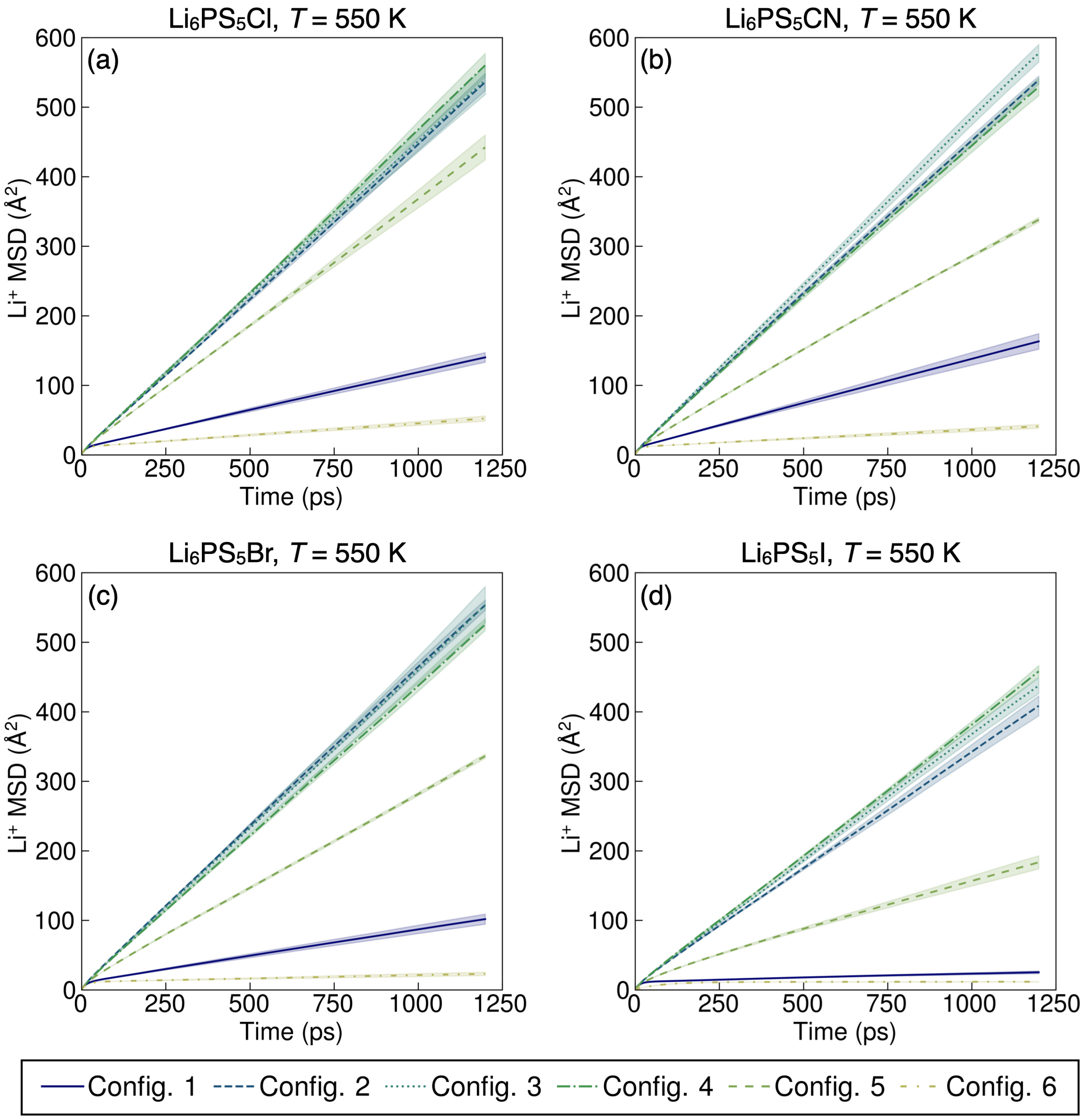

Figure S18: Lithium ion mean squared displacement for each of the six Configurations for the (a) chloride, (b) cyanide, (c) bromide, and (d) iodide argyrodite structures from molecular dynamics simulations. Simulations were performed at T = 550 K in triplicate. Lines represent the average MSD from the three runs and the shaded areas represent the standard deviations between replicate runs.

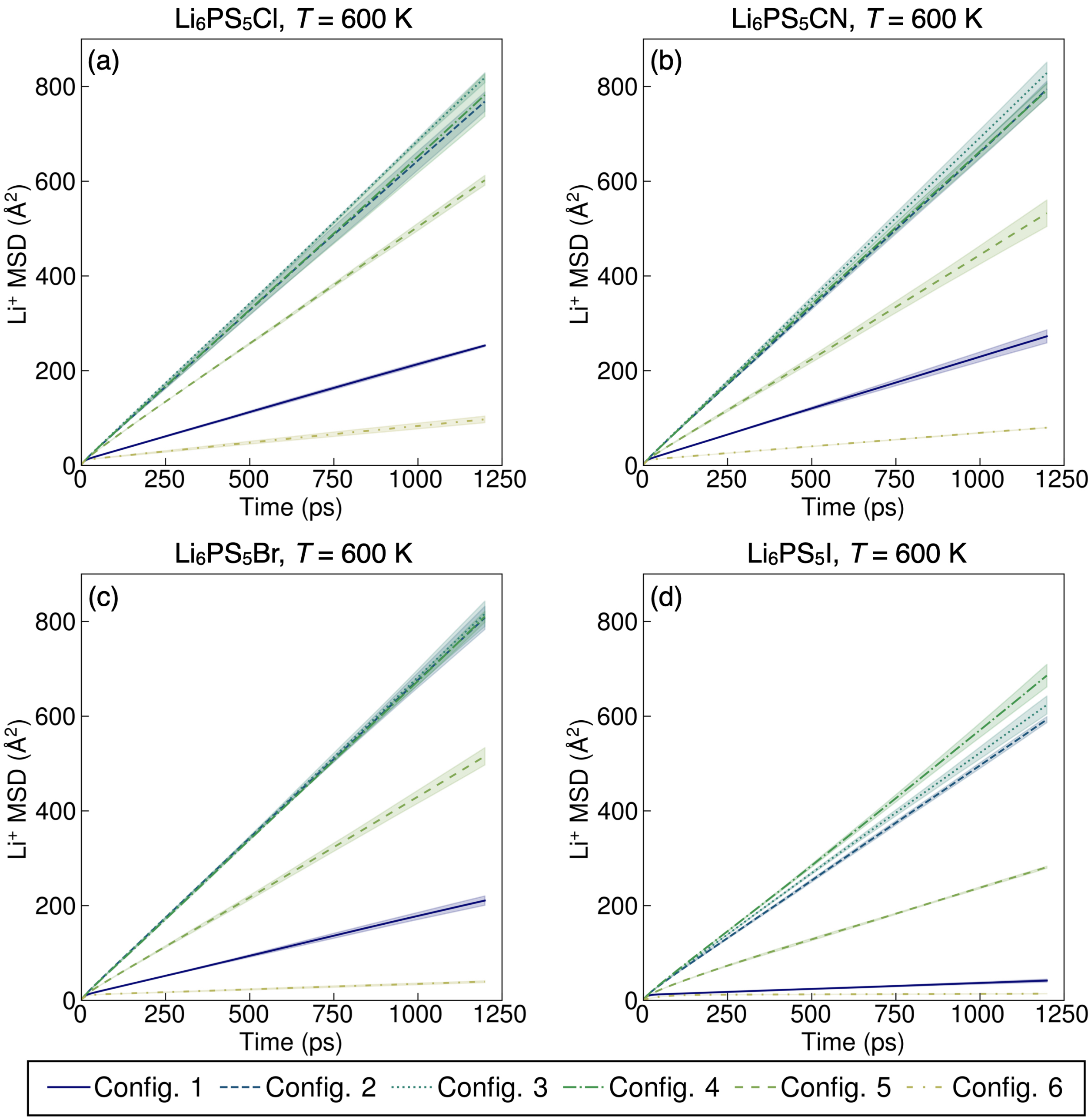

Figure S19: Lithium ion mean squared displacement for each of the six Configurations for the (a) chloride, (b) cyanide, (c) bromide, and (d) iodide argyrodite structures from molecular dynamics simulations. Simulations were performed at T = 600 K in triplicate. Lines represent the average MSD from the three runs and the shaded areas represent the standard deviations between replicate runs.

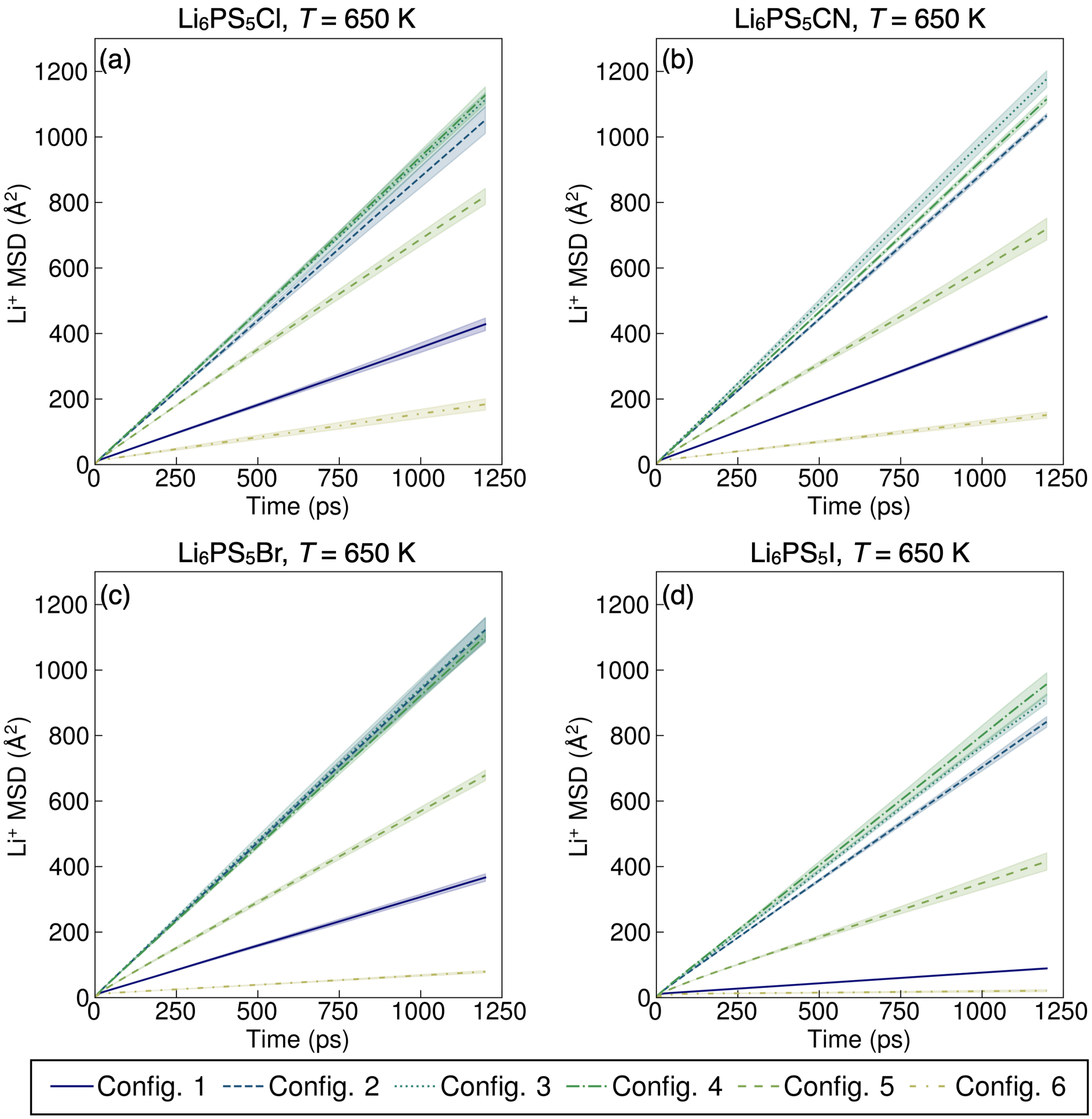

Figure S20: Lithium ion mean squared displacement for each of the six Configurations for the (a) chloride, (b) cyanide, (c) bromide, and (d) iodide argyrodite structures from molecular dynamics simulations. Simulations were performed at $T = 650\,\text{K}$ in triplicate. Lines represent the average MSD from the three runs and the shaded areas represent the standard deviations between replicate runs.

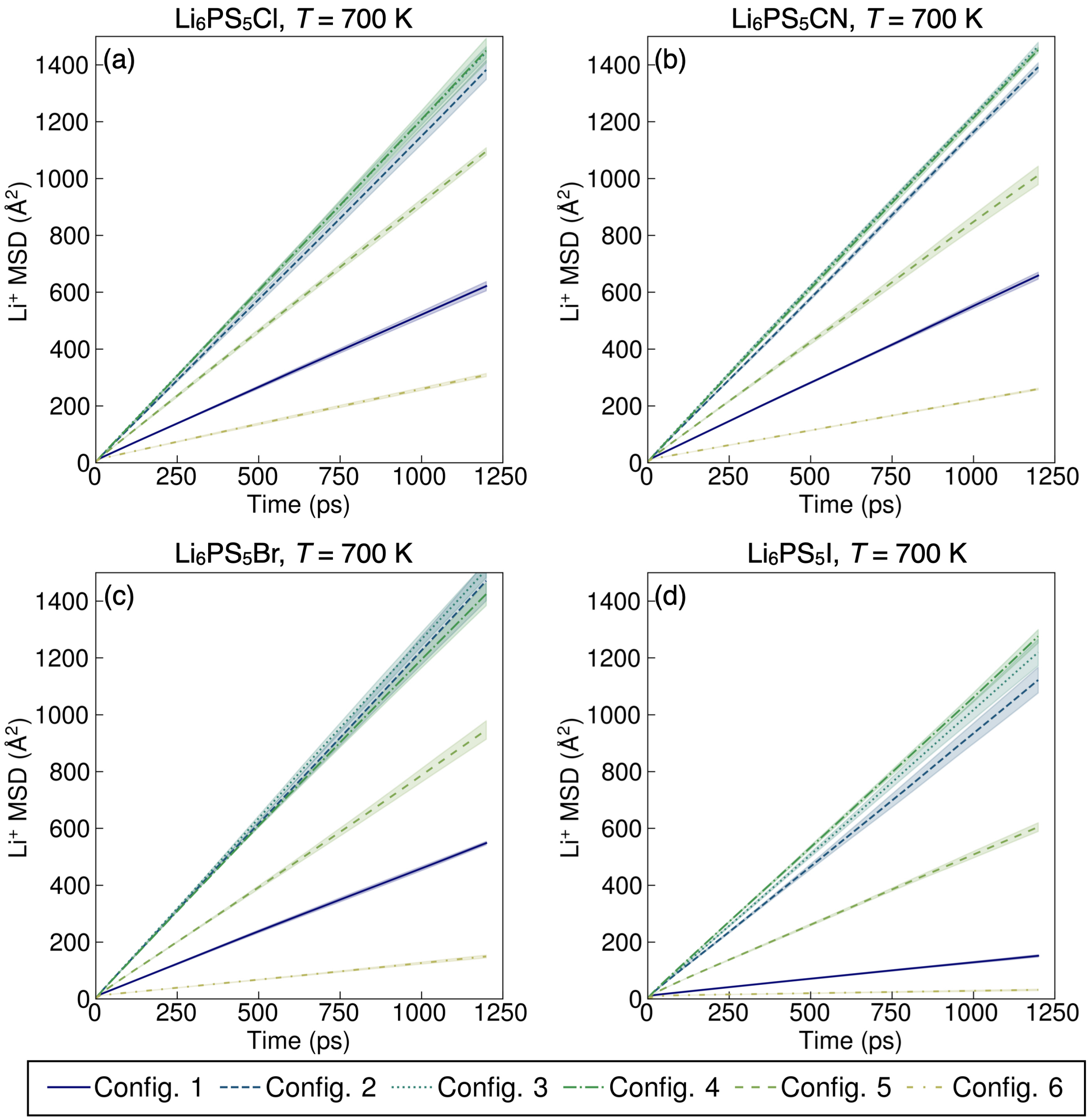

Figure S21: Lithium ion mean squared displacement for each of the six Configurations for the (a) chloride, (b) cyanide, (c) bromide, and (d) iodide argyrodite structures from molecular dynamics simulations. Simulations were performed at T = 700 K in triplicate. Lines represent the average MSD from the three runs and the shaded areas represent the standard deviations between replicate runs.

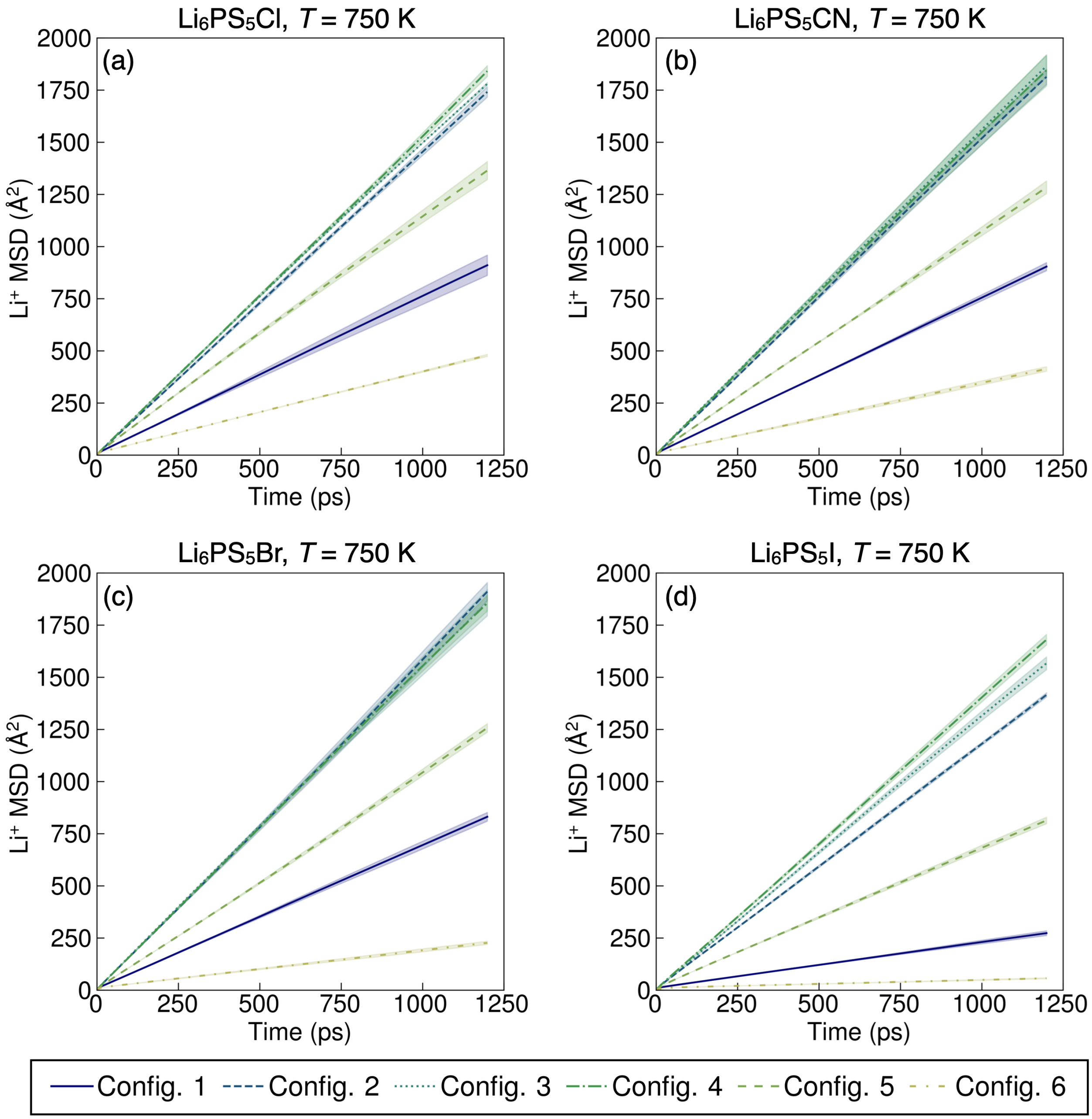

Figure S22: Lithium ion mean squared displacement for each of the six Configurations for the (a) chloride, (b) cyanide, (c) bromide, and (d) iodide argyrodite structures from molecular dynamics simulations. Simulations were performed at T = 750 K in triplicate. Lines represent the average MSD from the three runs and the shaded areas represent the standard deviations between replicate runs.

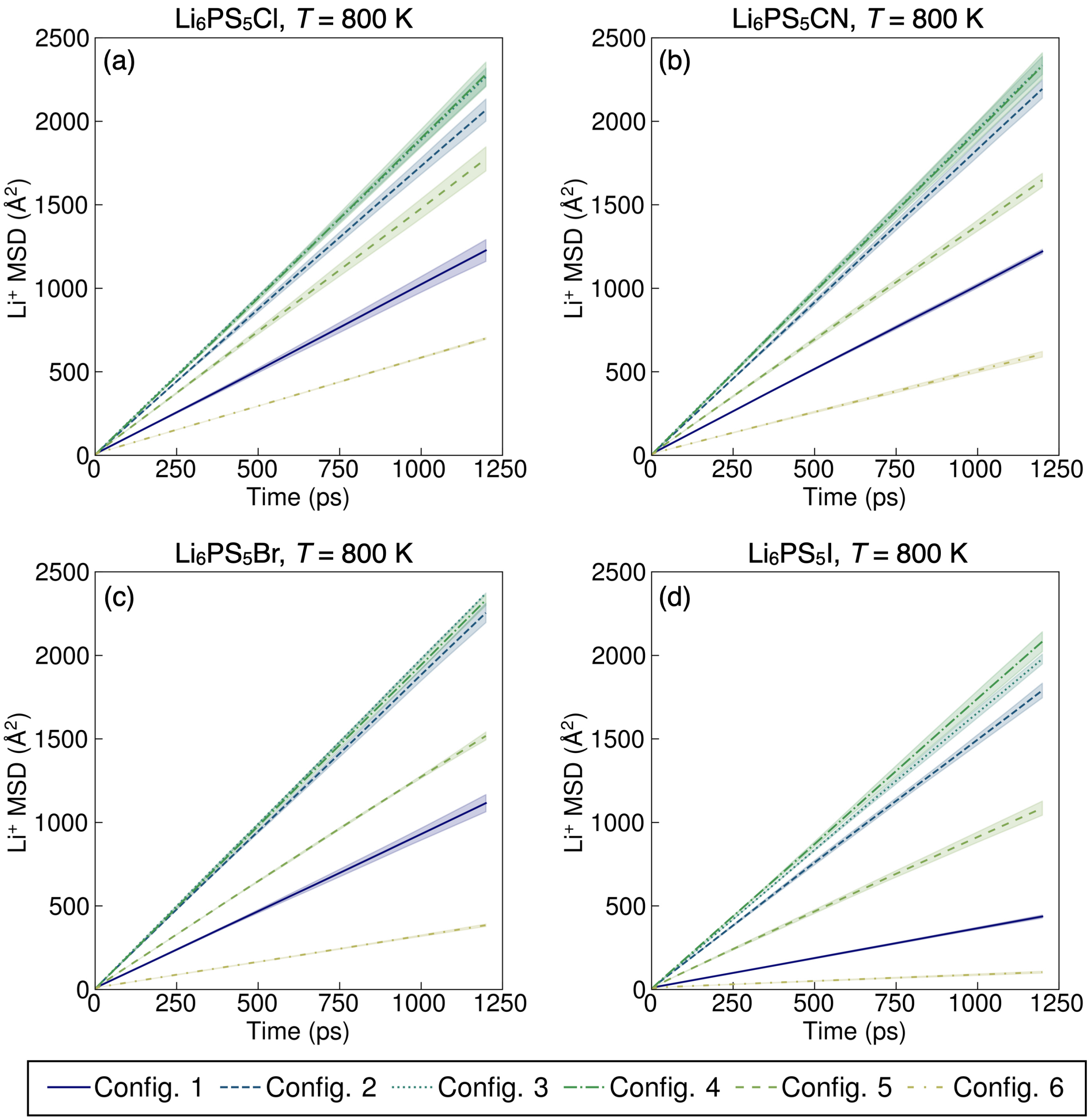

Figure S23: Lithium ion mean squared displacement for each of the six Configurations for the (a) chloride, (b) cyanide, (c) bromide, and (d) iodide argyrodite structures from molecular dynamics simulations. Simulations were performed at T = 800 K in triplicate. Lines represent the average MSD from the three runs and the shaded areas represent the standard deviations between replicate runs.

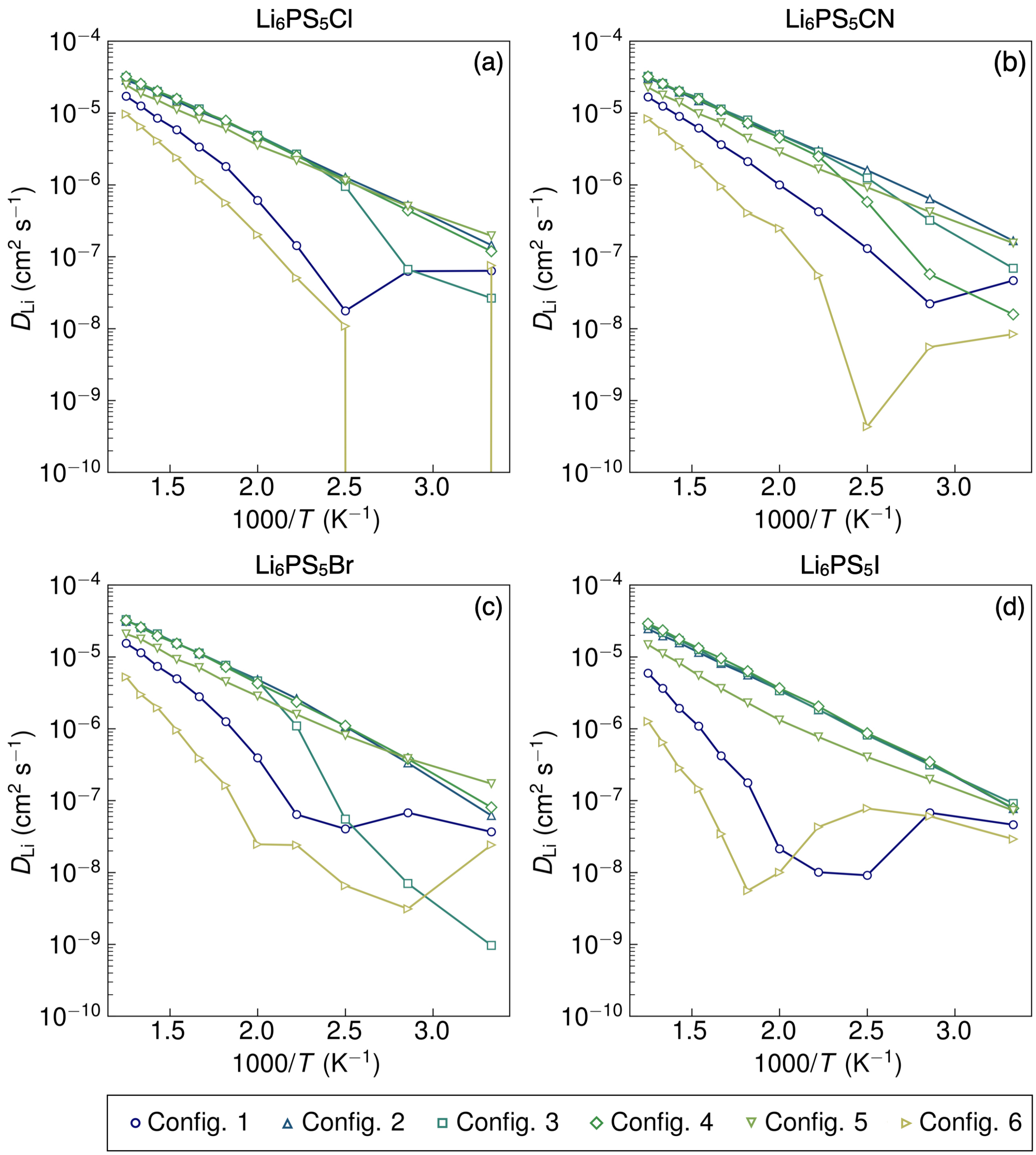

Figure S24: Arrhenius plots for (a) Li_6PS_5Cl, (b) Li_6PS_5CN, (c) Li_6PS_5Br, and (d) Li_6PS_5I. Lithium diffusion coefficients (D_{Li}) were determined from the slope of the Li^+ mean-squared displacements from molecular dynamics simulations. The different marker styles correspond to the different configurations of site mixing.

Table S2: Lithium diffusion coefficients (D_{Li}) for Li_6PS_5Cl for each disorder configuration determined from machine learning-assisted molecular dynamics simulations. Values are listed in units of $cm^2\ s^{-1}$. For $T < 500$ K, diffusion coefficients were determined both from the slope of the Li^+ MSD and also extrapolated from linear fits in the Arrhenius plot at higher simulation temperatures ($T = 500$ K to $T = 800$ K) for comparison.

T (K)	Config. 1	Config. 2	Config. 3	Config. 4	Config. 5	Config. 6
Extrapolated from Arrhenius						
300	2.256×10^{-9}	1.890×10^{-7}	1.804×10^{-7}	1.616×10^{-7}	1.305×10^{-7}	2.232×10^{-10}
350	1.772×10^{-8}	5.978×10^{-7}	5.851×10^{-7}	5.409×10^{-7}	4.282×10^{-7}	2.585×10^{-9}
400	8.318×10^{-8}	1.418×10^{-6}	1.414×10^{-6}	1.338×10^{-6}	1.044×10^{-6}	1.623×10^{-8}
450	2.769×10^{-7}	2.776×10^{-6}	2.808×10^{-6}	2.707×10^{-6}	2.088×10^{-6}	6.775×10^{-8}
Extracted from Li^+ MSD						
300	6.37×10^{-8}	1.45×10^{-7}	2.65×10^{-8}	1.19×10^{-7}	1.95×10^{-7}	7.55×10^{-8}
350	6.28×10^{-8}	5.22×10^{-7}	6.68×10^{-8}	4.42×10^{-7}	5.05×10^{-7}	-3.50×10^{-9}
400	1.76×10^{-8}	1.27×10^{-6}	9.52×10^{-7}	1.18×10^{-6}	1.14×10^{-6}	1.08×10^{-8}
450	1.43×10^{-7}	2.64×10^{-6}	2.68×10^{-6}	2.52×10^{-6}	2.19×10^{-6}	5.07×10^{-8}
500	6.10×10^{-7}	4.71×10^{-6}	4.89×10^{-6}	4.67×10^{-6}	3.55×10^{-6}	2.02×10^{-7}
550	1.80×10^{-6}	7.45×10^{-6}	7.34×10^{-6}	7.81×10^{-6}	6.08×10^{-6}	5.63×10^{-7}
600	3.36×10^{-6}	1.05×10^{-5}	1.14×10^{-5}	1.08×10^{-5}	8.22×10^{-6}	1.17×10^{-6}
650	5.85×10^{-6}	1.46×10^{-5}	1.55×10^{-5}	1.58×10^{-5}	1.12×10^{-5}	2.40×10^{-6}
700	8.47×10^{-6}	1.92×10^{-5}	2.00×10^{-5}	2.01×10^{-5}	1.51×10^{-5}	4.10×10^{-6}
750	1.25×10^{-5}	2.41×10^{-5}	2.44×10^{-5}	2.55×10^{-5}	1.86×10^{-5}	6.48×10^{-6}
800	1.71×10^{-5}	2.85×10^{-5}	3.14×10^{-5}	3.19×10^{-5}	2.46×10^{-5}	9.65×10^{-6}

Table S3: Lithium diffusion coefficients (D_{Li}) for Li_6PS_5CN for each disorder configuration determined from machine learning-assisted molecular dynamics simulations. Values are listed in units of $\mathrm{cm}^2\ \mathrm{s}^{-1}$. For $T < 500$ K, diffusion coefficients were determined both from the slope of the Li^+ MSD and also extrapolated from linear fits in the Arrhenius plot at higher simulation temperatures ($T = 500$ K to $T = 800$ K) for comparison.

T (K)	Config. 1	Config. 2	Config. 3	Config. 4	Config. 5	Config. 6
Extrapolated from Arrhenius						
300	7.021×10^{-9}	1.887×10^{-7}	1.897×10^{-7}	1.395×10^{-7}	6.941×10^{-8}	2.977×10^{-10}
350	4.185×10^{-8}	6.022×10^{-7}	6.124×10^{-7}	4.833×10^{-7}	2.603×10^{-7}	3.068×10^{-9}
400	1.596×10^{-7}	1.438×10^{-6}	1.475×10^{-6}	1.228×10^{-6}	7.016×10^{-7}	1.765×10^{-8}
450	4.522×10^{-7}	2.831×10^{-6}	2.922×10^{-6}	2.535×10^{-6}	1.517×10^{-6}	6.882×10^{-8}
Extracted from Li^+ MSD						
300	4.67×10^{-8}	1.66×10^{-7}	6.91×10^{-8}	1.58×10^{-8}	1.55×10^{-7}	8.38×10^{-9}
350	2.22×10^{-8}	6.41×10^{-7}	3.21×10^{-7}	5.70×10^{-8}	4.21×10^{-7}	5.54×10^{-9}
400	1.30×10^{-7}	1.60×10^{-6}	1.25×10^{-6}	5.79×10^{-7}	9.24×10^{-7}	4.32×10^{-0}
450	4.23×10^{-7}	2.99×10^{-6}	2.87×10^{-6}	2.49×10^{-6}	1.67×10^{-6}	5.48×10^{-8}
500	9.97×10^{-7}	5.04×10^{-6}	4.99×10^{-6}	4.54×10^{-6}	2.88×10^{-6}	2.49×10^{-7}
550	2.11×10^{-6}	7.31×10^{-6}	8.00×10^{-6}	7.18×10^{-6}	4.45×10^{-6}	4.07×10^{-7}
600	3.63×10^{-6}	1.09×10^{-5}	1.14×10^{-5}	1.08×10^{-5}	7.35×10^{-6}	9.49×10^{-7}
650	6.17×10^{-6}	1.48×10^{-5}	1.64×10^{-5}	1.55×10^{-5}	9.77×10^{-6}	1.95×10^{-6}
700	8.99×10^{-6}	1.94×10^{-5}	2.00×10^{-5}	2.00×10^{-5}	1.41×10^{-5}	3.47×10^{-6}
750	1.25×10^{-5}	2.52×10^{-5}	2.57×10^{-5}	2.54×10^{-5}	1.77×10^{-5}	5.62×10^{-6}
800	1.67×10^{-5}	3.05×10^{-5}	3.23×10^{-5}	3.22×10^{-5}	2.27×10^{-5}	8.28×10^{-6}

Table S4: Lithium diffusion coefficients (D_{Li}) for Li_6PS_5Br for each disorder configuration determined from machine learning-assisted molecular dynamics simulations. Values are listed in units of cm^2 s^{-1}. For $T < 500$ K, diffusion coefficients were determined both from the slope of the Li^+ MSD and also extrapolated from linear fits in the Arrhenius plot at higher simulation temperatures ($T = 500$ K to $T = 800$ K) for comparison.

T (K)	Config. 1	Config. 2	Config. 3	Config. 4	Config. 5	Config. 6
Extrapolated from Arrhenius						
300	7.955×10^{-10}	1.656×10^{-7}	1.499×10^{-7}	1.280×10^{-7}	7.730×10^{-8}	3.527×10^{-12}
350	7.827×10^{-9}	5.517×10^{-7}	5.132×10^{-7}	4.533×10^{-7}	2.789×10^{-7}	9.401×10^{-11}
400	4.349×10^{-8}	1.360×10^{-6}	1.291×10^{-6}	1.170×10^{-6}	7.300×10^{-7}	1.103×10^{-9}
450	1.650×10^{-7}	2.745×10^{-6}	2.647×10^{-6}	2.445×10^{-6}	1.543×10^{-6}	7.486×10^{-9}
Extracted from Li^+ MSD						
300	3.67×10^{-8}	6.20×10^{-8}	9.67×10^{-10}	8.06×10^{-8}	1.72×10^{-7}	2.42×10^{-8}
350	6.78×10^{-8}	3.34×10^{-7}	7.01×10^{-9}	3.79×10^{-7}	3.84×10^{-7}	3.12×10^{-9}
400	4.04×10^{-8}	1.07×10^{-6}	5.52×10^{-8}	1.10×10^{-6}	8.09×10^{-7}	6.56×10^{-9}
450	6.40×10^{-8}	2.62×10^{-6}	1.09×10^{-6}	2.35×10^{-6}	1.58×10^{-6}	2.40×10^{-8}
500	3.94×10^{-7}	4.83×10^{-6}	4.62×10^{-6}	4.28×10^{-6}	2.84×10^{-6}	2.46×10^{-8}
550	1.26×10^{-6}	7.59×10^{-6}	7.63×10^{-6}	7.21×10^{-6}	4.50×10^{-6}	1.62×10^{-7}
600	2.80×10^{-6}	1.11×10^{-5}	1.13×10^{-5}	1.12×10^{-5}	7.10×10^{-6}	3.88×10^{-7}
650	4.95×10^{-6}	1.55×10^{-5}	1.54×10^{-5}	1.53×10^{-5}	9.23×10^{-6}	9.56×10^{-7}
700	7.39×10^{-6}	2.03×10^{-5}	2.09×10^{-5}	1.95×10^{-5}	1.32×10^{-5}	1.96×10^{-6}
750	1.14×10^{-5}	2.69×10^{-5}	2.55×10^{-5}	2.57×10^{-5}	1.77×10^{-5}	2.99×10^{-6}
800	1.54×10^{-5}	3.12×10^{-5}	3.29×10^{-5}	3.22×10^{-5}	2.08×10^{-5}	5.23×10^{-6}

Table S5: Lithium diffusion coefficients (D_{Li}) for Li_6PS_5I for each disorder configuration determined from machine learning-assisted molecular dynamics simulations. Values are listed in units of cm^2 s^{-1}. For $T < 500$ K, diffusion coefficients were determined both from the slope of the Li^+ MSD and also extrapolated from linear fits in the Arrhenius plot at higher simulation temperatures ($T = 500$ K to $T = 800$ K) for comparison.

T (K)	Config. 1	Config. 2	Config. 3	Config. 4	Config. 5	Config. 6
Extrapolated from Arrhenius						
300	2.293×10^{-12}	1.055×10^{-7}	8.742×10^{-8}	9.796×10^{-8}	1.717×10^{-8}	2.129×10^{-13}
350	6.995×10^{-11}	3.665×10^{-7}	3.256×10^{-7}	3.602×10^{-7}	8.005×10^{-8}	7.210×10^{-12}
400	9.078×10^{-10}	9.326×10^{-7}	8.728×10^{-7}	9.565×10^{-7}	2.540×10^{-7}	1.012×10^{-10}
450	6.666×10^{-9}	1.928×10^{-6}	1.879×10^{-6}	2.045×10^{-6}	6.235×10^{-7}	7.900×10^{-10}
Extracted from Li^+ MSD						
300	4.61×10^{-8}	7.74×10^{-8}	9.12×10^{-8}	7.83×10^{-8}	7.28×10^{-8}	2.91×10^{-8}
350	6.78×10^{-8}	3.33×10^{-7}	3.14×10^{-7}	3.46×10^{-7}	1.97×10^{-7}	6.06×10^{-8}
400	9.13×10^{-9}	8.71×10^{-7}	8.14×10^{-7}	8.67×10^{-7}	4.04×10^{-7}	7.76×10^{-8}
450	1.01×10^{-8}	1.83×10^{-6}	1.85×10^{-6}	2.04×10^{-6}	7.68×10^{-7}	4.30×10^{-8}
500	2.13×10^{-8}	3.46×10^{-6}	3.37×10^{-6}	3.66×10^{-6}	1.32×10^{-6}	1.00×10^{-8}
550	1.77×10^{-7}	5.58×10^{-6}	6.02×10^{-6}	6.31×10^{-6}	2.29×10^{-6}	5.58×10^{-9}
600	4.20×10^{-7}	8.10×10^{-6}	8.46×10^{-6}	9.54×10^{-6}	3.64×10^{-6}	3.43×10^{-8}
650	1.09×10^{-6}	1.15×10^{-5}	1.26×10^{-5}	1.32×10^{-5}	5.51×10^{-6}	1.45×10^{-7}
700	1.92×10^{-6}	1.56×10^{-5}	1.70×10^{-5}	1.76×10^{-5}	8.21×10^{-6}	2.83×10^{-7}
750	3.64×10^{-6}	1.96×10^{-5}	2.17×10^{-5}	2.34×10^{-5}	1.11×10^{-5}	6.43×10^{-7}
800	5.94×10^{-6}	2.45×10^{-5}	2.73×10^{-5}	2.90×10^{-5}	1.48×10^{-5}	1.26×10^{-6}

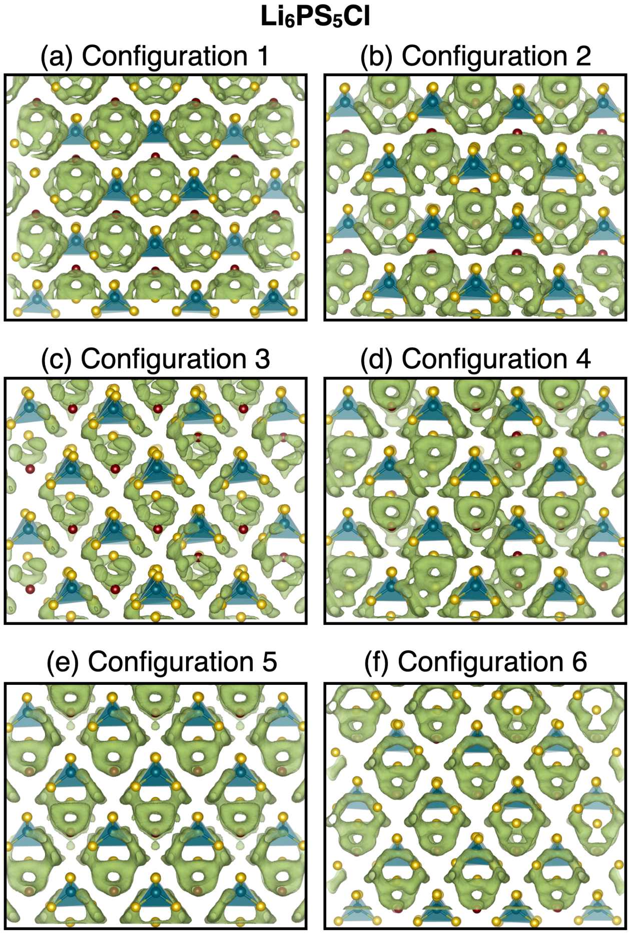

Figure S25: Lithium density distributions for Li_6PS_5Cl for each configuration of anion disorder extracted from the molecular dynamics simulations at $T = 300$ K.

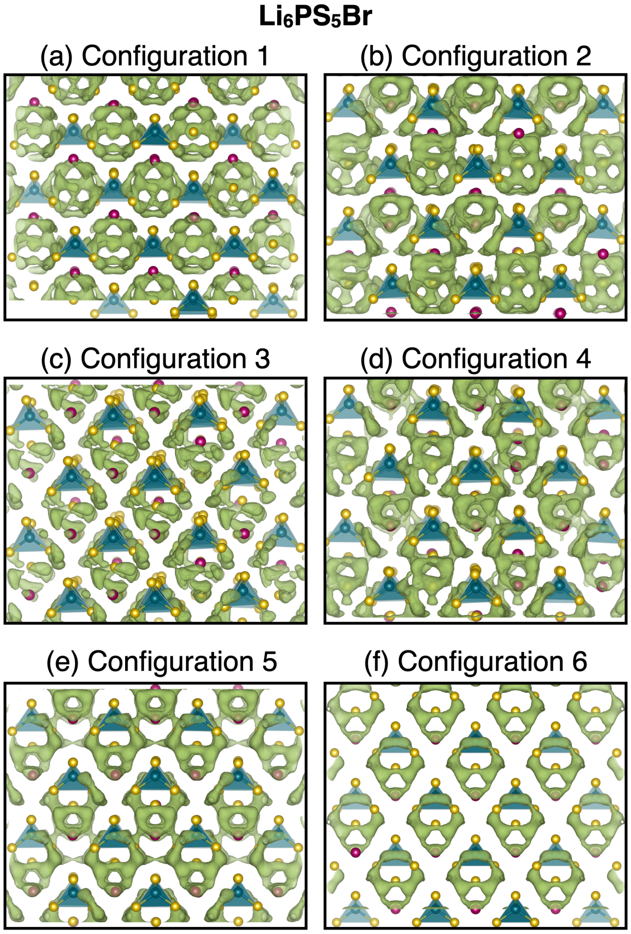

Figure S26: Lithium density distributions for Li_6PS_5Br for each configuration of anion disorder extracted from the molecular dynamics simulations at $T = 300$ K.

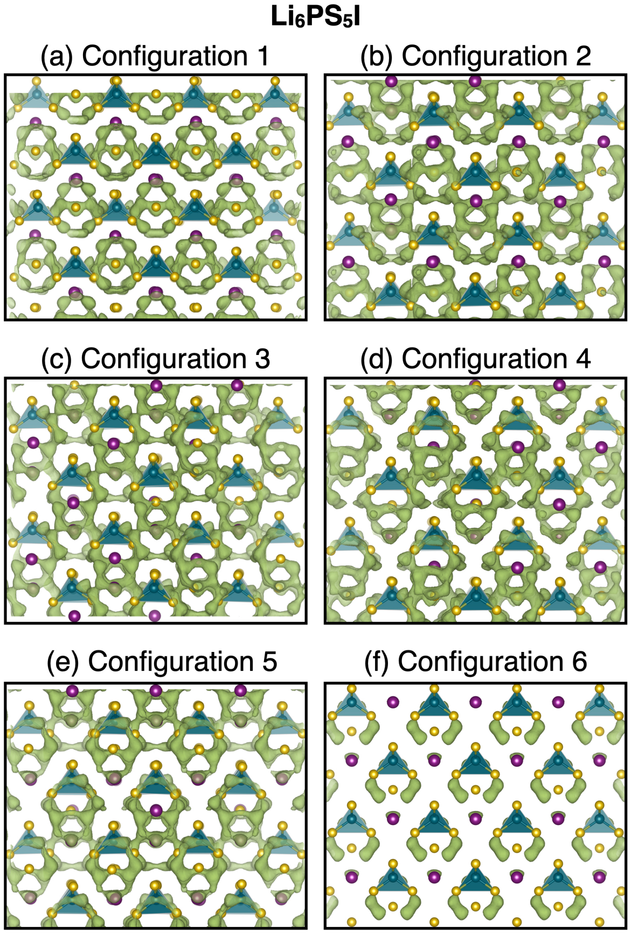

Figure S27: Lithium density distributions for Li_6PS_5I for each configuration of anion disorder extracted from the molecular dynamics simulations at $T = 300$ K.

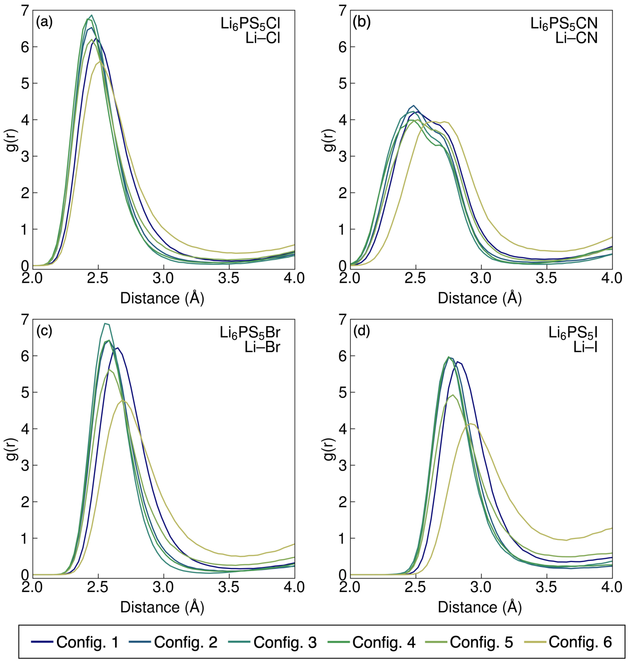

Figure S28: Radial distribution function for Li–X distances for (a) Li_6PS_5Cl, (b) Li_6PS_5CN, (c) Li_6PS_5Br, and (d) Li_6PS_5I for each configuration of anion disorder determined from MD simulations at $T = 300$ K.

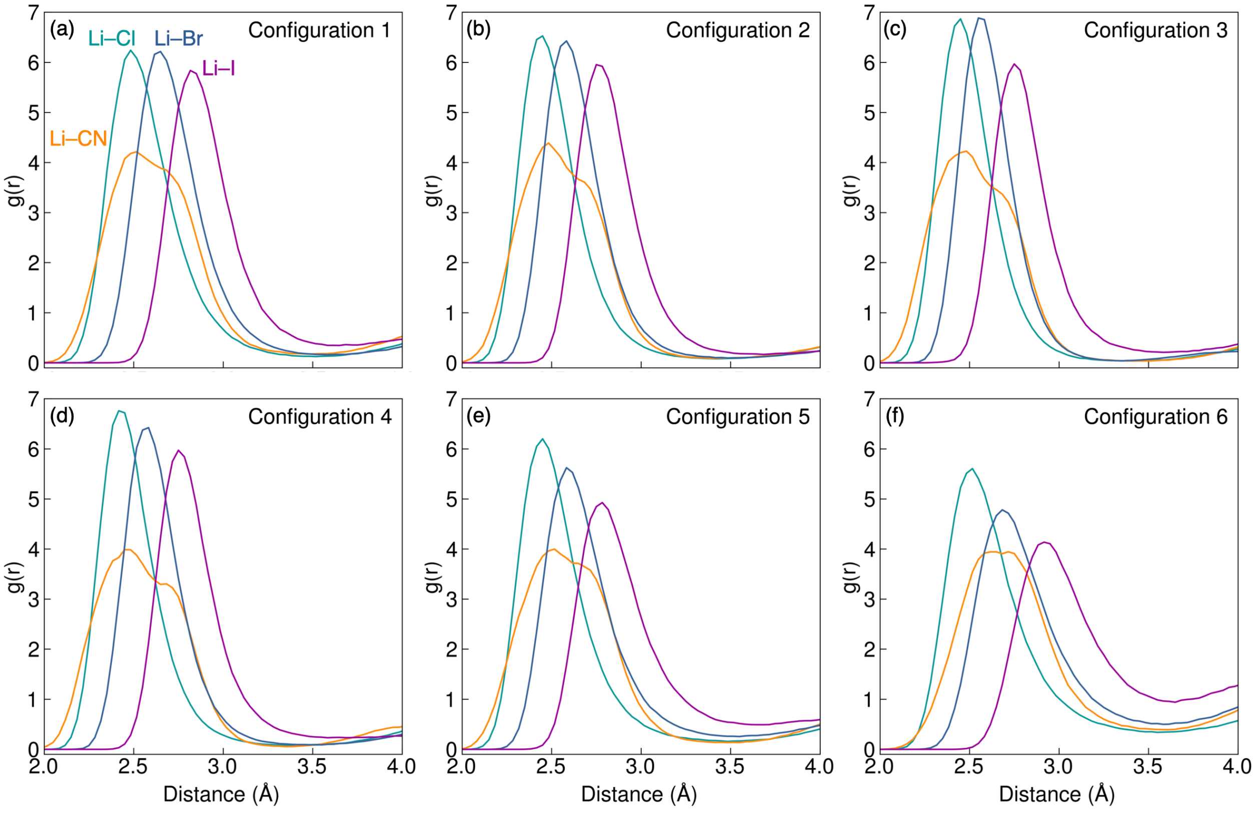

Figure S29: Radial distribution function for Li–X distances for each configuration of anion disorder determined from MD simulations at $T = 300$ K.

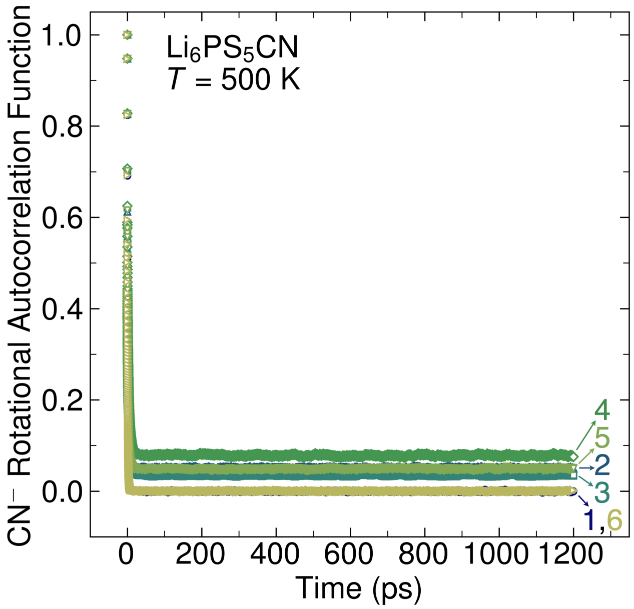

Figure S30: The rotational autocorrelation function for cyanide ions calculated for all Configurations of anion site disorder from MD simulations performed at T = 500 K. Each Configuration number is labeled adjacent to the respective curves.

References

(1) Morgan, B. J.; Madden, P. A. Absence of a space-charge-derived enhancement of ionic conductivity in β/γ-heterostructured 7H- and 9R-AgI. *J. Phys. Condens. Matter* **2012**, *24*, 275303.

(2) Morgan, B. J. Mechanistic Origin of Superionic Lithium Diffusion in Anion-Disordered Li_6PS_5X Argyrodites. *Chem. Mater.* **2021**, *33*, 2004–2018.
